# Allenylidene Phosphonium Ion: An Isoelectronic Phosphorus Analogue of [3]Cumulene

**DOI:** 10.1002/anie.202502201

**Published:** 2025-04-10

**Authors:** Lucas C. Torres, Pawel Löwe, Avik Bhattacharjee, Maike B. Röthel, Michael Seidl, Jesse LeBlanc, Klaus Wurst, Fabian Dielmann, Christopher B. Caputo

**Affiliations:** ^1^ Department of Chemistry York University 4700 Keele St Toronto ON M3J 1P3 Canada; ^2^ Institute of General, Inorganic and Theoretical Chemistry Universität Innsbruck Innrain 80–82 Innsbruck 6020 Austria; ^3^ Institute of Inorganic and Analytical Chemistry Westfälische Wilhelms‐Universität Münster 48149, Corrensstrasse 30 48149 Münster Germany

**Keywords:** Cumulene, Cycloaddition, Lewis acids, Phosphorus, Trigonal planar phosphonium cations

## Abstract

Herein, we disclose the synthesis, characterization, and bonding analysis of a crystalline allenylidene phosphonium ion ([R_2_P═C═C═CAr_2_]^+^) [**2**]^+^ (R = 1,3‐diisopropylphenylimidazolin‐2‐ylidenamino, CAr_2_ = 9‐fluorenylidene), an isolobal analogue of [3]cumulenes. The cation was prepared *via* methoxide ion abstraction of an alkynyl phosphine **1**. Electron rich *N*‐heterocyclic imine substituents and a π conjugated fluorenyl scaffold together stabilize the resulting three‐coordinate phosphonium center of [**2**]^+^. [**2**]^+^ features a trigonal planar phosphorus atom which is electrophilic, as evidenced experimentally by the preparation of a Lewis acid‐base adduct with 4‐dimethylaminopyridine, and fluoride ion affinity calculations. Crystallographic and computational investigations of [**2**]^+^ support a structure with considerable P═C double bond character. Furthermore, this P═C double bond participates in a thermally reversible intramolecular dearomative [2 + 2] cycloaddition with an arene moiety of one of its supporting 1,3‐diisopropylphenylimidazolin‐2‐ylidenamino substituents to give a tetracyclic isomer [**2^cyclo^**]^+^. The isolation of a free [R_2_P═C═C═CAr_2_]^+^ draws further parallels between the properties and reactivity of low‐coordinate phosphorus compounds and related carbon compounds.

## Graphical abstract

We report the first isolobal and isoelectronic incorporation of a phosphorus atom into a [3]cumulene. This trigonal planar phosphonium allenylidene demonstrates exceptional Lewis acidity via a π* acceptor orbital and exhibits unique reactivity through a reversible intramolecular dearomative [2 + 2] cycloaddition reaction.

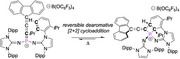

## Introduction

The isolation of p‐block element versions of carbon compounds continues to be a highly active research topic. Within this area, low‐coordinate and unsaturated phosphorus compounds which mimic the electronic or bonding situation of carbon‐based small molecules have been the focus of intense investigation for decades. Striking parallels in the molecular chemistry of phosphorus and carbon reflect the diagonal relationship shared between the two elements; derived from their comparable electronegativities and similar propensity to form element‐element multiple bonds.^[^ ^1^, ^2^ ^]^

**Figure 1:**
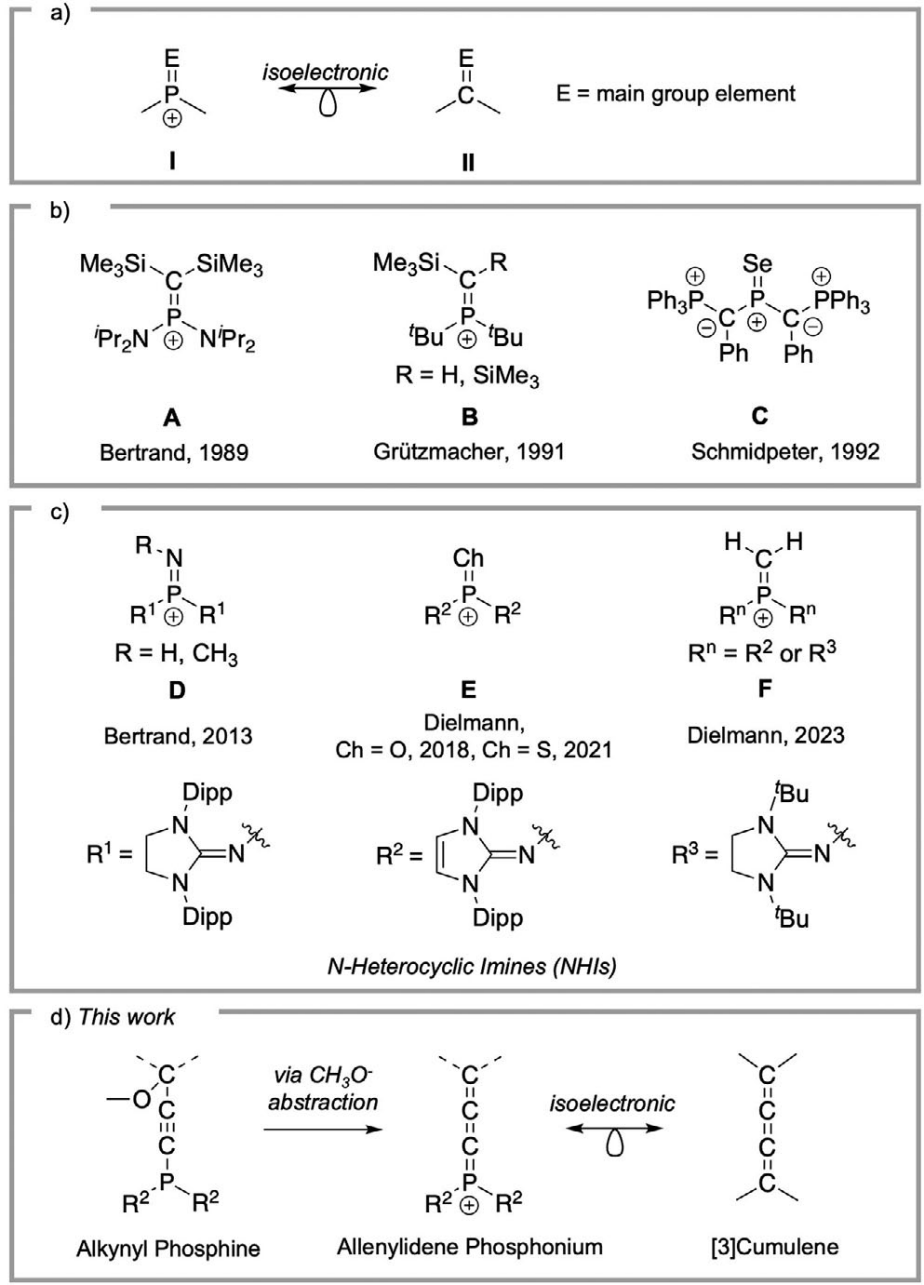
a) Isolobal/isoelectronic relationship between trigonal planar phosphorus cations I and sp^2^‐carbon compounds II. b) Isolable trigonal planar phosphorus cations **A‐C**. c) NHI‐stabilized trigonal planar phosphorus cations **D‐F**. d) This work.

Trigonal‐planar phosphonium cations [R_2_P═E]^+^ (Figure 1, **I**) are a fascinating class of phosphorus(V) species that represent isolobal and isoelectronic analogues of organic molecules which contain *s*p^2^‐carbon atoms (R_2_C═E) (Figure 1, **II**).^[^ ^3^, ^4^ ^]^ While R_2_C═E compounds like imines, olefins, and (thio)carbonyls are ubiquitous in nearly all aspects of chemistry, examples of [R_2_P═E]^+^ entities are limited as their syntheses pose a considerable challenge. This is exacerbated by the fact that [R_2_P═E]^+^ compounds exhibit poor thermodynamic stability and are prone to dimerization.^[^ ^5^, ^6^, ^7^ ^]^ Despite this, the feasibility of isolating compounds with [R_2_P═E]^+^ structural elements was demonstrated over 30 years ago by the working groups of Bertrand **A**,^[^ ^8^ ^]^ Grützmacher **B**,^[^ ^9^, ^10^, ^11^ ^]^ and Schmidpeter **C**,^[^ ^12^ ^]^ although limited to select bonding motifs, namely methylene phosphonium [R_2_P═CR_2_]^+^,^[^ ^8^, ^9^, ^10^ ^]^ selenophosphonium [R_2_P═Se]^+^,^[^ ^12^ ^]^ and phosphanylphosphenium [R_2_P═PR]^+^ cations (Figure 1b).^[^ ^11^ ^]^ Within the last decade, a new era of [R_2_P═E]^+^ compounds have been described, attributed to innovations in the design of stabilizing substituents appropriate for accessing electron deficient main‐group centers. In this regard, *N*‐heterocyclic imine (NHI) groups, which possess robust π‐donating properties accompanied by a substantial steric profile, can be leveraged to impart thermodynamic, and kinetic stability to the P center of [R_2_P═E]^+^ salts.^[^ ^13^ ^]^ By exploiting this molecular design, the first examples of iminophosphonium ions **D** [R_2_P═N−R]^+^,^[^ ^14^ ^]^ oxo/thio‐phosphonium ions **E** [R_2_P═O]^+^/[R_2_P═S]^+^,^[^ ^15^, ^16^ ^]^ and terminal methylene phosphonium ions **F** [R_2_P═CH_2_]^+^ have been prepared,^[^ ^17^ ^]^ which represent genuine phosphorus analogues of imines, (thio)carbonyls, and terminal olefins, respectively (Figure 1c).

With the [R_2_P═E]^+^/ R_2_C═E isolobal analogy in mind, a number of “phosphorus mimics” of unsaturated carbon compounds can be approached with this new toolkit. Of particular interest to our groups, and yet to be described in the literature, are phosphorus analogues of [*n*]cumulenes (*n* = # of double bonds). [*n*]Cumulenes are an allotrope of carbon characterized by contiguous double bonds in their structure and have attracted considerable interest for numerous applications in materials science.^[^ ^18^, ^19^, ^20^, ^21^, ^22^, ^23^ ^]^ For synthetic chemists, [*n*]cumulenes offer a wealth of potential transformations which are afforded through their π electron rich framework.^[^ ^18^, ^19^, ^20^, ^21^, ^22^, ^23^, ^24^ ^]^ [3]Cumulenes are the simplest, most stable, and synthetically accessible representatives of the cumulene family (Figure 1d). Phosphorus incorporation into a [3]cumulene system has been explored previously by Bestmann, in the form of neutral phosphacumulene ylides R_3_P═C═C═CR_2_.^[^ ^25^, ^26^ ^]^ Contrary to what their name suggests, phosphacumulene ylides have tetracoordinate σ^4^ *λ* ^5^ P atoms, and lack the correct orbital configuration and structural features characteristic of cumulenes (i.e. a linear arrangement of sp hybridized atoms terminated by sp^2^ hybridized atoms). In this article, we explore the isolobal displacement of a single sp^2^ carbon atom of a [3]cumulene with a trigonal planar phosphonium cation to afford an allenylidene phosphonium [R_2_P═C═C═CAr_2_]^+^ (Figure 1d), representing the first “genuine” phosphorus analogue of such a system.

## Results and Discussion

**Figure 2:**
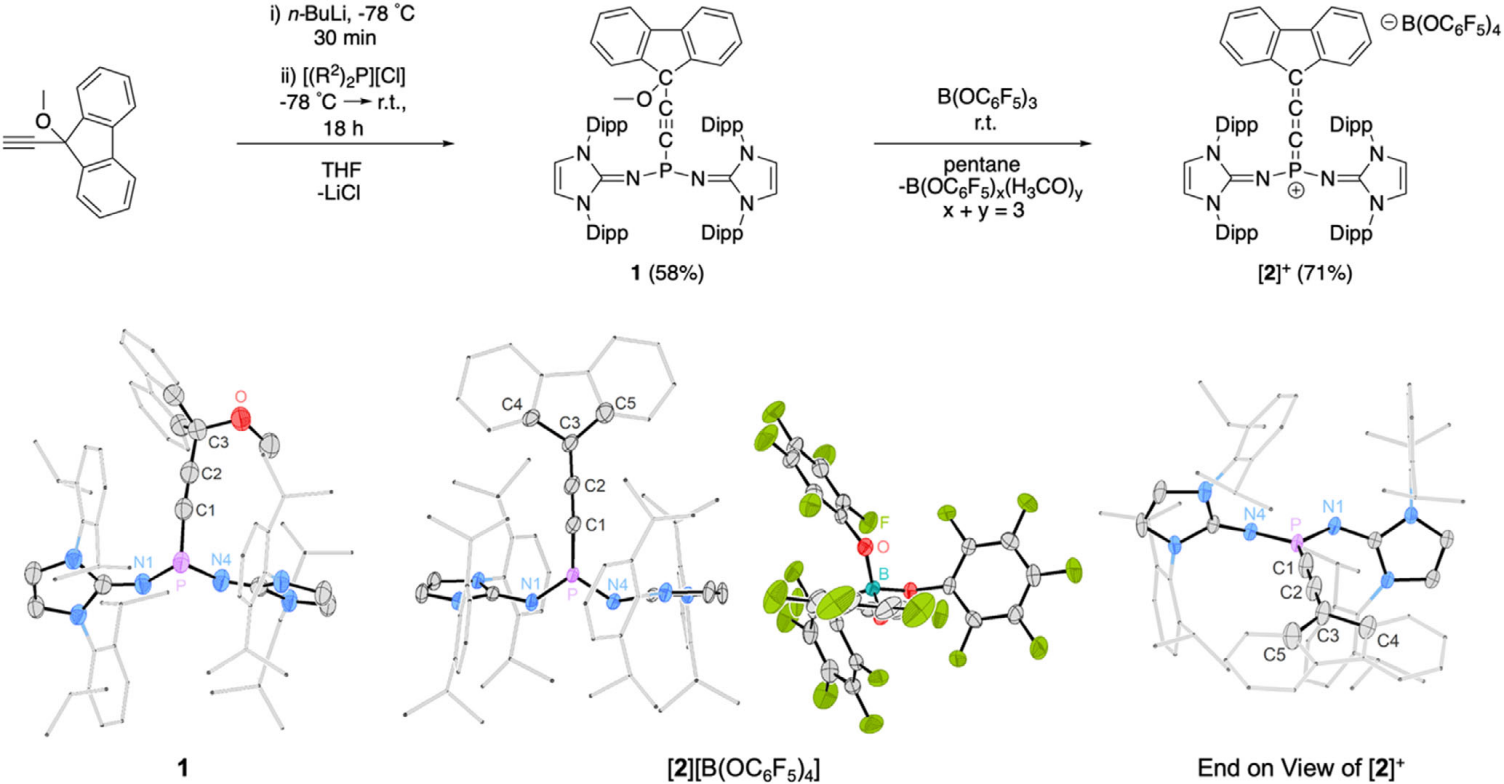
Synthesis of bis(imino)alkynylphosphine **1** and allenylidene phosphonium [**2**][B(OC_6_F_5_)_4_] (top). Solid‐state structure of 1 (bottom‐left). Solid‐state structure of [**2**][B(OC_6_F_5_)_4_] (bottom‐center). End‐on perspective for the solid‐state structure of [**2**]**^+^**, with the [B(OC_6_F_5_)_4_]^−^ anion omitted (bottom‐right). In all cases, thermal ellipsoids are shown at 50% probability. For clarity, hydrogen atoms are omitted. The Dipp groups and fluorenyl motifs are shown in wireframe. Selected atomic distances [Å] and angles [°]: **1**: P−C1 1.810(3), P─N1 1.673(2), P─N4 1.689(2), C1─C2 1.217(4), C2─C3 1.484(4), C3─O 1.436(4), N1─P─C1 104.75(12), N4─P─C1 96.23(12), N1─P─N4 96.75(11). [**2**][B(OC_6_F_5_)_4_]: P─C1 1.624(4), C1─C2 1.252(5), C2─C3 1.332(5), P─N1 1.560(3), P─N4 1.557(3), P─C1─C2 170.3(4), C1─C2─C3 179.3(4), N1─P─N4 112.55(15), N1─P─C1 123.78(17), N4─P─C1 123.37(17), C2─C3─C4 125.3(4), C2─C3─C5 127.7(3), C4─C3─C5 106.5(3).

In pursuit of an allenylidene phosphonium cation, we reasoned its synthesis could be approached in a similar manner to that of cationic transition metal allenylidene complexes.^[^ ^27^, ^28^, ^29^, ^30^, ^31^ ^]^ In particular, we were inspired by the work of Widenhoefer and co‐workers with their Lewis acid mediated synthetic strategy to access cationic (allenylidene)carbene gold complexes from their respective acetylide precursors.^[^ ^28^ ^]^ This led us to devise a methodology which employs alkynyl phosphines of the form R_2_P─C≡C─C(OCH_3_)Ar_2_, and utilize a Lewis acid as a methoxide ion (CH_3_O^−^) abstraction agent to generate the desired trigonal planar phosphonium cation [R_2_P═C═C═CAr_2_]^+^ (Figure 1).^[^ ^32^, ^33^ ^]^ Our group had previously attempted to isolate [R_2_P═C═C═CAr_2_]^+^ *via* this approach, with *P*‐aryl‐ or *P*‐alkyl‐ substituted precursors R_2_P─C≡C─C(OCH_3_)Ar_2_ (R═Ph, Cy, Mes), and B(C_6_F_5_)_3_ as a CH_3_O^−^ abstracting agent, but we were ultimately unsuccessful.^[^ ^32^ ^]^ Complementary computational modelling provided in the study did however support the existence of [R_2_P═C═C═CAr_2_]^+^ as fleeting, kinetically accessible, intermediates. However, to isolate the desired compound, significant changes to the molecular design would be required to stabilize this intermediate. In this present study, we sought out bis(imino)alkynyl phosphine **1** as an appropriate precursor to access an allenylidene phosphonium salt, given that decoration of the P atom with bulky π‐donating groups has been the key to the isolation of various [R_2_P═E]^+^ compounds.^[^ ^14^, ^15^, ^16^, ^17^ ^]^ Additionally, the stability of carbon cumulenes is improved when such species are end capped with aryl substituents as they can π conjugate with the cumulene core.^[^ ^18^, ^19^, ^20^, ^21^, ^22^, ^23^ ^]^ With this in mind, we opted to terminate alkynyl phosphine **1** with a fluorenyl scaffold in hopes that this structural motif might offer additional stabilization to the target [R_2_P═C═C═CAr_2_]^+  ^cation by facilitating π conjugation and charge delocalization throughout the cumulene. **1** was readily prepared in a reaction of 9‐ethynyl‐9‐methoxy‐9*H*‐fluorene with *n*‐butyl lithium at −78 °C, with the subsequent addition of the phosphenium chloride [(R^2^)_2_P]Cl (R^2^ = 1,3‐diisopropylphenylimidazolin‐2‐ylidenamino, NIDipp).^[^ ^17^, ^34^ ^]^ Compound **1** was isolated in a yield of 58% and crystallized from a concentrated *n*‐hexane solution. Characteristically, the phosphine shows a singlet at *δ* = +51.9 ppm in the ^31^P NMR spectrum. The ^13^C NMR resonances corresponding to the α, β, and γ carbons of **1** were identified as features at 94.9 ppm (^1^ *J*_PC_ = 65 Hz), 92.0 ppm (^2^ *J*_PC_ =  8 Hz), and 81.2 ppm (^3^ *J*_PC_ = 2 Hz), respectively. A single‐crystal X‐ray diffraction (SCXRD) study indicated that **1** has an expected pyramidalized geometry at the N_2_PC unit (sum of angles: 298°) and exhibits a considerable degree of steric crowding around the phosphorus center (Figure 2). The P─C1 bond length in **1** is 1.810(3) Å, in the range for a P─C single bond, and consistent with other previously reported alkynyl phosphines.^[^ ^32^, ^33^, ^35^ ^]^ The C1─C2 bond is 1.217(4) Å, consistent with a C─C triple bond, while the C2─C3 bond length agrees with a Csp─Csp^3^ single bond (1.484(4) Å).

The allenylidene phosphonium cation [**2**]**^+^** was prepared by CH_3_O^−^ abstraction from **1** using the borate ester B(OC_6_F_5_)_3_.^[^ ^36^ ^]^ Carrying out this reaction in either pentanes or *n*‐hexane instantaneously resulted in the precipitation of a bright red solid, which was subsequently characterized by multinuclear NMR spectroscopy at −20 °C (Note: low temperature characterization was undertaken as NMR experiments at room temperature suggested [**2**]^+^ undergoes a subsequent transformation (vide infra)). The main feature of the NMR spectra of [**2**]**^+^** are the ^13^C resonances corresponding to the α, β, and γ cumulene carbons, which show doublets at *δ* = 99.1 ppm (^1^ *J*_PC_ = 263 Hz), 161.9 ppm (^2^ *J*_PC_ = 34 Hz), and 94.6 ppm (^3^ *J*_PC_ = 31 Hz), respectively. Compared to **1**, the *J*_PC_ coupling constants for carbons α−γ are all roughly one order of magnitude larger, consistent with a change in oxidation state from P^III^ to P^V^, as well as an increase in s character of the phosphorus‐carbon bond in [**2**]^+^. Despite a change in oxidation state, the ^31^P NMR chemical shift of [**2**]**^+^** (*δ* = + 43.9 ppm) is only shifted slightly to lower frequencies relative to phosphine **1**.^[^ ^14^, ^15^, ^16^, ^17^ ^]^ Interestingly, heteronuclear NMR experiments suggested the identity of the anion to be [B(OC_6_F_5_)_4_]^−^, evidenced by a sharp signal at +1.6 ppm in the ^11^B {^1^H} NMR spectrum, and three distinct resonances observed in the ^19^F {^1^H} NMR (*δ* = −156.9, −167.9, and −171.2 ppm). While the reaction of **1** and B(OC_6_F_5_)_3_ was anticipated to give [**2**]^+^ paired with a methoxy‐borate [H_3_CO−B(OC_6_F_5_)_3_]^−^ counterion, the exclusive presence of [B(OC_6_F_5_)_4_]^−^ implies that substituent redistribution occurs during the reaction, liberating neutral borate esters with the form (B(OC_6_F_5_)*_x_* (H_3_CO)*_y_*) (*x* + *y* = 3)^[^ ^37^, ^38^, ^39^ ^]^ After purification by washing with pentane or hexanes, [**2**][B(OC_6_F_5_)_4_] was obtained in yields up to 71%. The isolation of [**2**]^+^ was also initially attempted with other CH_3_O^−^ abstracting agents, including B(C_6_F_5_)_3_, TMSOTf, and Al(C_6_F_5_)_3_, but such reactions either gave the target cation with poorly defined anionic components, or were unselective.

**Scheme 1:**
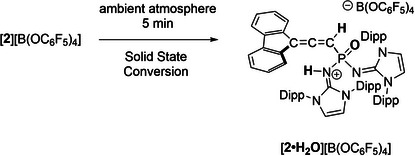
Solid‐state hydrolysis of crystalline [**2**][B(OC_6_F_5_)_4_] to [**2•H_2_O**][B(OC_6_F_5_)_4_] (see Supporting Information, Section 2.9).

Storing a CH_2_Cl_2_/pentane solution of [**2**][B(OC_6_F_5_)_4_] at −40 °C afforded crystals suitable for a SCXRD study (Figure 2). In the molecular structure, the phosphorus atom has a planar coordination environment (sum of angles: 360°). The formulation of the anion was confirmed to be [B(OC_6_F_5_)_4_]^−^ and is well separated from the cationic component. [**2**]**^+^** deviates slightly from the idealized linearity of 180° for a [3]cumulene‐type system, with bending about the P terminus (∠P─C1─C2 angle of 170.3°). The phosphorus‐bound NHI substituents and terminal carbon‐bound aryl rings are slightly out of plane, with a measured N1─N4─C4─C5 twist angle of 7.9°. The P─C1 bond distance of 1.624(4) Å is relatively short for a methylene phosphonium analogue,^[^ ^40^, ^41^, ^42^, ^43^ ^]^ and is comparable in length to the P─C bond of the NHI substituted terminal methylene phosphonium ions **F** (R = R^3^, 1.621(5) Å; R = R^2^, 1.619(3) Å).^[^ ^17^ ^]^ The C1─C2 distance of [**2**]**^+^** is 1.252(5) Å, which is short for a C─C double bond but typical for the internally bonded sp‐hybridized carbon atoms of [3]cumulenes.^[^ ^18^, ^19^, ^20^, ^21^, ^22^, ^23^ ^]^ The C2─C3 bond length is 1.332(5) Å which agrees with a typical terminal cumulene Csp─Csp^2^ double bond. The P─N bonds (average distance of 1.559 Å) are in the range of P─N double bonds (1.50−1.60 Å),^[^ ^44^ ^]^ which reflects strong π‐donation from the NHI substituents to the phosphorus atom. We found that in a CD_2_Cl_2_ solution, [**2**]**^+^** decomposes rapidly when exposed to air, which is indicated by an immediate color change from red to yellow. Solid samples of [**2**][B(OC_6_F_5_)_4_] degrade more slowly. Remarkably, the red single crystals of [**2**][B(OC_6_F_5_)_4_] changed color to yellow after brief exposure to air, while retaining their crystallinity. Although the data obtained by an SCXRD study of a yellow crystal was of low quality, it revealed the connectivity of the hydrolysis product to be [**2•H_2_O**][B(OC_6_F_5_)_4_], which was corroborated by multinuclear NMR spectroscopy and high‐resolution mass spectrometry (HRMS) (Supporting Information, Section 2.9). Hydrolysis of [**2**]**^+^** to [**2•H_2_O**][B(OC_6_F_5_)_4_] occurs in analogous manner to the hydrolysis of the iminophosphonium cations reported by Bertrand and co‐workers (Scheme 1).^[^ ^14^ ^]^

**Figure 3:**
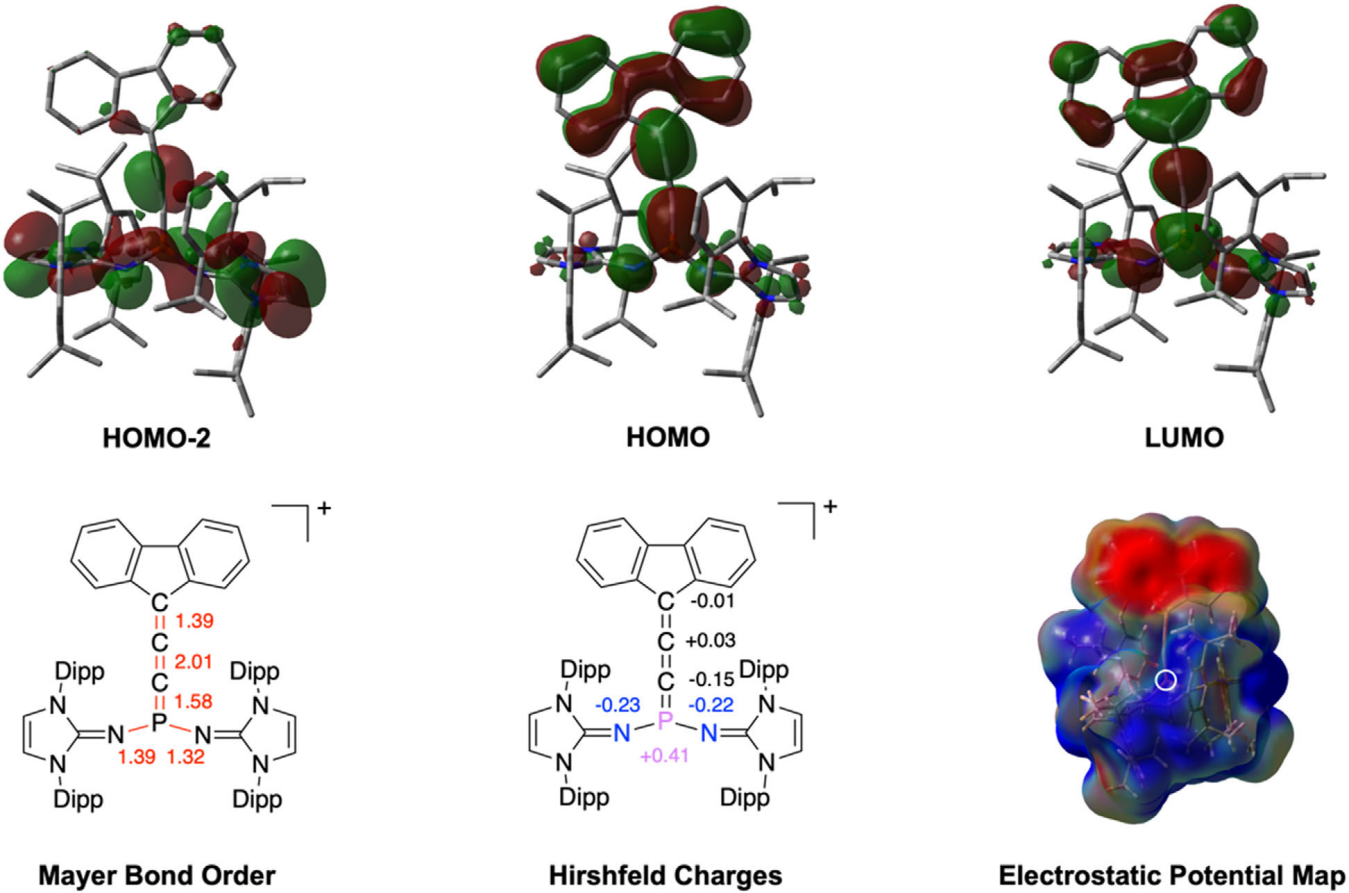
Selected molecular orbitals of [**2**]**^+^**  (HOMO‐2, HOMO and LUMO) at the B3LYP−D3BJ/def2−TZVP level of theory (top). Calculated Mayer bond order analysis (bottom left) and Hirshfeld charges (bottom‐center) of [**2**]**^+^**. Electrostatic potential map of [**2**]**^+^**  (bottom‐right).

To gain insight into the electronic structure of [**2**]^+^, UV‐Vis spectroscopy was undertaken. This compound showed a *λ*_max_ at 483 nm which closely resembles with the absorption properties of analogous all carbon [3]cumulenes (Figure S17).^[^ ^18^, ^19^, ^20^, ^21^, ^22^, ^23^ ^]^ This was further corroborated by TD‐DFT calculations (B3LYP/6‐311+G(d)) with a simulated absorption at 478 nm, corresponding to a HOMO‐LUMO transition (Figure S79 and Table S7). Moreover, the redox behavior of [3]cumulenes has been well established in the literature.^[^ ^18^, ^19^, ^20^, ^21^, ^22^, ^23^ ^]^ We observe at least one irreversible reduction and one irreversible oxidation event in cyclic voltammetry at −0.75 V and +0.62 V, respectively vs Ag/AgCl (Figure S18), suggesting that the allenylidene phosphonium moiety is redox active. Additionally, quantum chemical calculations were performed at the B3LYP−D3BJ/def2−TZVP level of theory (see Supporting Information Section 4).^[^ ^45^, ^46^, ^47^ ^]^ The optimized geometry of [**2**]^+^ is in excellent agreement with the corresponding solid‐state structure. Density Functional Theory (DFT) calculations revealed that the highest occupied molecular orbital (HOMO) reflects the P─C1 and C2─C3 π−bonding orbitals, as well as the delocalized π system of the fluorenyl entity. The HOMO−2 corresponds to the orthogonal C1─C2 π−bonding orbital, as well as the π system and exocyclic nitrogen lone pair of the NHI substituents. The lowest unoccupied molecular orbital (LUMO) represents the π^∗  ^orbital of the cumulene unit, while the in‐plane π^∗ ^is reflected in the LUMO+2. Antibonding combinations in the fluorenyl group are also represented in the LUMO depiction. Mayer bond orders for P─C1 (1.58), C1─C2 (2.01), and C2─C3 (1.39) indicate significant π−bonding character along the PCCC motif, especially with respect to the C1─C2 unit. Hirshfeld charges analyses revealed that the P atom (+0.41) is the most electrophilic site in [**2**]^+^ and the *α*‐carbon carries a negative charge (−0.15), suggesting a polarized P^δ+^─C^δ−^ bond. The *β*‐carbon (+0.03) and *γ*−carbon (−0.01) are deemed neutral. The electrostatic potential map illustrates that [**2**]^+^ is highly polarized, with an electron‐depleted P terminus, and an electron‐rich C terminus (Figure 3). With respect to the Lewis acidity of [**2**]^+^, fluoride ion affinities (FIA) were determined using Christie's method, and computed in both gas phase as well as using a polarizable continuum model for CH_2_Cl_2_.^[^ ^48^ ^]^ The gas phase FIA of [**2**]^+^ suggested a high degree of electrophilicity (655 kJ·mol^−1^), and is comparable to previously reported three‐coordinate oxophosphonium cations, [(NIDipp)_2_PO]^+^ (634 kJ·mol^−1^), and [(NIDipp)(IDippCH)PO]^+^ (618 kJ·mol^−1^)^[^ ^15^ ^]^ but lower than reported values for other monocationic phosphorus Lewis super acids like Stephan's organofluorophosphonium [(C_6_F_5_)_3_PF]^+^ (FIA: 795 kJ·mol^−1^),^[^ ^15^, ^49^ ^]^ as well as Greb's catecholato‐derived phosphonium salts [P(cat)_2_]^ +^ (FIA: 677−845 kJ·mol^−1^).^[^ ^50^ ^]^ Accounting for dampening of the FIA by CH_2_Cl_2_ solvation (366 kJ·mol^−1^), [**2**]^+^ is still considered a strong electrophile.

**Figure 4:**
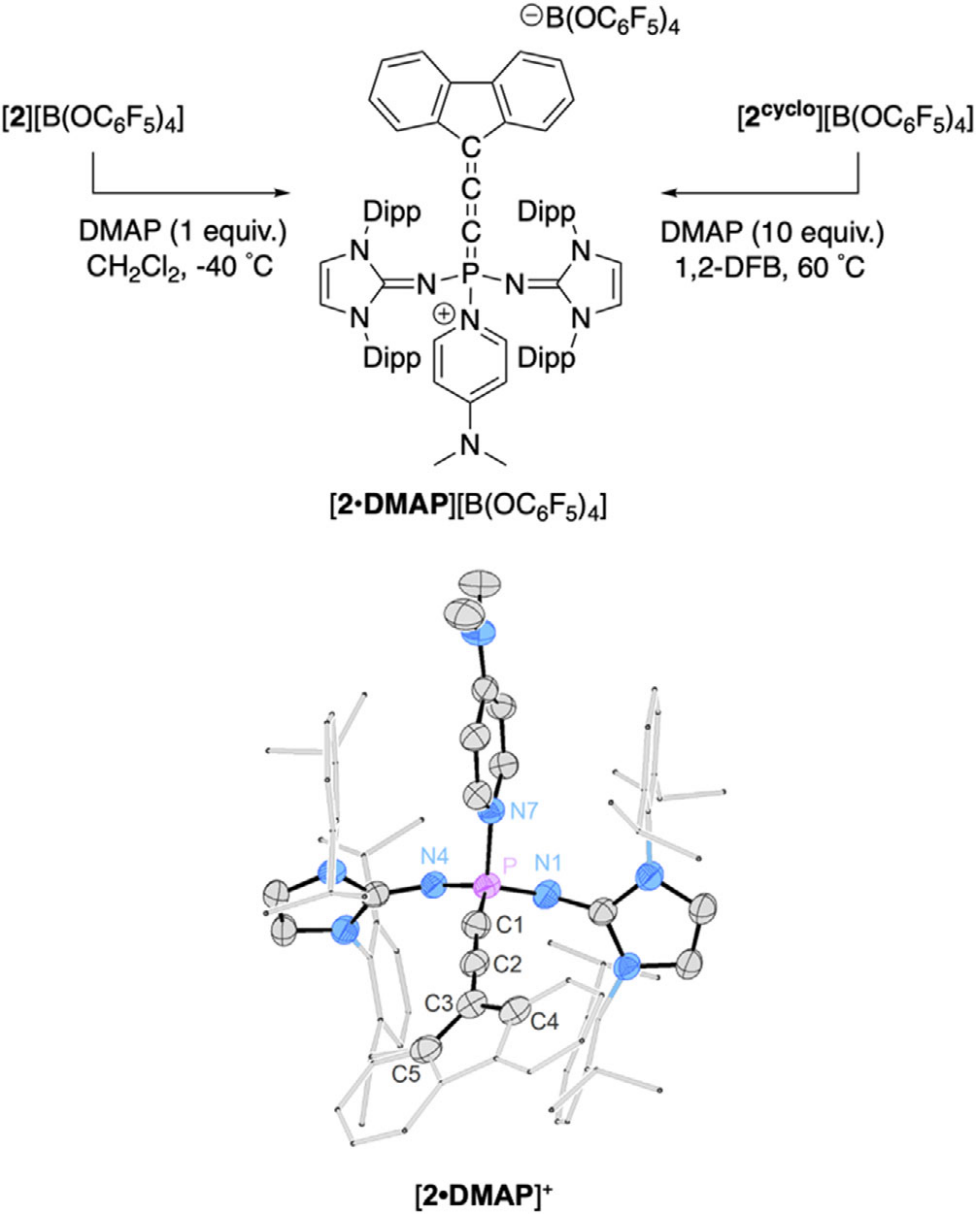
Two Independent syntheses of [**2•DMAP**][B(OC_6_F_5_)_4_] (top). Thermally induced cycloreversion of [**2^cyclo^**][B(OC_6_F_5_)_4_] in solution using DMAP as a trapping reagent (top right). Molecular structure of [**2•DMAP**]^+^ with the [B(OC_6_F_5_)_4_] anion excluded (bottom). Thermal ellipsoids are shown at 50% probability. For clarity, hydrogen atoms are omitted. The Dipp groups and fluorenyl motif are shown in wireframe. Selected atomic distances [Å] and angles [°]: P─C1 1.677(3). C1─C2 1.227(4), C2─C3 1.385(4), P─N1 1.561(3), P─N4 1.567 (2), P─N7 1.787(2), P─C1─C2 166.2(3), C1─P─N1 118.19(14), C1─P─N4 116.69(14), N1─P─N4 108.82(13), N4─P─N7 105.23(11), N1─P─N7 104.42(12), C1─P─N7 101.56(13).

It was next attempted to obtain an effective Lewis acidity measure for [**2**]^+^ *via* the Gutmann‐Beckett method,^[^ ^51^, ^52^ ^]^ but this proved to be inconclusive. The ^31^P NMR spectrum of a reaction between Et_3_PO and [**2**]^+^ in CD_2_Cl_2_ suggests that an unwanted reaction occurs that appears to involve cleavage of the P─O bond (Supporting Information Section 2.8). As an alternative for demonstrating the Lewis acidic character, [**2**]^+^ was treated with the Lewis base 4−dimethylaminopyridine (DMAP).^[^ ^14^, ^15^ ^]^ Combining a CH_2_Cl_2_ solution of [**2**][B(OC_6_F_5_)_4_] with DMAP at −40 °C gave the isolable donor‐acceptor compound [**2•DMAP**][B(OC_6_F_5_)_4_], which is a cationic σ^4^ *λ* ^5^ phosphacumulene with a ^31^P NMR chemical shift of *δ* = −30.4 ppm (^3^ *J*_PH_ = 8.2 Hz). [**2•DMAP**][B(OC_6_F_5_)_4_] was subjected to a SCXRD study, revealing an expected tetracoordinate phosphorus center, and slightly bent geometry about the bond angle of P1─C1─C2 of 166° (Figure 4). This data differs to previously reported neutral phosphacumulene ylides (R_3_P═C═C═X; X = CR_2_, O, S) which typically have considerably bent P1─C1─C2 angles.^[^ ^53^, ^54^, ^55^, ^56^, ^57^ ^]^ Compared to [**2**]^+^, [**2•DMAP**][B(OC_6_F_5_)_4_] has a longer P─C1 bond (1.677(3) Å), but this bond length is consistent with the P─C bond of aforementioned cumulated ylides. In the presence of acetonitrile with gentle heating, [**2**]^+^ was found to form the cationic azaphosphete [**3**]^+^ via polarized intermolecular [2 + 2] cycloaddition, which was confirmed by multinuclear and 2D NMR techniques, as well as by HRMS. [**3**]^+^ shows a singlet at *δ* = −18.7 ppm in the phosphorus NMR spectrum which appears at a lower frequency than [**2**]^+^ (*δ* = +43.9 ppm). The formation of [**3**]^+^ is supported by ^13^C NMR experiments, revealing three diagnostic deshielded doublets, one centered at 187.9 ppm (^2^ *J*_CP_ = 24 Hz) corresponding to the sp hybridized allene carbon, a second centered at 187.3 ppm (^2^ *J*_CP_ = 25 Hz) attributed to the nitrogen‐bound azaphosphete carbon atom, and a third at *δ* = 120.5 ppm (^1^ *J*_CP_ = 70 Hz) which reflects the sp^2^ carbon atom directly bound to phosphorus. The ^1^H NMR signal of the acetonitrile derived methyl group in [**3**]^+^ appears as a doublet at *δ* = 1.34 ppm (^4^ *J*_HP_ = 4 Hz) which collapses to a singlet upon ^31^P decoupling. The reaction of [**2**]^+^ with acetonitrile stands in sharp contrast to the reactivity of carbon‐based cumulenes, which undergo [2 + 2] cycloadditions with electron‐deficient substrates like tetrafluoroethylene (TFE), tetracyanoethylene (TCNE), bis(trifluoromethyl)acetylene, dimethyl acetylenedicarboxylate (DMAD) or C_60_.^[^ ^20^, ^21^, ^22^, ^23^ ^]^ [**2**]^+^ can be viewed as a polarized (P^δ+^─C^δ−^) electron‐deficient analogue of a carbon cumulene, which is reflected in its facile reactivity with an electron‐rich substrate like a nitrile.

**Figure 5:**
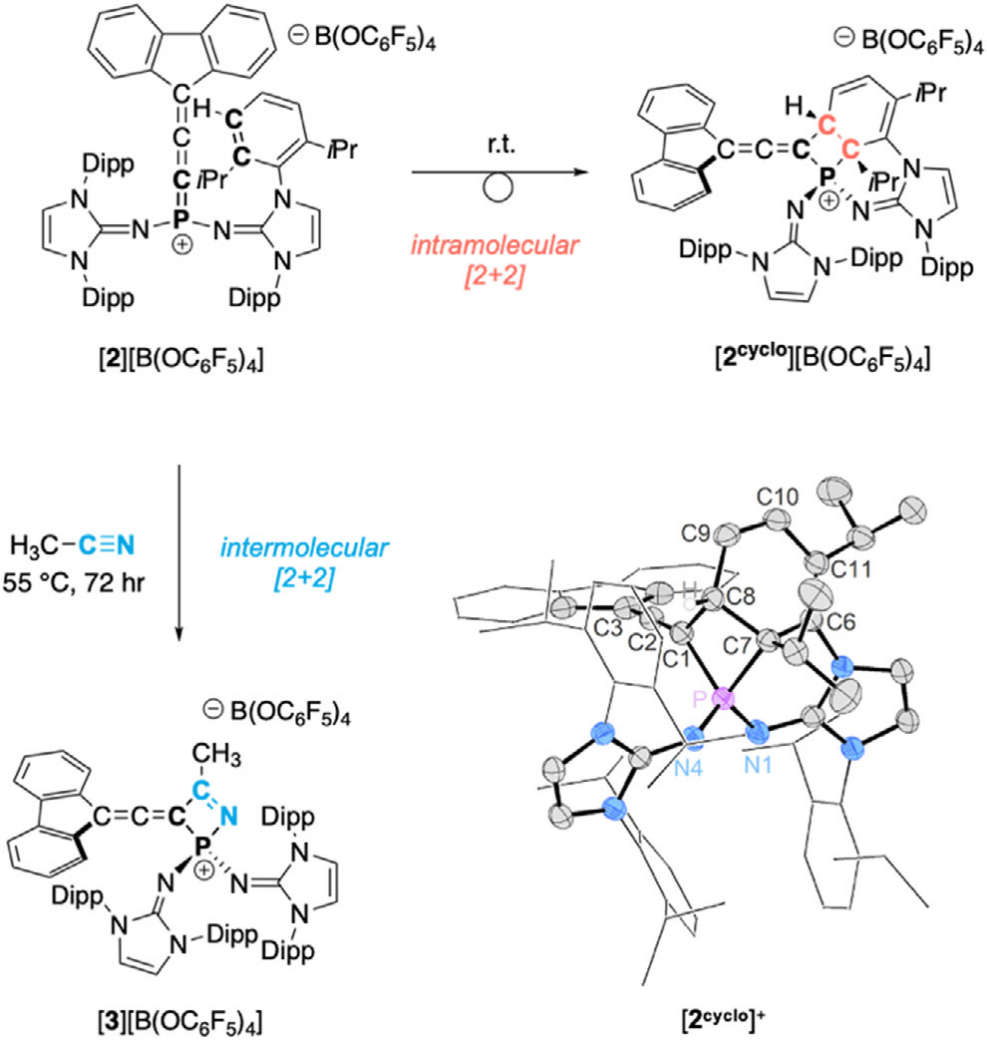
[2 + 2] cycloaddition of [**2**][B(OC_6_F_5_)_4_] to [**2^cyclo^**][B(OC_6_F_5_)_4_] in solution state (top). [2 + 2] cycloaddition of [**2**][B(OC_6_F_5_)_4_] with acetonitrile (left) to [**3**][B(OC_6_F_5_)_4_]. Molecular structure of [**2^cyclo^**][B(OC_6_F_5_)_4_] with the [B(OC_6_F_5_)_4_]^−^ anion excluded (bottom right). Thermal ellipsoids are shown at 50% probability. For clarity, hydrogen atoms are omitted, the Dipp groups and fluorenyl motif are shown in wireframe. Selected atomic distances [Å] and angles [°]: P─N1 1.604(2), P─N4 1.5573(19), P─C1 1.817(2), P─C7 1.834(2), C1─C8 1.555(3), C1─C2 1.295(3), C2─C3 1.318(3), C6─C7 1.517(3), C7─C8 1.563(3), C8─C9 1.497(3), C9─C10 1.317(3), C10─C11 1.469(3), C6─C11 1.347(3), N1─P─N4 115.74(10), N1─P─C1 118.06(11), N4─P─C1 114.05(11), N1─P─C7 112.38(10), N4─P─C7 112.64(11), C1─C2─C3 171.8(3).

The phosphonium cation [**2**]^+^ showed impressive stability in the solid state, with no indication of decomposition occurring at room temperature under an inert atmosphere. However, when dissolved at room temperature in polar non‐coordinating solvents, such as dichloromethane, trifluorotoluene, or 1,2‐difluorobenzene (1,2‐DFB), [**2**]^+^ slowly rearranges to a new compound quantitatively after five days. This new compound exhibits a singlet resonance (*δ* = +16.3 ppm) in the ^31^P NMR spectrum, and, most notably, this reaction proceeds to form the same product with the exclusion of light. The corresponding ^1^H NMR spectrum suggested a high degree of asymmetry in the product which initially precluded a structural assignment. Gratifyingly, colorless single crystals could be obtained by slow evaporation of a CH_2_Cl_2_ solution at room temperature. A SCXRD study revealed the compound to be the polycyclic phosphonium salt [**2^cyclo^**]**^+^**, resulting from a [2 + 2] cycloaddition of the P─C1 double bond with a C─C double bond of an adjacent 2,6‐diisopropylphenyl (Dipp) substituent. The dearomatized cyclohexa‐1,3‐diene ring of the tetracyclic [**2^cyclo^**]^+^ shows two double bonds (C6─C11 1.347(3) Å, C9─C10 1.317(3) Å) and four single bonds (C6─C7 1.517(3) Å, C7─C8 1.563(3) Å, C8─C9 1.497(3) Å, C10─C11 1.469(3) Å) (Figure 5). The P_1_C_3_ ring adopts a slightly puckered butterfly shape (interior angle sum of 352°), with the bond angle at the phosphorus center being the smallest (C1─P─C7 78°). [**2^cyclo^**]**^+^** has an allene fragment, with the substituents at C1 and C3 placed in orthogonal planes. Similar to [**3**]^+^, [**2^cyclo^**]**^+^** features ^13^C NMR resonances characteristic of a C3 allene system, the most notable being a very deshielded doublet at 194.2 ppm (^2^ *J*_CP_ = 10 Hz) corresponding to the sp hybridized central allene carbon.

Unlike [**2**]**^+^**, [**2^cyclo^**]**^+^** is surprisingly tolerant to atmospheric moisture and oxygen. Solution state samples of [**2^cyclo^**][B(OC_6_F_5_)_4_] showed no signs of decomposition with respect to the cation component after overnight exposure to ambient conditions. On the contrary, anion decomposition was observed after several hours, with ^19^F NMR spectra indicating the formation of several anionic borate species, as well as pentafluorophenol (see Supporting Information, Section 2.6). While not trivial, the synthesis of [**2^cyclo^**]**^+ ^** with a more robust weakly coordinating cation could be a promising avenue to accessing a water and air stable surrogate of [**2**]**^+^**.

DFT methods were once again used to gain mechanistic insight into the transformation of [**2**]**^+^** to [**2^cyclo^**]**^+^** using the truncated model compounds [**2**]**^+^_model_** and [**2^cyclo^**]**^+^_model_** having three of their Dipp substituents replaced with methyl substituents. Utilizing B3LYP−D3BJ/def2−TZVP level of theory and employing an implicit CH_2_Cl_2_ solvation, the reaction coordinates suggest the transformation occurs by a concerted [2 + 2] cycloaddition process *via* a single transition state (**TS1** Δ*G* ^‡^ = 131 kJ·mol^−1^). The isomers [**2**]^+^ **_model_** and [**2^cyclo^**]^+^ **_model_** have a minor energy difference, with the reaction process being slightly endergonic (Δ*G* = +26 kJ·mol^−1^), but equally exothermic (Δ*H* = −26 kJ·mol^−1^). We therefore wanted to explore whether an equilibrium between [**2**]^+^ and [**2^cyclo^**]^+^ could be established at elevated temperatures. Chemical corroboration of the thermal reversibility of [**2^cyclo^**]^+^ to [**2**]^+^ was demonstrated by gently heating a sample of [**2^cyclo^**]^+^ in 1,2‐DFB to 60 °C, in the dark, and trapping [2]^+^ with an excess of DMAP, yielding [**2•DMAP**][B(OC_6_F_5_)_4_]. Note that DMAP does not react with [**2^cyclo^**]^+^ at room temperature and required prolonged heating to achieve full conversion. The driving force for cycloreversion can be rationalized by the alleviation of ring strain and rearomatization of the Dipp substituent. This compensates for the energetic penalty associated with a change in geometry about the phosphorus center from tetrahedral [**2^cyclo^**]^+^ to trigonal planar [**2**]^+^ (see Supporting Information, Section 2.7).

The reversible activation of small molecule substrates by main‐group compounds has attracted much attention from the chemistry community as such behavior is a hallmark of transition‐metal catalyzed processes.^[^ ^58^, ^59^ ^]^ Bond activations involving arenes are of great relevance to organic chemists as they present opportunities to construct complex ring systems. For main group systems, inert arene activations are challenging and typically occur *via* a single atom insertion process, most often facilitated by an ambiphilic center such as a carbene^[^ ^17^, ^60^, ^61^, ^62^, ^63^, ^64^, ^65^, ^66^, ^67^ ^]^ or heavier analogues, such as phosphinidines,^[^ ^68^, ^69^, ^70^ ^]^ silylenes,^[^ ^71^, ^72^, ^73^, ^74^, ^75^, ^76^ ^]^ and aluminylenes.^[^ ^77^, ^78^ ^]^ Few arene activations occur across an unsaturated main group bond,^[^ ^79^, ^80^, ^81^, ^82^, ^83^ ^]^ and very rarely do these processes occur reversibly.^[^ ^84^, ^85^, ^86^ ^]^ Concerning trigonal planar phosphorus cations, only one previous study has demonstrated a reversible arene activation process. The Grützmacher group showed that the transient methylene phosphonium species [*^t^* Bu_2_P═C(Ph)(TMS)]^+^ and [*^t^* Bu_2_P═C(*o*‐tol)(TMS)]^+^ both undergo intramolecular electrocyclic ring closure with their proximal arene substituents to give benzannulated σ^4^ *λ* ^5^ phosphate salts.^[^ ^86^ ^]^ Stephan and co‐workers provided the first example of a reversible cycloaddition of a P─C multiple bond and an arene, a 1,4−cycloaddition which occurs intramolecularly between a Dipp substituent and a phosphalkene generated from a phosphepinium precursor.^[^ ^84^ ^]^ Despite sharing few similarities from a mechanistic perspective to the currently presented work, the process is reminiscent to recent contributions by the Bertrand group which details the isomerization of a super nucleophilic bis(imino)carbene to a polycyclic compound *via* a reversible [3 + 2] cycloaddition with a proximal Dipp substituent.^[^ ^87^ ^]^ Although unusual with arenes, such 1,3‐dipolar cycloaddition reactions, as well as [4 + 2] cycloaddition reactions with multiple bonds are common in organic synthesis to form five‐ and six‐membered rings, respectively. In contrast, we observe a reversible [2 + 2] cycloaddition between a phospha‐analogue [3]cumulene and an arene – an unprecedented transformation that contrasts sharply with our previously reported methylenephosphonium ions.^[^ ^17^ ^]^ These few examples highlight the unique behavior observed with our allenylidene phosphonium cation [**2**]^+^.

## Conclusion

Over a century since the synthesis of the first [3]cumulene by Brand,^[^ ^88^ ^]^ and three decades after Bertrand's pioneering work on trigonal planar phosphorus cations,^[^ ^8^ ^]^ here we demonstrate the preparation of a base‐free allenylidene phosphonium cation [2]^+^. Solid state structural data and quantum calculations indicate that [2]^+^ features a conjugated PCCC framework with a high degree of double bond character for the terminal P─C1 and C2─C3 bonds, as well as the central C1─C2 bond. Remarkably [2]^+^ undergoes an isomerization in solution to give [2^cyclo^]^+^, a rare example of a thermally reversible [2 + 2] cycloaddition between an unsaturated main‐group motif and an inert arene. Additionally, [2]^+^ can participate in a polarized intermolecular [2 + 2] cycloaddition as demonstrated by its reactivity with acetonitrile to yield [3]^+^. Further exploration of [2]^+^ in bond activation reactions are currently under investigation and will be reported in due course. Overall, our work illustrates the feasibility of replacing sp^2^ carbon atoms with three‐coordinate P(V) centers in π conjugated molecules and serves to further strengthen the carbon‐phosphorus diagonal relationship. While the structure and bonding analyses of [2]^+ ^provides fascinating comparisons to carbon [3]cumulenes, its ability to promote reversible bond activation supports the notion that unsaturated main group systems like trigonal planar phosphonium cations can facilitate reactivity patterns which are typically observed for transition metals complexes.

## Supporting Information

The authors have cited additional references within the Supporting Information.^[^ ^41^, ^42^, ^43^, ^44^, ^45^, ^46^, ^47^, ^48^, ^49^, ^50^, ^51^, ^52^, ^53^, ^54^, ^55^, ^56^, ^57^, ^58^, ^59^, ^60^, ^61^, ^62^ ^]^ Crystallographic data have been submitted to the CCDC: 2371601–2371604.

## Author Contributions

Conceptualization of the project was by F.D., C.B.C., P.L., and L.C.T. The project was supervised and directed by F.D. and C.B.C. Synthetic experimentation was performed by L.C.T. and M.B.R. Computations performed by A.B. SCXRD studies performed by M.S., J.L., and K.W. The manuscript was written by L.C.T., A.B. C.B.C, and F.D. and edited by L.C.T., P.L., A.B., F.D., and C.B.C. All authors have given approval for the final version of the manuscript.

## Conflict of Interests

The authors declare no conflict of interest.

## Supporting information



Supporting information

Supporting information

Supporting information

## Data Availability

The data that support the findings of this study are available in the supplementary material of this article.
