# Supplementary material for: Allenylidene Phosphonium Ion: An Isoelectronic Phosphorus Analogue of [3]Cumulene
Source: Angew Chem Int Ed Engl. 2025 Apr 10;64(21):e202502201. doi: 10.1002/anie.202502201 (PMC12087857; doi:10.1002/anie.202502201)
Supplement: Supplementary file 1 — Supporting information [file ANIE-64-e202502201-s001.pdf]

Supplementary Information for

Allenylidene Phosphonium Ion: An Isoelectronic Phosphorus Analogue of [3]Cumulene

Lucas C. Torres,[a,c] Pawel Löwe,[b]† Avik Bhattacharjee,[a]† Maike B. Röthel,[c] Michael Seidl,[c] Jesse LeBlanc,[a] Klaus Wurst,[c] Fabian Dielmann,[c]* Christopher B. Caputo.[a]*

a. Department of Chemistry, York University, 4700 Keele St, Toronto, ON, M3J 1P3 (Canada) E-mail: Caputo@yorku.ca

b. Institute of Inorganic and Analytical Chemistry, Westfälische Wilhelms-Universität Münster, 48149, Correnstrasse 30, 48149 Münster (Germany)

c. Institute of General, Inorganic and Theoretical Chemistry, Leopold-Franzens-Universität Innsbruck, Innrain 80-82, 6020 Innsbruck (Austria) E-mail: Fabian.Dielmann@uibk.ac.at

Table of Contents

1 General Experimental Procedures S2

2 Synthetic Procedures and Spectroscopic Characterization S3-69

3 Crystallographic Studies S70-74

4 Computational Studies S75-81

5 References S81-83

1.0 General Experimental Procedures

General Remarks: All manipulations were performed under an inert atmosphere of either dry argon or nitrogen, employing standard Schlenk and glovebox techniques. Dry and oxygen-free solvents were utilized. All glassware was oven-dried at 150 °C. ^{1}H, ^{11}B, ^{13}C, ^{19}F, and ^{31}P nuclear magnetic resonance (NMR) spectra were recorded on Bruker Neo 700 MHz, Bruker ARX 400 MHz, Bruker AVANCE (IV) Neo 400 MHz, Bruker ARX 300 MHz spectrometers. Chemical shifts are given in parts per million (ppm) relative to $SiMe_4$ (^{1}H, ^{13}C), BF_3•Et_2O (^{11}B, ^{19}F), and 85% H_3PO_4 (^{31}P) and they were referenced to the residual solvent signals (CD_2Cl_2: ^{1}H d_H =5.32, ^{13}C d_C = 54.00; C_6D_6: ^{1}H d_H = 7.16, ^{13}C d_C = 128.06; CD_3CN: d_H =1.94, ^{13}C d_C = 118.26) or internally by the instrument after locking and shimming to the deuterated solvent (^{11}B, ^{19}F, ^{31}P). Coupling constants are reported in Hertz (Hz) and NMR multiplicities are abbreviated as follows: s = singlet, d = doublet, t = triplet, q = quartet, p = pentet, sext = sextet, sept = septet, m = multiplet, br = broad signal. Mass spectrometry was recorded using an QExactive Orbitrap (Thermo Scientific). FT-IR absorption measurements were recorded with a Bruker Alpha FT-IR (Platinum ATR unit) spectrometer, within an argon atmosphere glovebox. UV-Vis absorption measurements were recorded with a Cary 5000 UV-Vis-NIR Spectrophotometer from Agilent Technologies. Recordings were obtained at 25 °C and taken with the instrument operating in dual beam mode and referenced to dichloromethane. All absorption experiments were conducted in quartz cuvettes (1 cm x 1 cm) equipped with a J-Young style Teflon tap. Cyclic voltammetry experiments were performed under an argon atmosphere using a Metrohm Autolab potentiostat. A three-electrode cell was utilized with a glassy carbon working electrode, platinum wire counter electrode, and a Ag/AgCl pseudo-reference electrode. Formal redox potentials were referenced to the Ag/AgCl redox couple. 0.1 M $^{n}Bu_4PF_6$ was used as the supporting electrolyte. 1,2-difluorobenzene was used as the solvent, and the scan rate was set to 100 mV/s. The voltammogram was recorded using the NOVA software package.

Reagents and Handling: All compounds were purchased from commercial sources (Sigma Aldrich, Alfa Aesar, Tokyo Chemical Industry, Oakwood chemicals) and used as received, if not stated differently. $[(R^2)_2P]Cl$ (R^2 =1,3-diisopropylphenylimidazolin-2-ylidenamino, NIDipp)[41] and $B(OC_6F_5)_3$[42] were synthesized according to literature procedure.

2. Synthetic Procedures and Spectroscopic Characterization

2.1 Preparation of bis(imino)alkynylphosphine (1)

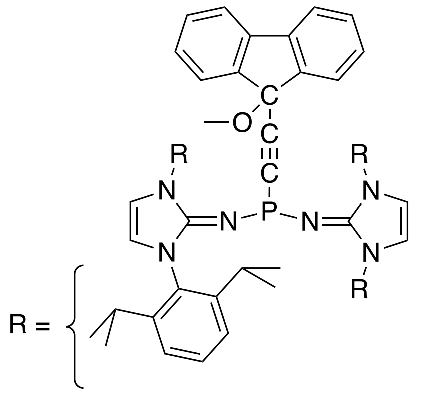

9-Ethynyl-9-methoxy-9H-fluorene (50 mg, 0.227 mmol, 1.00 equiv) was dissolved in THF (~2 mL) and cooled to -78°C. n-BuLi (142 mL, 1.6 M in hexanes, 0.227 mmol, 1.00 equiv) was then added in a single portion. The reaction mixture was kept at -78°C and shaken periodically over the course of 30 minutes. The mixture was then added dropwise to a precooled (-78°C) solution of [(NIDipp)$_2$P][Cl] (198 mg, 0.227 mmol, 1.00 eq) in THF (~2 mL) and subsequently allowed to warm to room temperature to stir for 16 hours. All volatiles were removed *in vacuo*. The crude product was extracted with pentane (30 mL) and passed through a glass filter. The solvent was then removed in *in vacuo*, to provide the title compound as a yellow powder. Compound **1** is soluble in THF, diethyl ether, benzene, and toluene, and sparingly soluble in *n*-pentane and *n*-hexane. It decomposes slowly in $CHCl_3$, CH_2Cl_2, and CH_3CN.

Yield: 140 mg (0.133 mmol, 58%).

^{1}H NMR (C_6D_6, 400 MHz, 298 K) δ (ppm) = 7.71 (d, $^3J_{HH}$ = 7.0 Hz, 2H, CH fluorenyl), 7.42 (d, d, $^3J_{HH}$ = 7.0 Hz, 2H, CH fluorenyl), 7.27 – 7.20 (m, 4H, CH fluorenyl), 7.19 (s, 2H, CH Dipp: *para*), 7.17 (s, 2H, CH Dipp: *para*), 7.14 – 7.09 (m, 4H, CH Dipp: *meta*), 6.94 – 6.89 (m, 4H, CH Dipp: *meta*), 5.79 (s, 4H, N-C<u>H</u>=C<u>H</u>-N), 3.20 (sept, $^3J_{HH}$ = 7.1 Hz, 4H, C<u>H</u>(CH_3)$_2$), 3.03-2.93 (m, 7H, C<u>H</u>(CH_3)$_2$ and C-OC<u>H</u>$_3$), 1.26 (d, $^3J_{HH}$ = 6.8 Hz, 12H, CH(C<u>H</u>$_3$)$_2$), 1.18 (d, $^3J_{HH}$ = 6.9 Hz, 12H, CH(C<u>H</u>$_3$)$_2$), 1.11 (d, $^3J_{HH}$ = 6.8 Hz, 12H, CH(C<u>H</u>$_3$)$_2$), 1.01 (d, $^3J_{HH}$ = 6.9 Hz, 12H, CH(C<u>H</u>$_3$)$_2$).

^{13}C {^{1}H} NMR (C_6D_6, 101 MHz, 298 K) δ (ppm) = 148.3 (C_q Dipp: *ortho*), 148.3 (C_q Dipp: *ortho*), 147.1 (C_q Dipp: *ortho*), 145.9 (C_q fluorenyl), 143.8 (d, $^2J_{CP}$ = 29.1 Hz, C_q N-C-N), 141.1 (C_q fluorenyl), 135.2 (C_q Dipp: *ipso*), 129.3 (CH fluorenyl), 128.9 (CH Dipp: *para*), 128 (overlapped with C_6D_6, CH fluorenyl), 126.9 (CH fluorenyl), 124.0 (CH Dipp: *meta*), 123.8 (CH Dipp: *meta*), 119.7 (CH fluorenyl), 115.1 (N-<u>C</u>H=<u>C</u>H-N), 94.9 (d , $^1J_{CP}$ = 65.4 Hz, P-<u>C</u>≡C-), 92 (d , $^2J_{CP}$ = 7.6 Hz, P-C≡<u>C</u>-), 81.2 (d, $^3J_{CP}$ = 2.4 Hz, <u>C</u>-OCH$_3$) 50.9 (C-O<u>C</u>H$_3$), 29.2 (<u>C</u>H(CH_3)$_2$), 28.9 (<u>C</u>H(CH_3)$_2$), 28.9 (<u>C</u>H(CH_3)$_2$), 24.9 (CH(<u>C</u>H$_3$)$_2$), 24.0 (CH(<u>C</u>H$_3$)$_2$), 23.9 (CH(<u>C</u>H$_3$)$_2$), 23.8 (CH(<u>C</u>H$_3$)$_2$), 23.8 (CH(<u>C</u>H$_3$)$_2$).

^{31}P NMR (C_6D_6, 162 MHz, 298 K) δ (ppm) = 51.9.

HR-ESI-MS: Calculated for [$C_{70}H_{84}N_6OP$]$^+$ ([**1**+H]$^+$) m/z = 1054.6444, found: m/z = 1055.6415.

Single crystal X-ray diffraction analysis: Yellow single crystals suitable for X-ray diffraction were obtained by storing a concentrated n-hexane solution of the product at -40 °C.

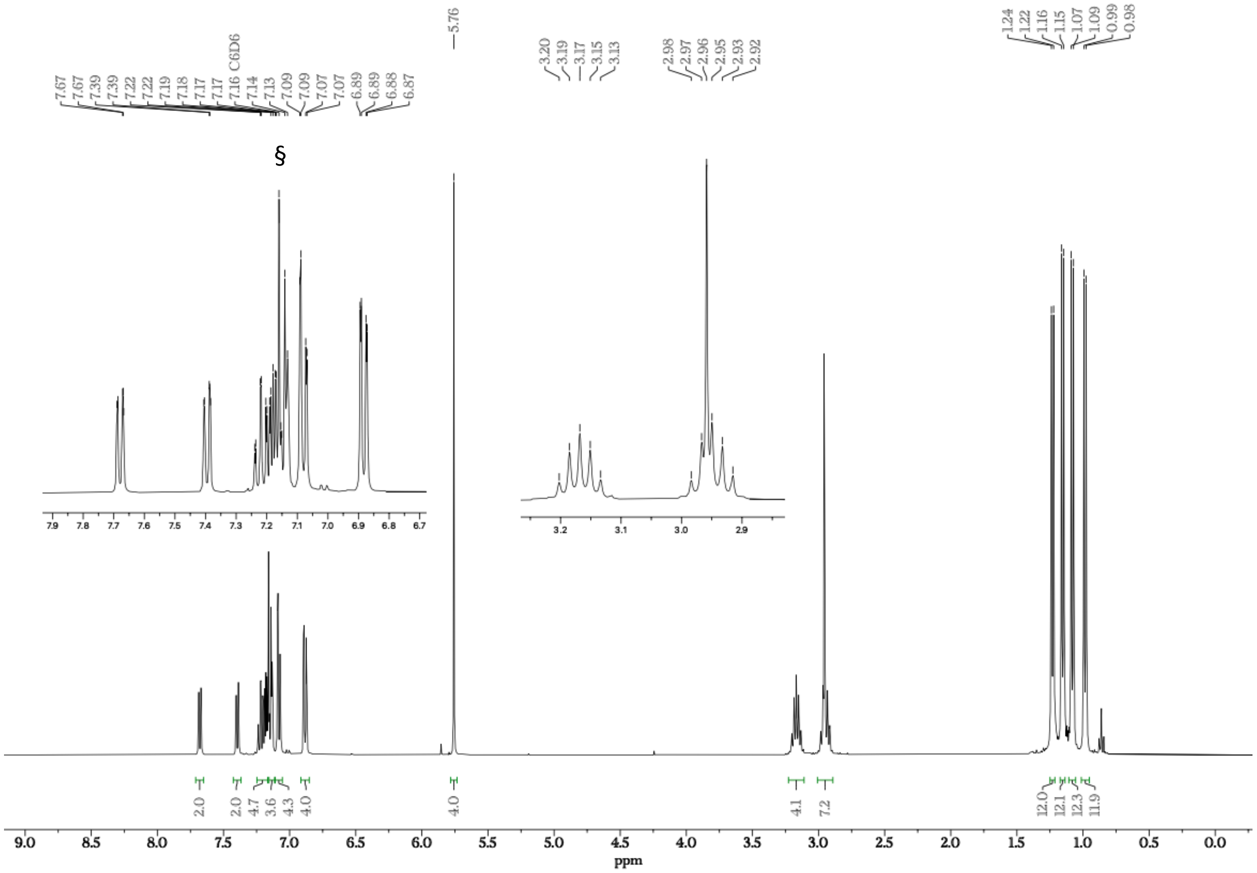

Figure S1: ^{1}H NMR (400 MHz, 298 K) spectrum of **1** in C_6D_6. § marks the solvent signal.

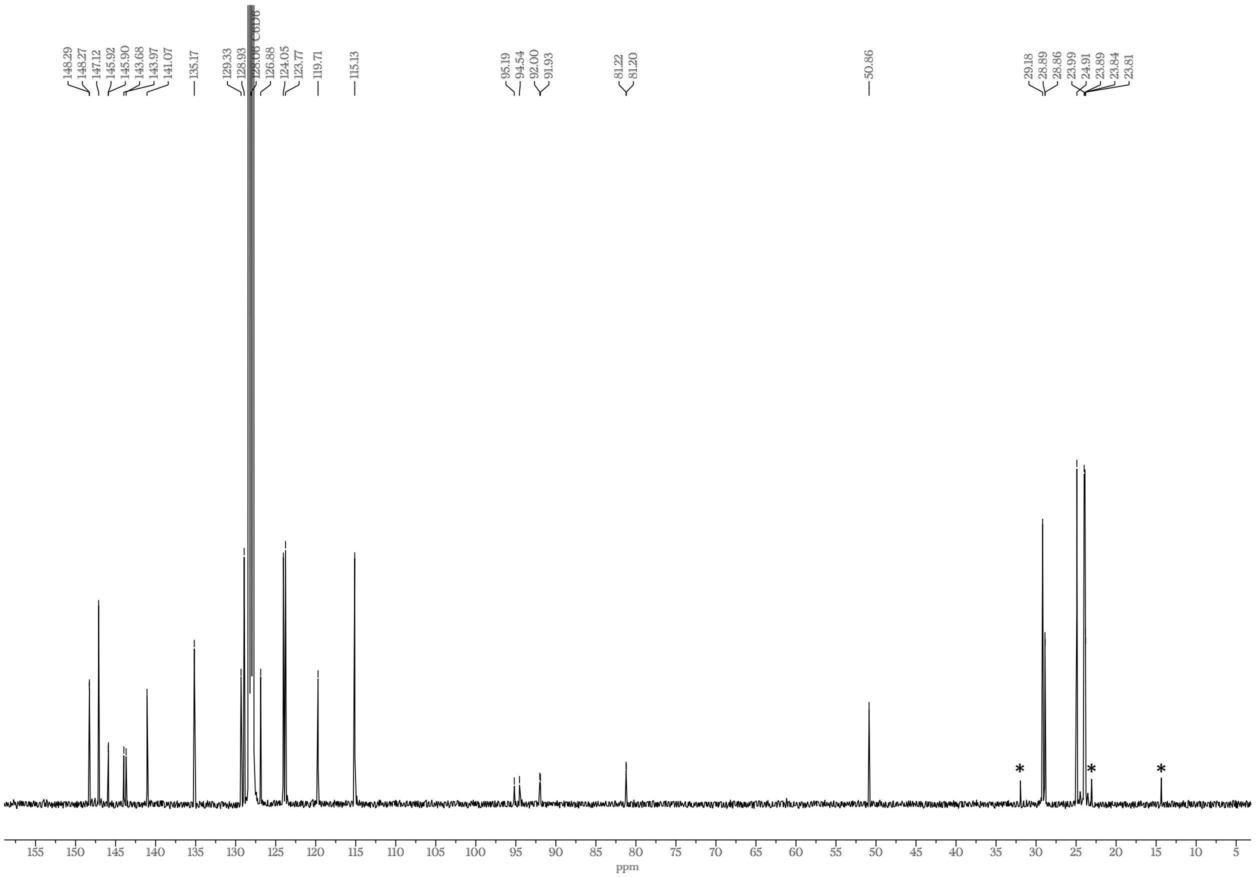

Figure S2: $^{13}C\{^{1}H\}$ NMR (101 MHz, 298 K) spectrum of **1** in C_6D_6,. The asterisk denotes residual pentane.

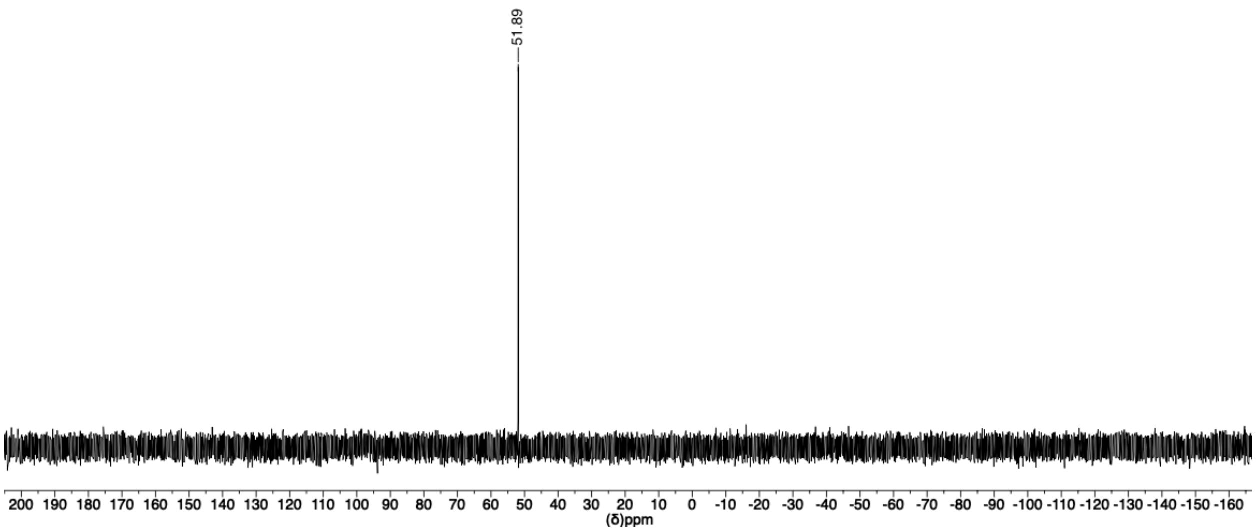

Figure S3: ^{31}P NMR (162 MHz, 298 K) spectrum of **1** in C_6D_6.

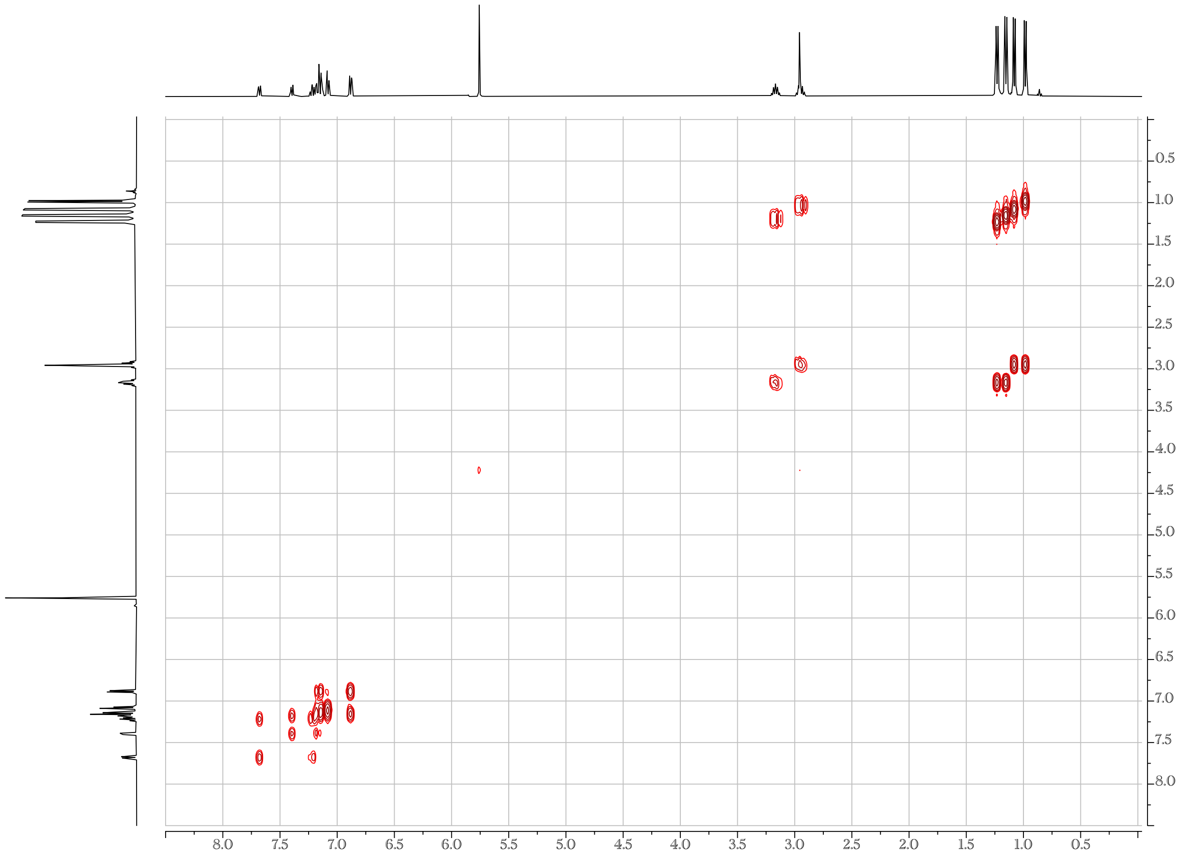

Figure S4: $^{1}H/^{1}H$ COSY NMR spectrum of **1** in C_6D_6.

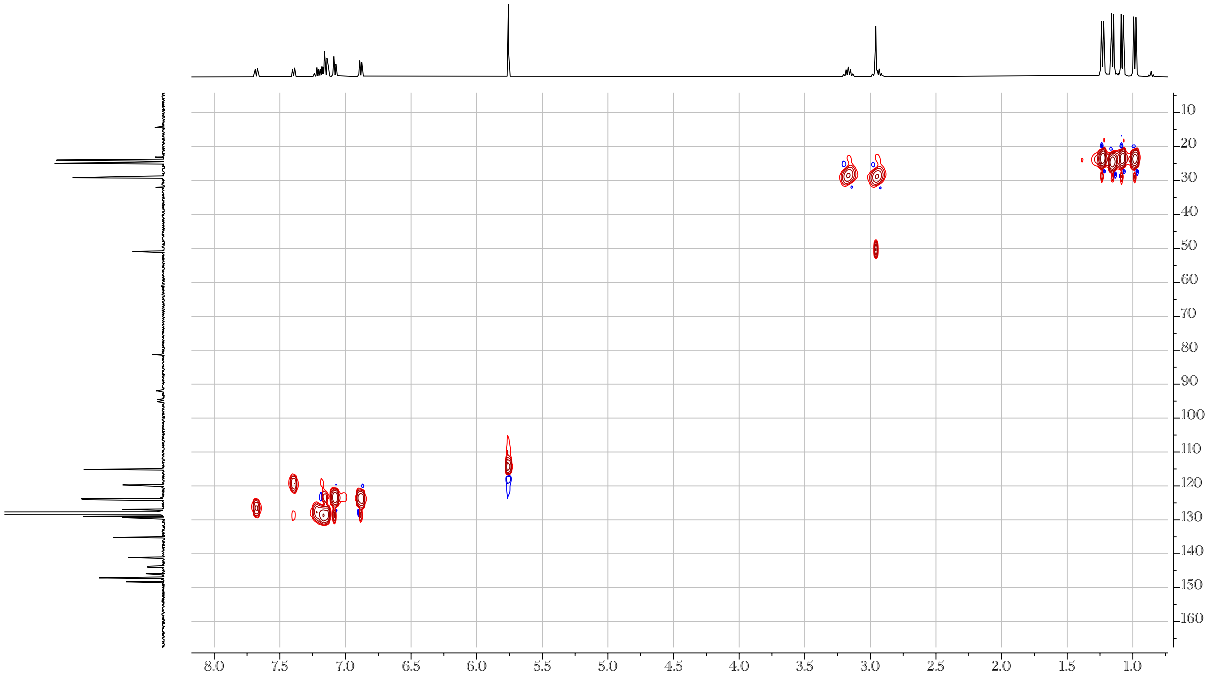

Figure S5: $^{1}H/^{13}C\{^{1}H\}$ HSQC NMR spectrum of **1** in C_6D_6.

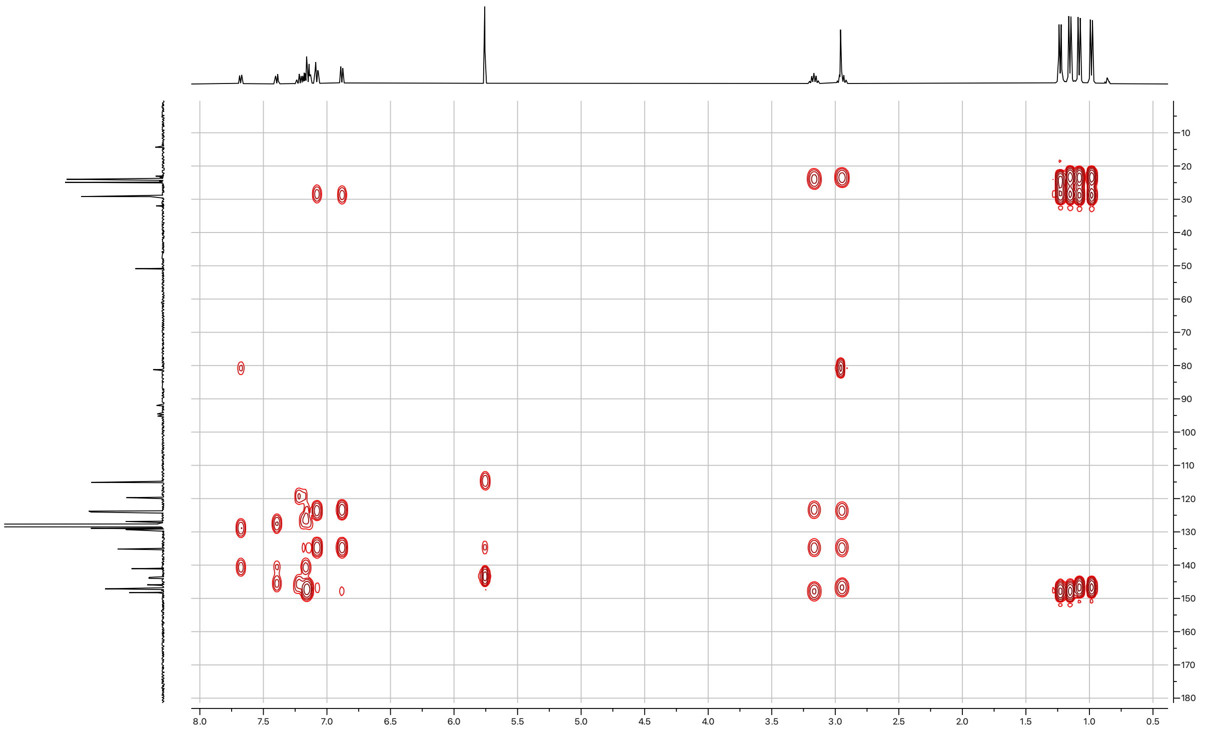

Figure S6: $^{1}H/^{13}C\{^{1}H\}$ HMBC NMR spectrum of **1** in C_6D_6.

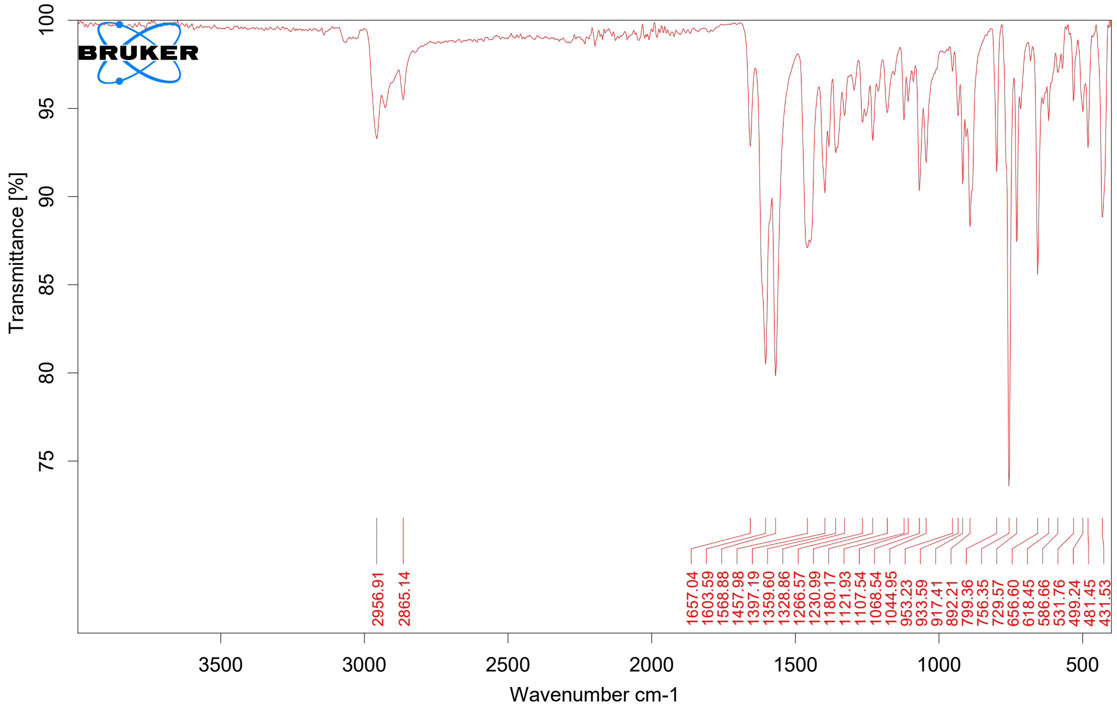

Figure S7: Solid state IR spectrum of **1**.

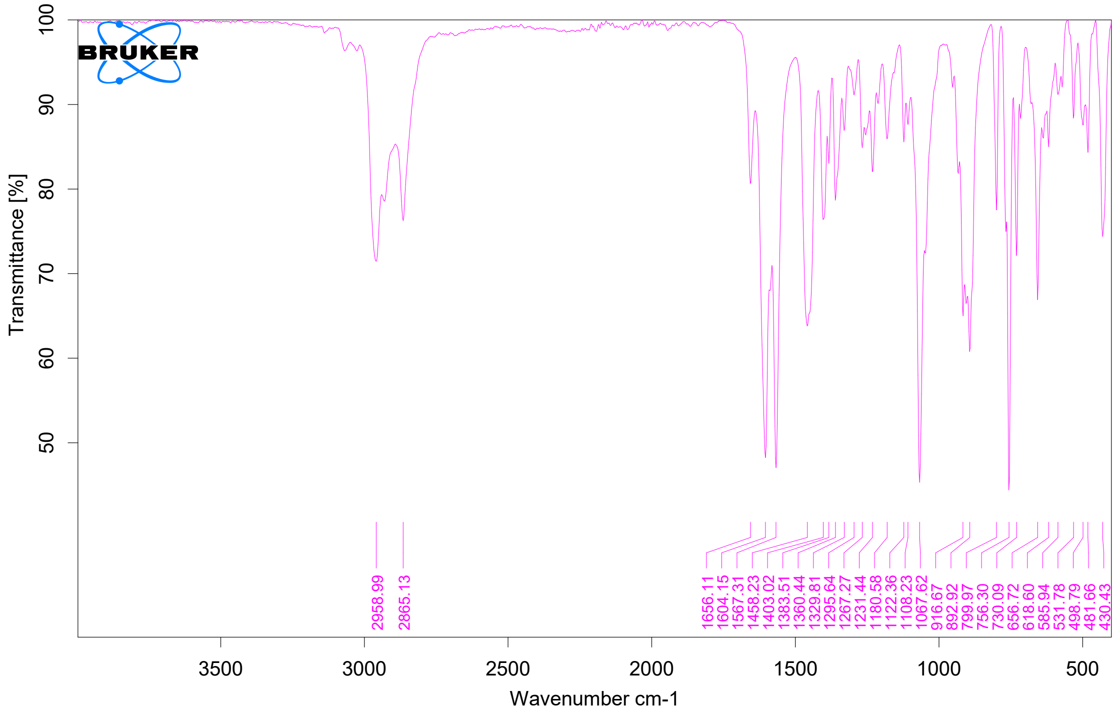

Figure S8: Solution state (CH_2Cl_2) IR spectrum of **1**.

2.2 Preparation of allenylidene phosphonium salt [2][B(OC_6F_5)$_4$]

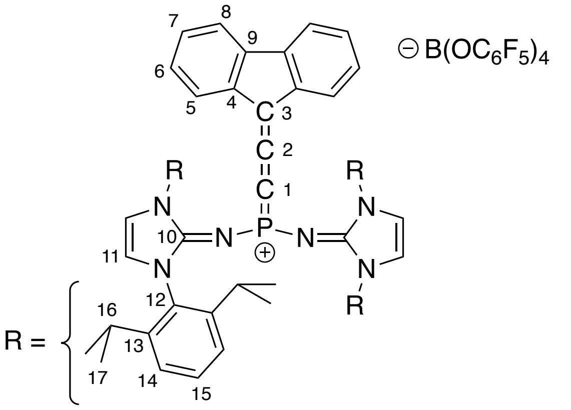

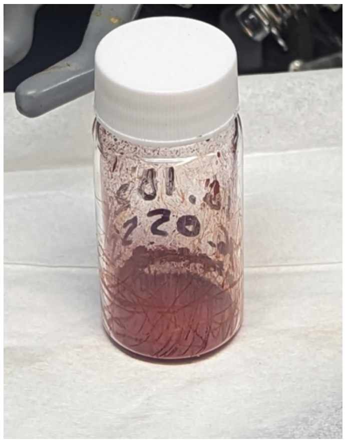

Solid state sample of [**2**][B(OC_6F_5)$_4$]

1 (40 mg, 38 mmol, 1.00 equiv) and B(OC_6F_5)$_3$ (30 mg, 54 mmol, 1.42 equiv) were each dissolved in *n*-pentane (5 mL). The B(OC_6F_5)$_3$ solution was added dropwise to the solution of **1** at room temperature while vigorously stirring. The combined solution gradually became turbid with precipitation of a red solid. The reaction was allowed to stir for 30 minutes, and the red solid was left to settle to the bottom of the reaction container. The supernatant was decanted, and the solid residue was washed with a generous amount of pentane (3 x 10 mL) until the resulting supernatant appeared colourless. Afterwards, all volatiles were removed *in vacuo*. The product was isolated as a red solid.

Yield: 47 mg (27 mmol, 71 %).

Note: For ease of reproducing the synthesis, the reaction was done using 1.42 equiv of borate relative to phosphine, since this provides conveniently measurable masses (40 mg of phosphine and 30 mg of borate).

For NMR analysis, a solid sample of [**2**][B(OC_6F_5)$_4$] and CD_2Cl_2 were stored at -78 °C for several minutes before preparing the NMR sample. Once the solution was made, the NMR tube was stored in a dry ice/acetonitrile bath (-40 °C), until it was placed in the spectrometer, which was precooled to the listed acquisition temperatures. At approximately room temperature, [**2**]$^+$ begins to slowly convert to [**2**$^{\mathbf{cyclo}}$]$^+$.

^{1}H NMR (CD_2Cl_2, 700 MHz, 258 K) δ (ppm) = 7.83 (d, $^3J_{HH}$ = 7.7 Hz, 2H, CH fluorenyl), 7.46 (t, $^3J_{HH}$ = 7.8 Hz, 4H, CH Dipp: *para*), 7.30 (t, $^3J_{HH}$ = 7.5 Hz, 2H, CH fluorenyl), 7.26 (t, $^3J_{HH}$ = 7.5 Hz, 2H, CH fluorenyl), 7.11 (d, $^3J_{HH}$ = 7.8 Hz, 8H, CH Dipp: *meta*), 7.08 (d, $^3J_{HH}$ = 7.7 Hz, 2H, CH fluorenyl), 7.02 (s, 4H, N-C$\underline{H}$=C$\underline{H}$-N), 2.41 (m, 8H, C$\underline{H}$(CH_3)$_2$), 1.06 (d, $^3J_{HH}$ = 6.9, 24 H, CH(C$\underline{H}_3$)$_2$), 0.81 (d, $^3J_{HH}$ = 6.9, 24 H, CH(C$\underline{H}_3$)$_2$).

^{11}B NMR (CD_2Cl_2, 96 MHz, 253 K) δ (ppm) = 1.6.

$^{13}C\{^1H\}$ NMR (CD_2Cl_2, 176 MHz, 258 K) δ (ppm) = 161.9 (d, $^2J_{CP}$ = 34 Hz, P=C=<u>C</u>=C), 145.6 (Dipp: *ortho*), 143.5 (d, $^2J_{CP}$ = 13 Hz, N-C-N), 142.1 (dm, $^1J_{CF}$ = 246 Hz, CF $B(OC_6F_5)_4$: *ortho*), 139.1 (d, $^4J_{CP}$ = 10 Hz, C_q fluorenyl), 137.7 (dm, $^1J_{CF}$ = 244 Hz, CF $B(OC_6F_5)_4$: *para*), 136.0 (d, $^5J_{CP}$ = 3 Hz, C_q fluorenyl), 134.9 (dm, $^1J_{CF}$ = 242 Hz, CF $B(OC_6F_5)_4$: *meta*), 133.0 (m, CF $B(OC_6F_5)_4$: *ipso*), 131.4 (Dipp: CH *para*), 129.0 (Dipp: *ipso*), 126.5 (CH fluorenyl), 126.3 (d, $^6J_{CP}$ = 3 Hz, CH fluorenyl), 124.9 (Dipp: CH *meta*), 121.7 (d, $^5J_{CP}$ = 4 Hz, CH fluorenyl), 120.8 (CH fluorenyl), 119.3 (N-CH=CH-N), 99.1 (d, $^1J_{PC}$ = 263 Hz, P=<u>C</u>=C=C), 94.6 (d, $^3J_{PC}$ = 31 Hz, P=C=C=<u>C</u>), 29.1 (<u>C</u>H(CH_3)$_2$), 25.2 (CH(<u>C</u>H_3)$_2$), 22.8 (CH(<u>C</u>H_3)$_2$).

^{19}F NMR (CD_2Cl_2, 282 MHz, 253 K) δ (ppm) = -156.9 (d, $^3J_{FF}$ = 20.4 Hz, 8F, CF $B(OC_6F_5)_4$: *ortho*), -167.9 (m, 8F, CF $B(OC_6F_5)_4$: *meta*), -171.2 (t, $^3J_{FF}$ = 20.4 Hz, 4F, CF $B(OC_6F_5)_4$: *para*).

^{31}P NMR (CD_2Cl_2, 121 MHz, 253 K) δ (ppm) = 42.9.

Single crystal X-ray diffraction analysis: Single crystals suitable for X-ray diffraction were obtained by storing a solution (CH_2Cl_2 /pentane mixture) of the product at -40 °C for several weeks.

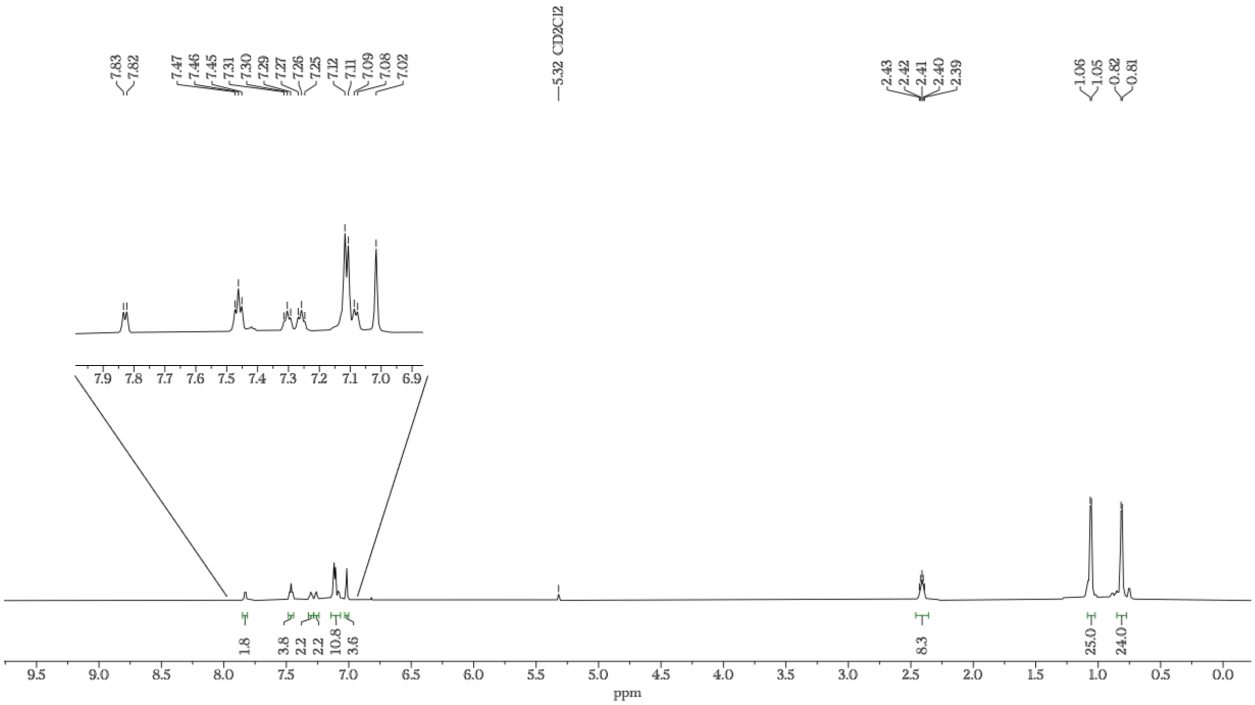

Figure S9: ^{1}H NMR (700 MHz, 258 K) spectrum of [**2**][$B(OC_6F_5)_4$] in CD_2Cl_2.

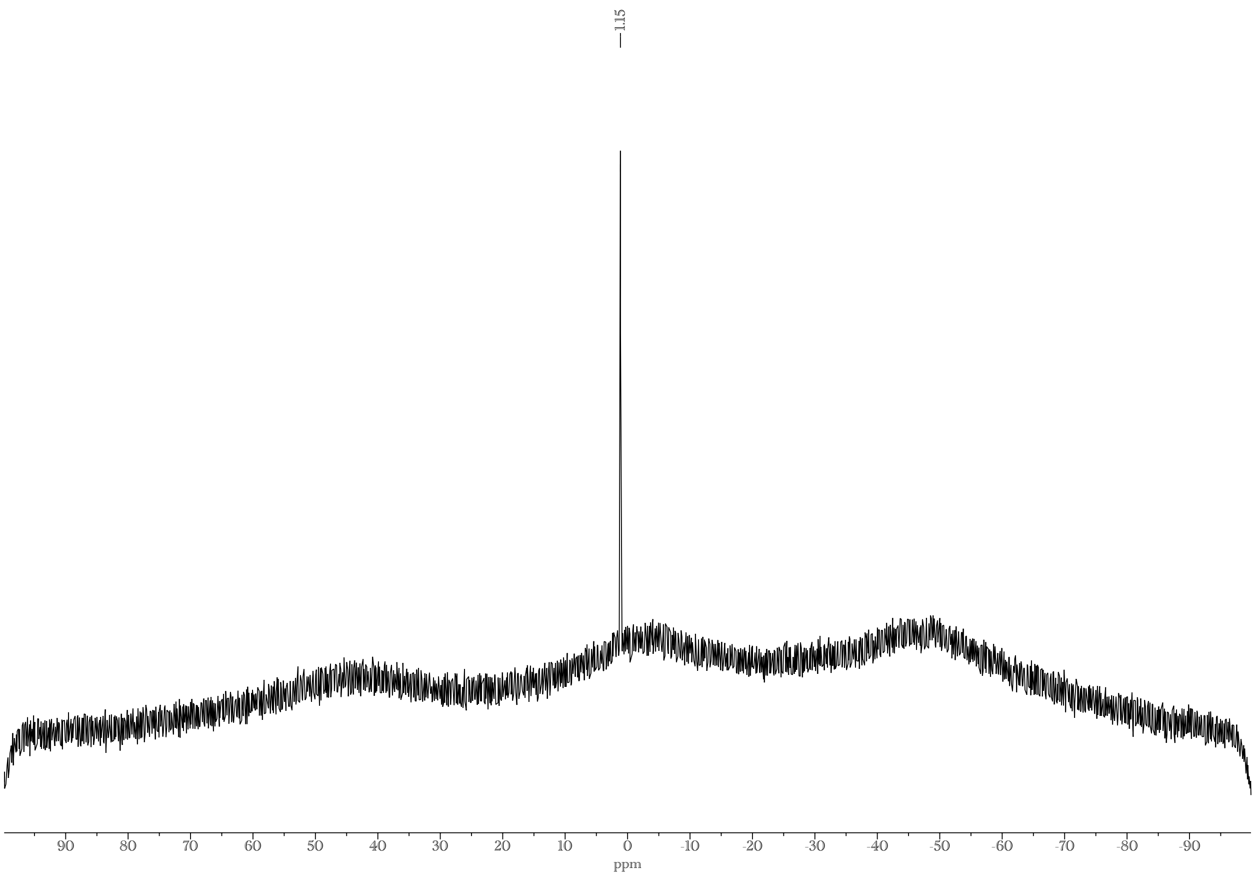

Figure S10: ^{11}B NMR (96 MHz, 253 K) spectrum of [**2**][$B(OC_6F_5)_4$] in CD_2Cl_2.

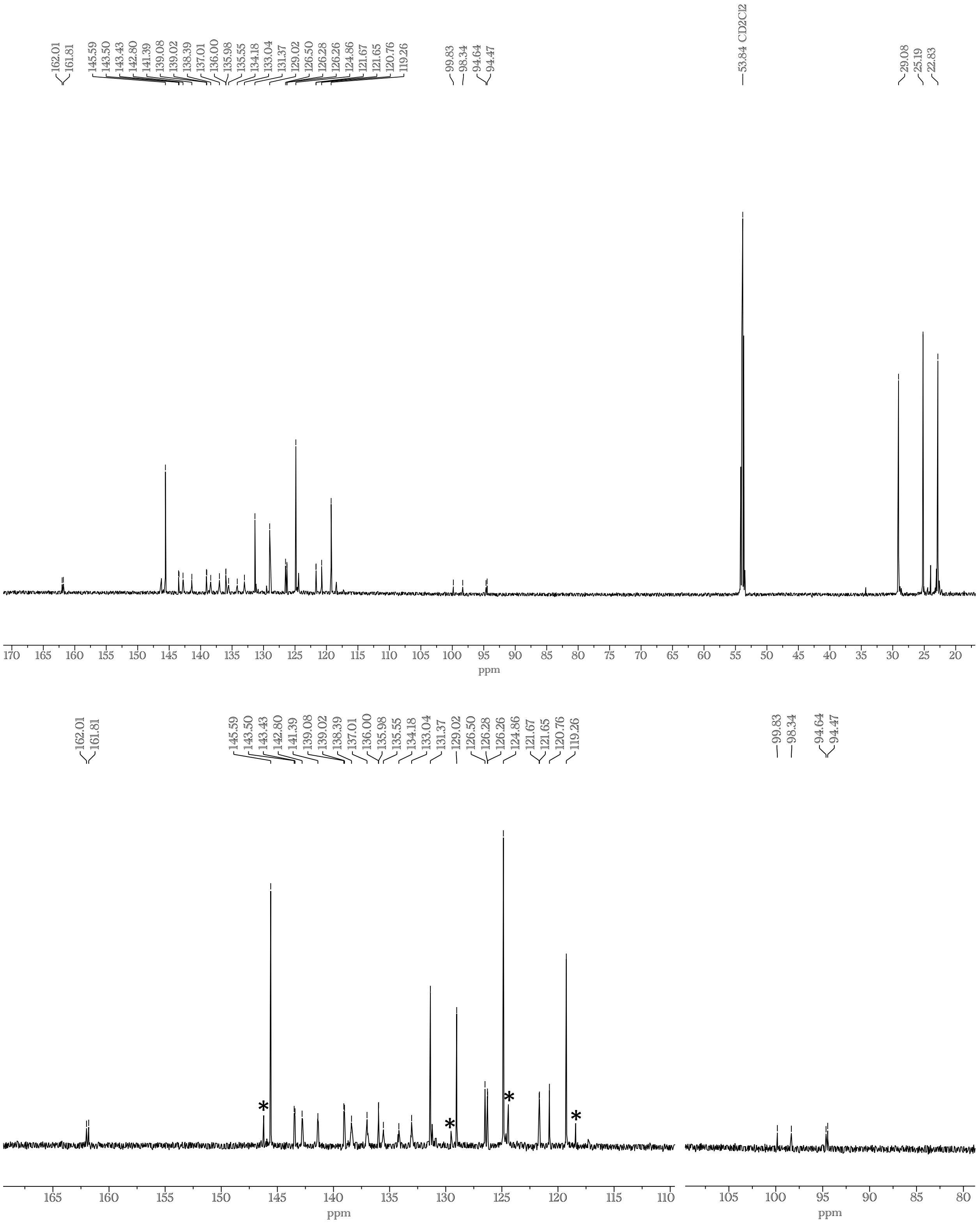

Figure S11: $^{13}C\ \{^{1}H\}$ NMR (176 MHz, 258 K) spectrum of [**2**][$B(OC_6F_5)_4$] in CD_2Cl_2. * Reflect trace decomposition impurities.

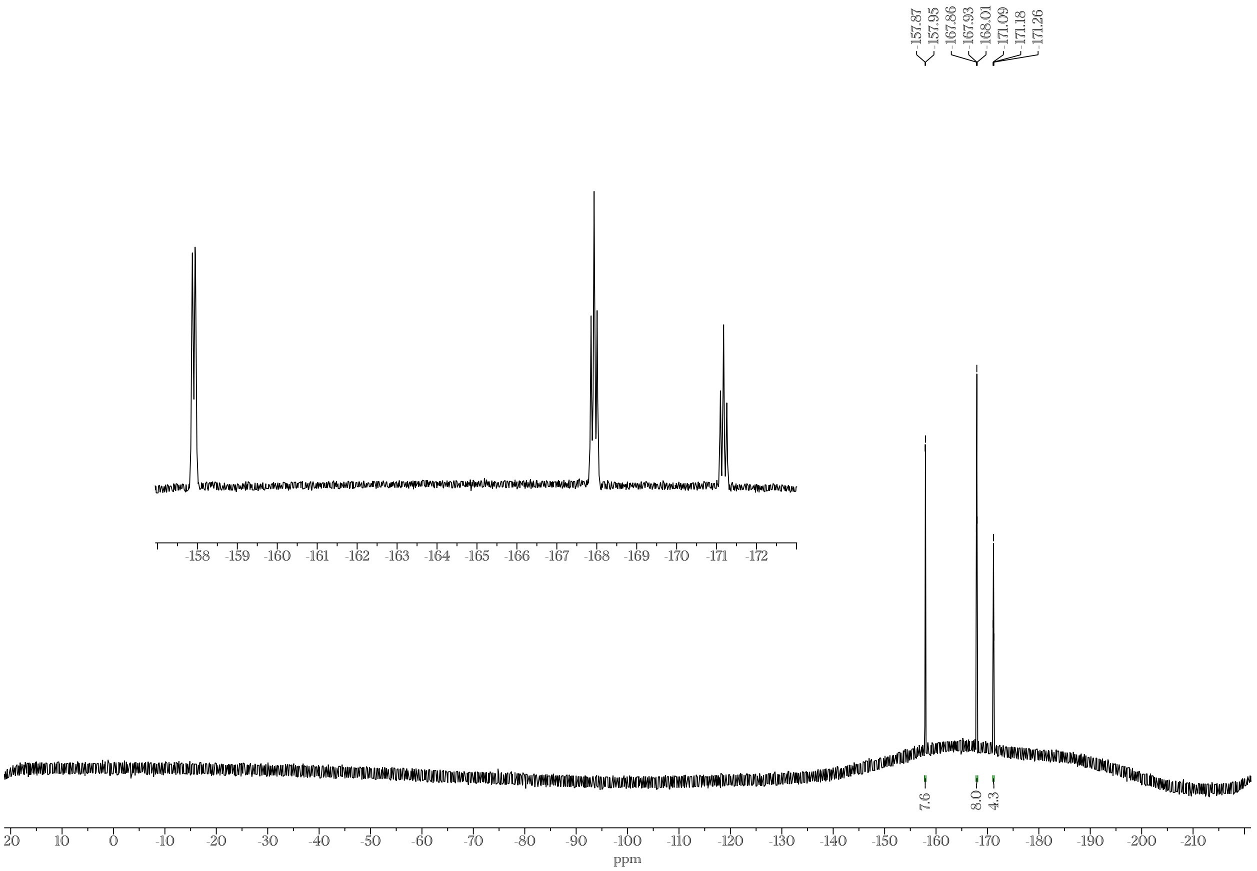

Figure S12: ^{19}F NMR (282 MHz, 253 K) spectrum of [**2**][$B(OC_6F_5)_4$] in CD_2Cl_2.

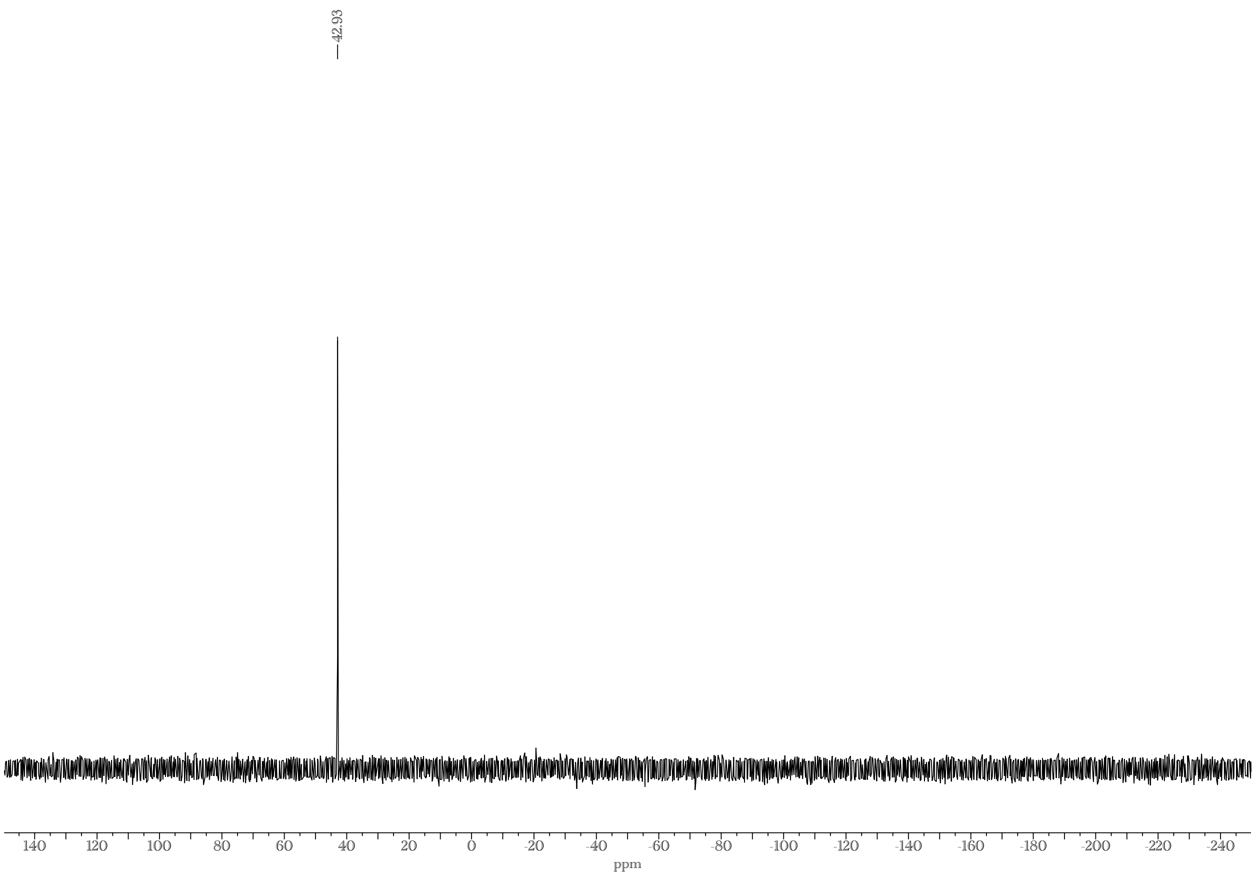

Figure S13: $^{31}P\{^{1}H\}$NMR (121 MHz, 253 K) spectrum of [**2**][$B(OC_6F_5)_4$] in CD_2Cl_2.

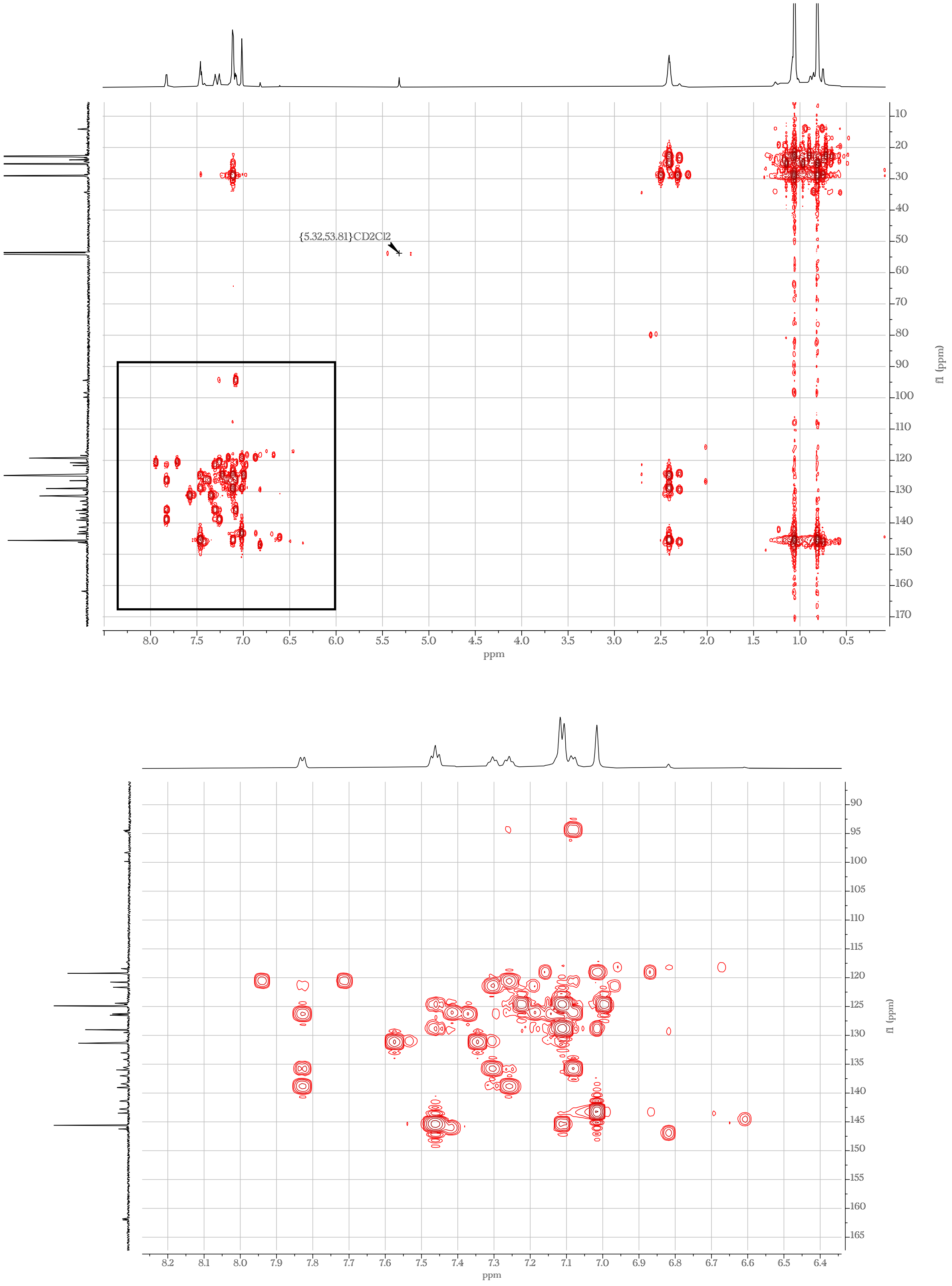

Figure S14: $^{1}H/^{13}C\{^{1}H\}$ HMBC NMR spectrum of [**2**][B(OC$_6$F$_5$)$_4$] in CD_2Cl_2.

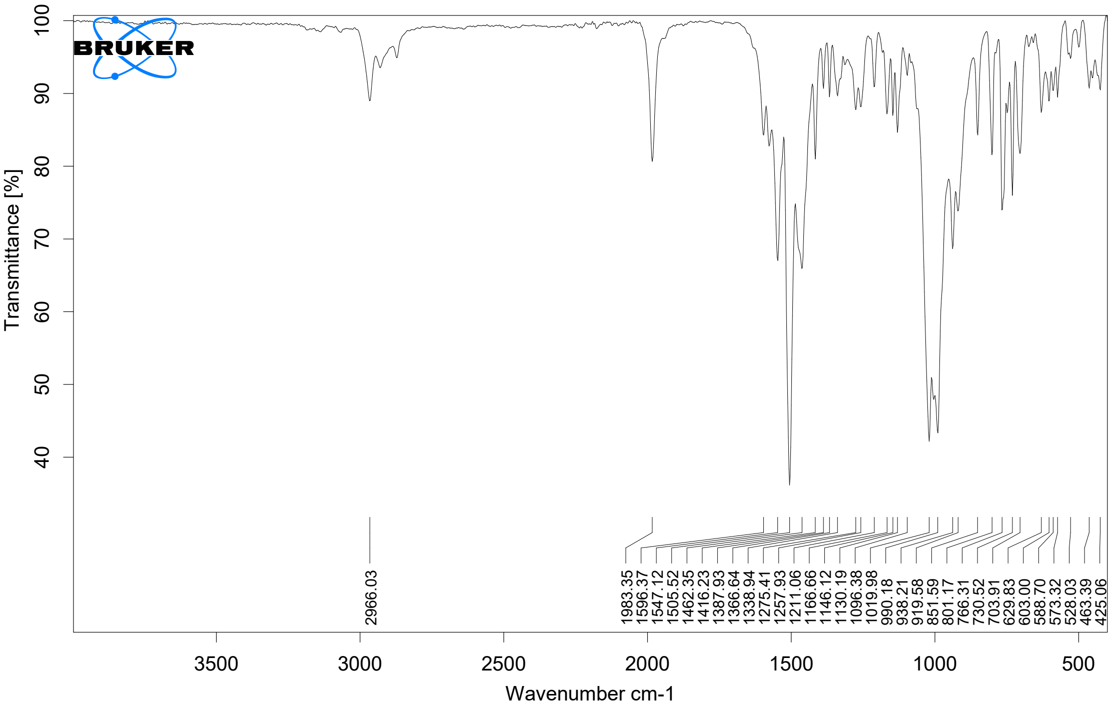

Figure S15: Solid state IR spectrum of [**2**][B(OC_6F_5)$_4$].

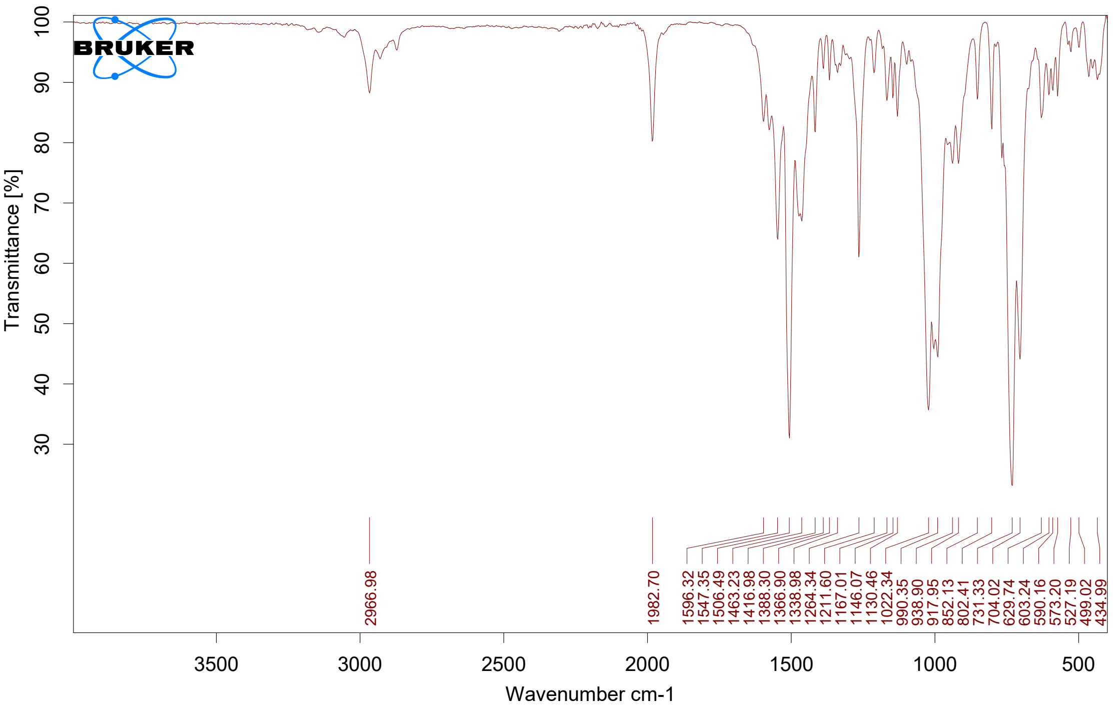

Figure S16: Solution state (CH_2Cl_2) IR spectrum of [**2**][B(OC_6F_5)$_4$].

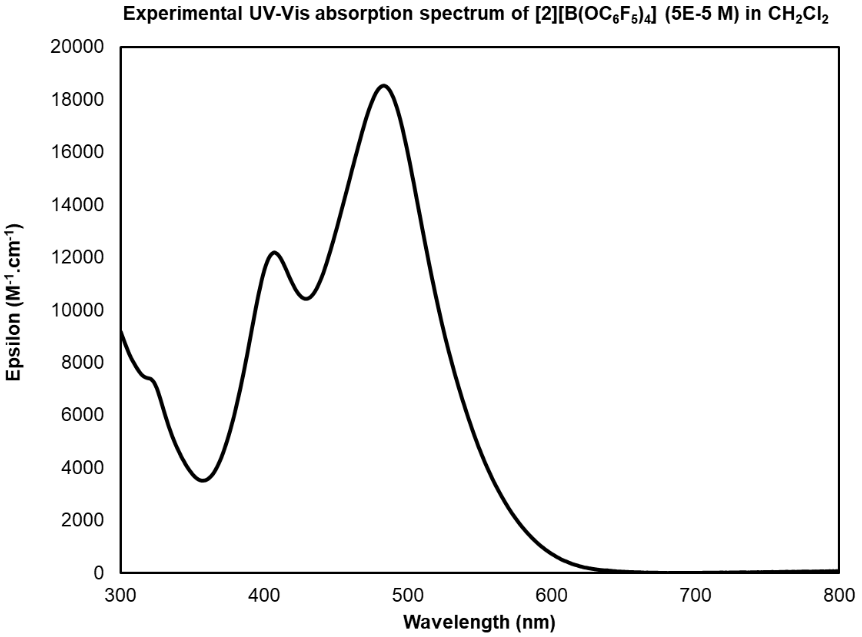

Figure S17: UV-Vis absorption spectrum of [**2**][$B(OC_6F_5)_4$].

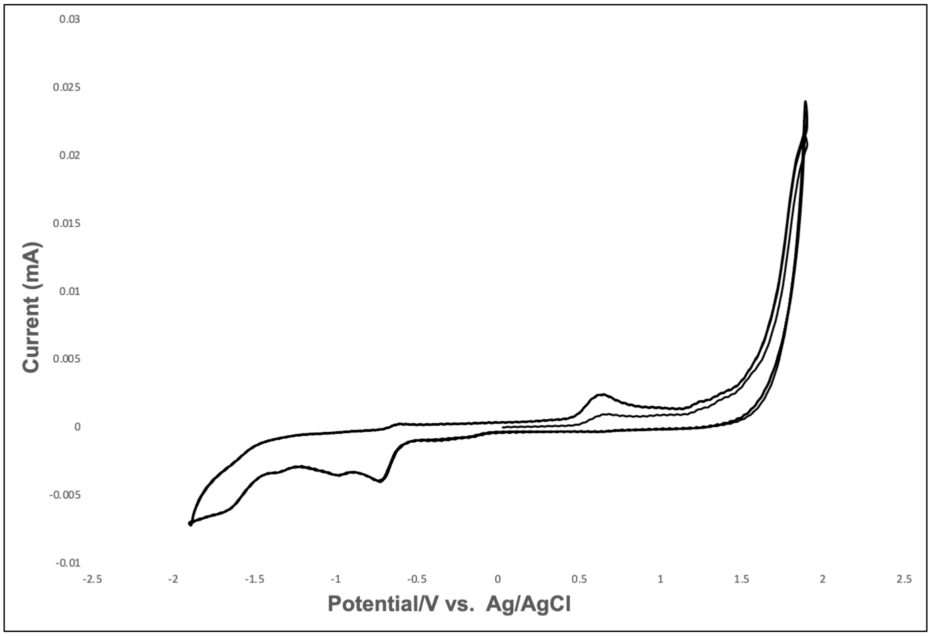

Figure S18: Cyclic voltammetry experiment of [**2**][$B(OC_6F_5)_4$] in 1,2-DFB (scan rate 100 mV/s, supporting electrolyte 0.1 M $N^nBu_4PF_6$, potential versus Ag/AgCl, scanned from -1.9 V to 1.9 V (3 sweeps).

2.3 Preparation of [2•DMAP][B(OC_6F_5)$_4$]

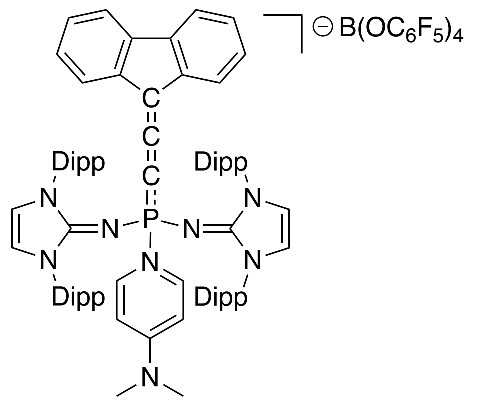

[**2**][B(OC_6F_5)$_4$] (50 mg, 28.2 mmol) was dissolved precooled (-40 °C) DCM (1 mL). A cooled (-40 °C) DCM solution of 4-dimethylaminopyridine (1.1 equiv, 3.8 mg, 31.1 mmol) was then added. The combination was stored overnight at -40 °C, then warmed to room temperature. Afterwards, all volatiles were removed *in vacuo*, and the resulting orange residue was washed with diethyl ether (3 x 1 ml), and benzene (3 x 1 ml). Removal of solvent afforded the title compound as a vibrant orange solid.

Yield: 33 mg (17.5 mmol, 62%).

^{1}H NMR (CD_2Cl_2, 400 MHz, 298 K) δ (ppm) = 7.97 (d, $^3J_{HH}$ = 7.7 Hz, 2H, CH fluorenyl), 7.75 (dd, $^3J_{HH}$ = 7.1. Hz, $^3J_{HP}$ = 8.2 Hz, 2H, DMAP *ortho*-H), 7.36 – 7.24 (m, 6H, CH Dipp; *para* and CH fluorenyl overlapped), 7.19 (t, $^3J_{HH}$ = 7.3 Hz, 2H, CH fluorenyl), 7.10 (dd, $^3J_{HH}$ = 7.8 Hz, 1.4 Hz, 4H, CH Dipp; *meta*), 7.06 (t, $^3J_{HH}$ = 7.3 Hz, 2H, CH fluorenyl), 6.97 (dd, $^3J_{HH}$ = 7.8 Hz, 1.4 Hz, 4H, CH Dipp; *meta*), 6.62 (s, 4H, N-C<u>H</u>=C<u>H</u>-N), 5.55 (d, $^3J_{HH}$ = 7.2 Hz, 2H, DMAP *m*-H), 3.03 (s, 6H, DMAP N(C<u>H</u>$_3$)$_2$), 2.92 (sept, $^3J_{HH}$ = 6.8 Hz, 4H, Dipp; C<u>H</u>(CH_3)$_2$), 2.52 (sept, $^3J_{HH}$ = 6.8 Hz, 4H, Dipp; C<u>H</u>(CH_3)$_2$), 1.10 (d, $^3J_{HH}$ = 6.8 Hz, 12H, Dipp; CH(C<u>H</u>$_3$)$_2$) 1.04 – 0.98 (m, 24H, Dipp; CH(C<u>H</u>$_3$)$_2$), 0.81 (d, $^3J_{HH}$ = 6.8 Hz, 12H, Dipp; CH(C<u>H</u>$_3$)$_2$).

^{13}C {^{1}H} NMR (CD_2Cl_2, 76 MHz, 298 K) δ (ppm) = 155.5 (DMAP *para*-C), 146.8, (Dipp: *ortho*) 146.3, (Dipp: *ortho*), 143.5 (d, $^2J_{CP}$ = 27.4 Hz, N-C-N), 144.4 (d, $^2J_{CP}$ = 43.5 Hz, P=C=<u>C</u>=C), 142.4 (quaternary carbon, fluorenyl), 142.38 (quaternary carbon, fluorenyl), 130.7 (quaternary carbon, fluorenyl), 138.64 ($^3J_{CP}$ = 6 Hz DMAP *meta*-C),138.64 ($^3J_{CP}$ = 6 Hz DMAP *ortho*-C), 132.4 (Dipp: *ipso*), 130.9 (Dipp; *para*), 125.4 (CH Dipp: *meta*), 125.1 (CH Dipp: *meta*), 124.3 (CH, fluorenyl), 120.1 (CH, fluorenyl), 119.6 (N-CH=CH-N), 119.3 (CH, fluorenyl), 118.7 (CH, fluorenyl), 106.8 ($^2J_{CP}$ = 5.3 Hz DMAP *ortho*-C), 106.8 ($^3J_{CP}$ = 5.3 Hz DMAP *meta*-C), 90.5 (d, $^1J_{CP}$ = 214.1 Hz, P=<u>C</u>=C=C), 75.8 (d, $^3J_{CP}$ = 11.6 Hz, P=C=C=<u>C</u>), 40.3 (DMAP; N(<u>C</u>H$_3$)$_2$), 29.4 (Dipp; <u>C</u>H(CH_3)$_2$), 29.3 (Dipp; <u>C</u>H(CH_3)$_2$), 25.4 (Dipp; CH(<u>C</u>H$_3$)$_2$), 24.9 (Dipp; CH(<u>C</u>H$_3$)$_2$), 23.4 (Dipp; CH(<u>C</u>H$_3$)$_2$), 22.9 (Dipp; CH(<u>C</u>H$_3$)$_2$).

Note: C-F carbons (*ortho/meta/para*) are too low in intensity, they can be observed in the baseline, but can't be definitively characterized. The data was acquired with 100,000 scans, and a concentrated sample.

^{11}B NMR (CD_2Cl_2, 96 MHz, 298 K) δ (ppm) = -1.2.

^{19}F NMR (CD_2Cl_2, 376 MHz, 298 K) δ (ppm) = -157.9 (d, $^3J_{FF}$ = 19 Hz, 8F, CF B(OC_6F_5)$_4$: *ortho*), -168.6 (t, $^3J_{FF}$ = 21 Hz, 8F, CF B(OC_6F_5)$_4$: *meta*), -172.6 (t, $^3J_{FF}$ = 22 Hz, 4F, CF B(OC_6F_5)$_4$: *para*).

^{31}P NMR (CD_2Cl_2, 162 MHz, 298 K) δ (ppm) = -30.4 (t, $^{3}J_{HP}$ = 8.2 Hz).

HR-ESI-MS: Calculated for $[C_{76}H_{90}N_8P]^+$ ([**2•DMAP**]$^+$) = 1145.7026 found: m/z = 1041.6256, Calculated for $[B(OC_6F_5)_4]^-$ = 742.9570, found: m/z = 742.9589.

The positive mass peak found corresponds to the calculated mass of [**2•H$_2$O**]$^+$ = 1041.6288.

Single crystal X-ray diffraction analysis: Orange single crystals of **[2•DMAP][B(OC$_6$F$_5$)$_4$]** suitable for X-ray diffraction were obtained by slow evaporation of a concentrated ether solution.

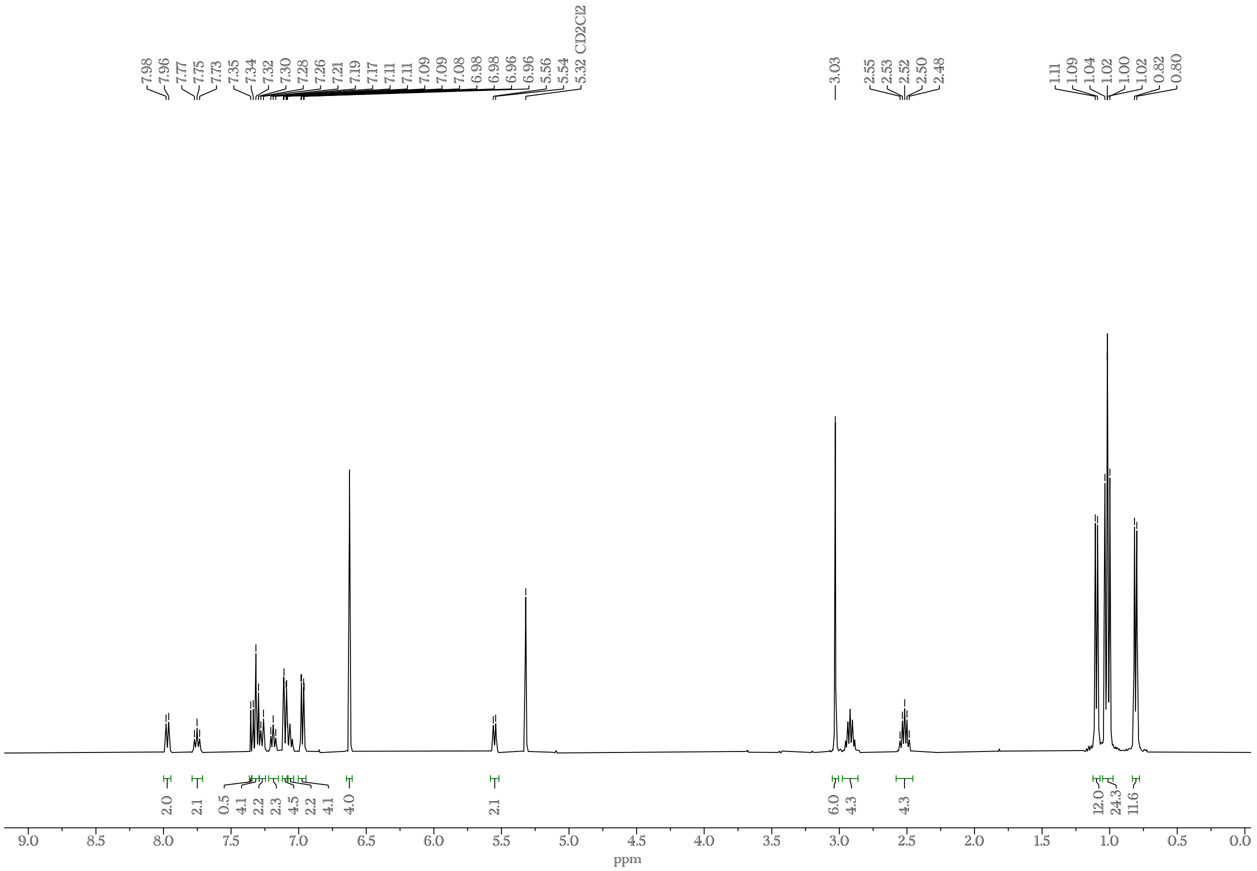

Figure S19: ^{1}H NMR (400 MHz, 298 K) spectrum of [**2•DMAP**][$B(OC_6F_5)_4$] in CD_2Cl_2.

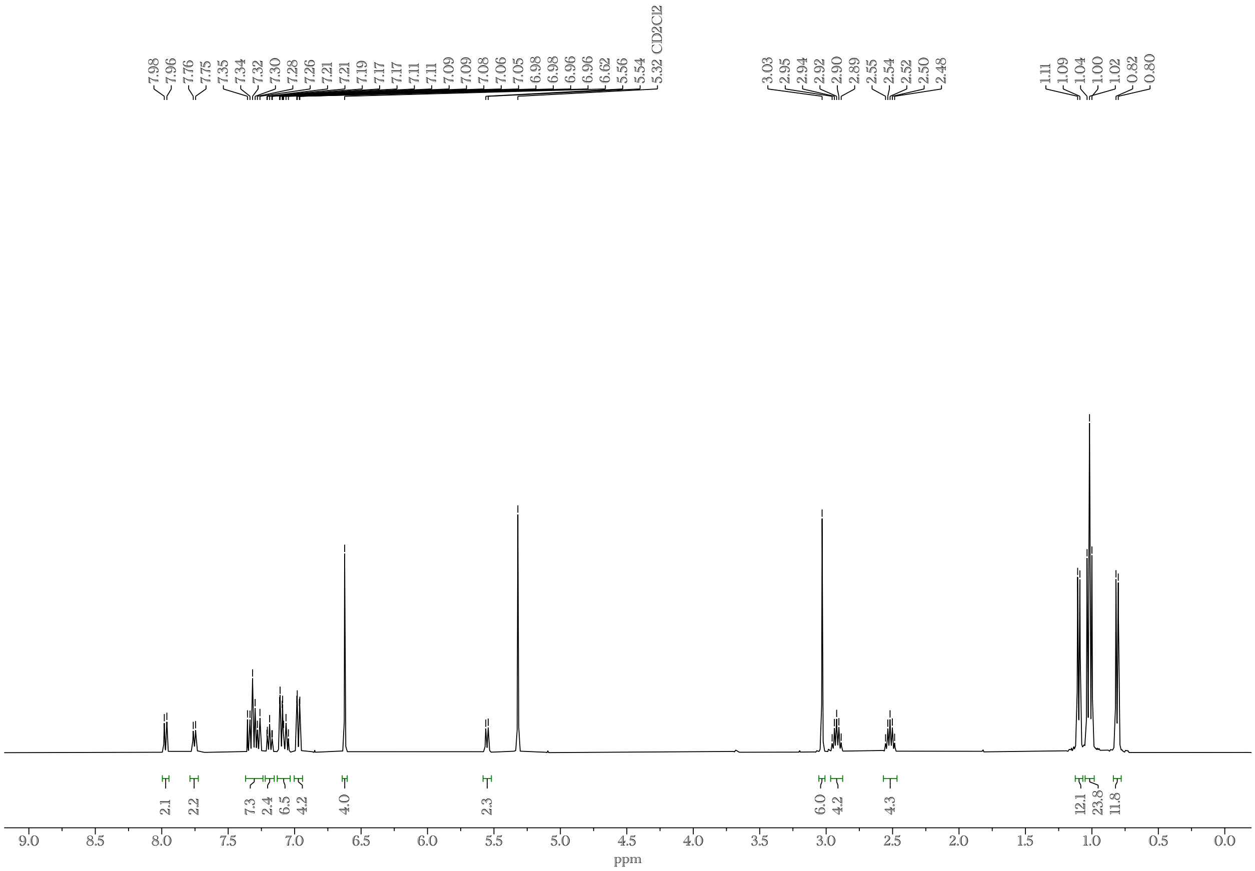

Figure S20: $^{1}H\{^{31}P\}$ NMR (400 MHz, 298 K) spectrum of [**2•DMAP**][$B(OC_6F_5)_4$] in CD_2Cl_2.

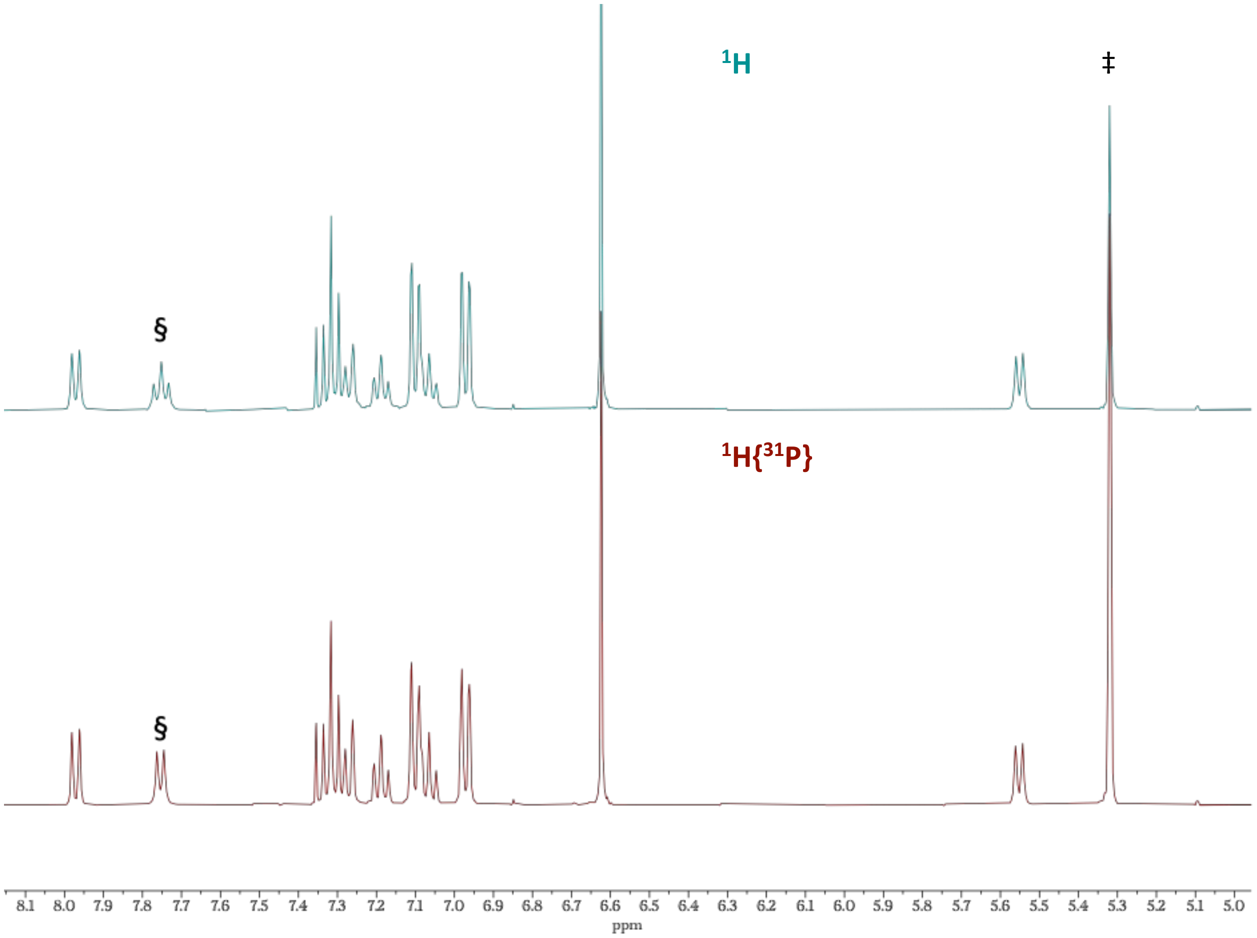

Figure S21: Stacked ^{1}H NMR (top) and ^{1}H{^{31}P} NMR (bottom) (400 MHz, 298 K, CD_2Cl_2) spectra of [**2•DMAP**][$B(OC_6F_5)_4$]. § marks the *o*-DMAP proton resonance. ‡ denotes the residual solvent peak.

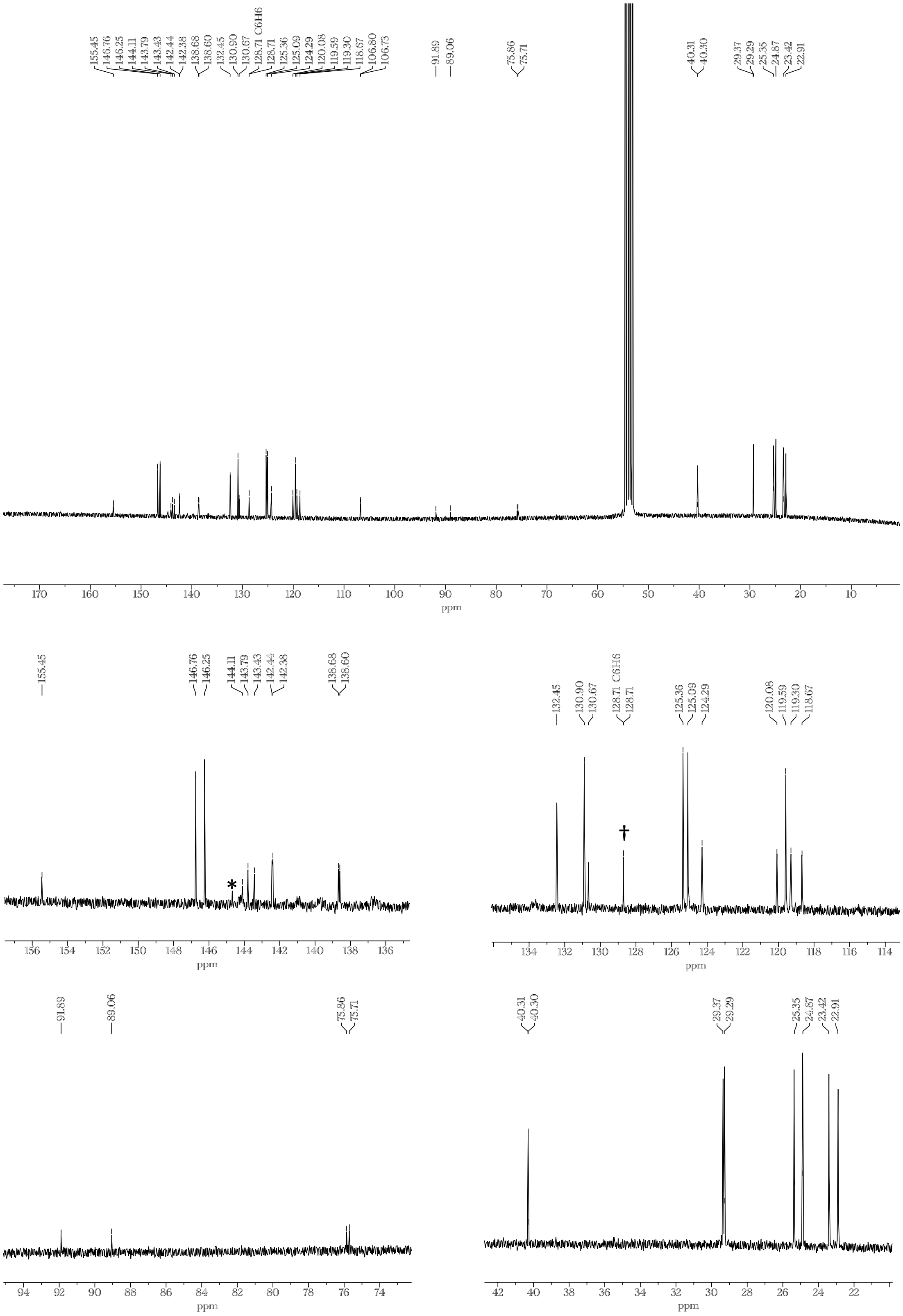

Figure S22: $^{13}C\ \{^{1}H\}$ NMR (76 MHz, 298 K) spectrum of [**2•DMAP**][$B(OC_6F_5)_4$] in CD_2Cl_2. * peak at 144.7 ppm below autodetection limit. † trace C_6H_6 from workup.

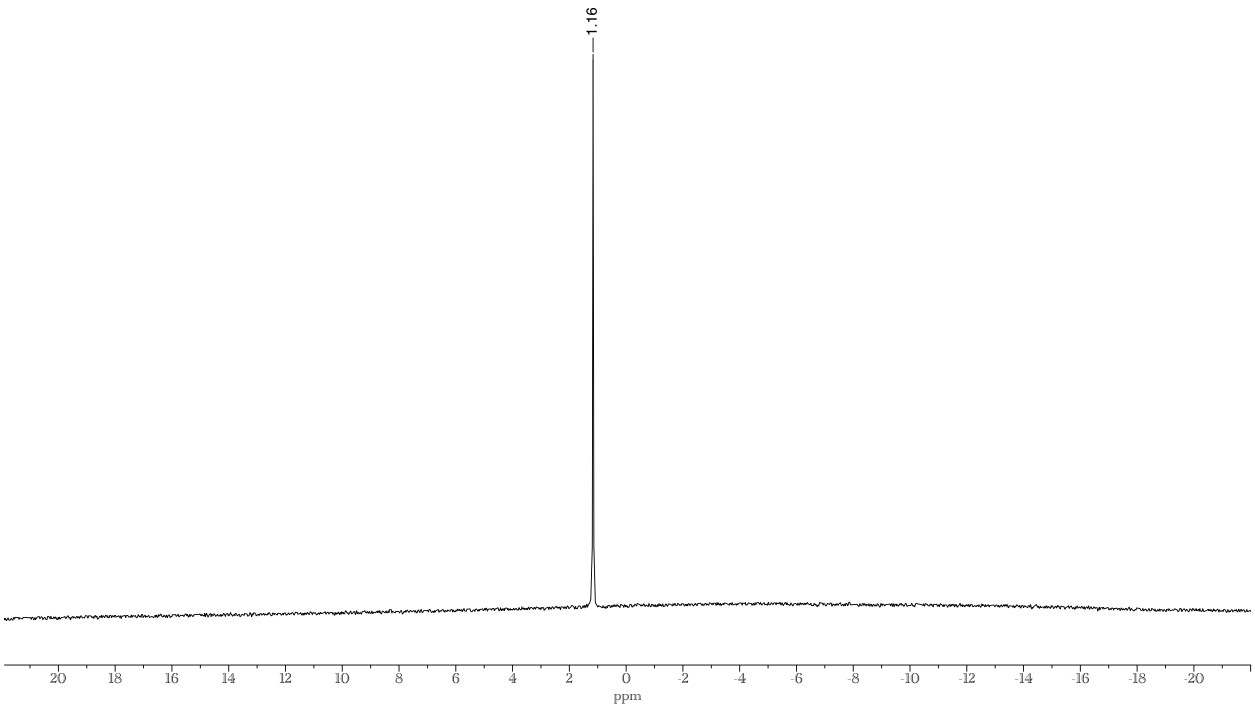

Figure S23: ^{11}B NMR (128 MHz, 298 K) spectrum of [**2•DMAP**][B(OC_6F_5)$_4$] in CD_2Cl_2.

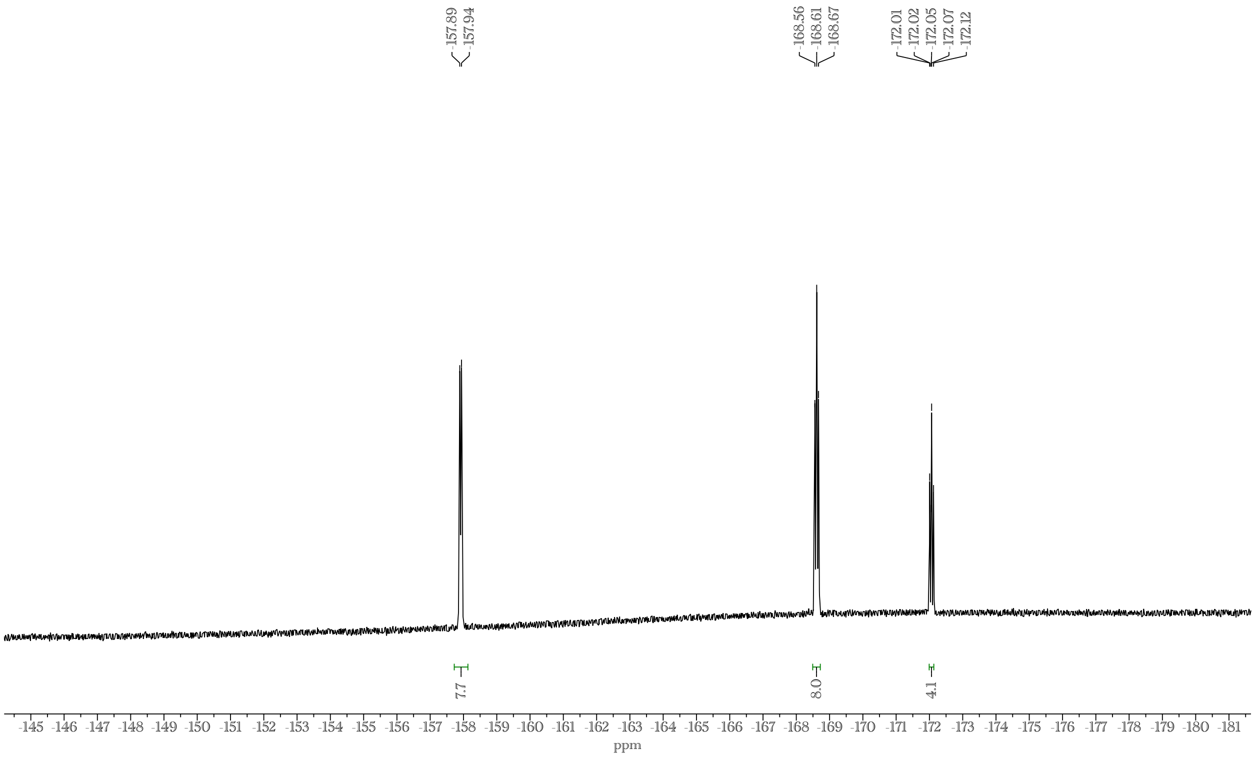

Figure S24: ^{19}F NMR (376 MHz, 298 K) spectrum of [**2•DMAP**][B(OC_6F_5)$_4$] in CD_2Cl_2.

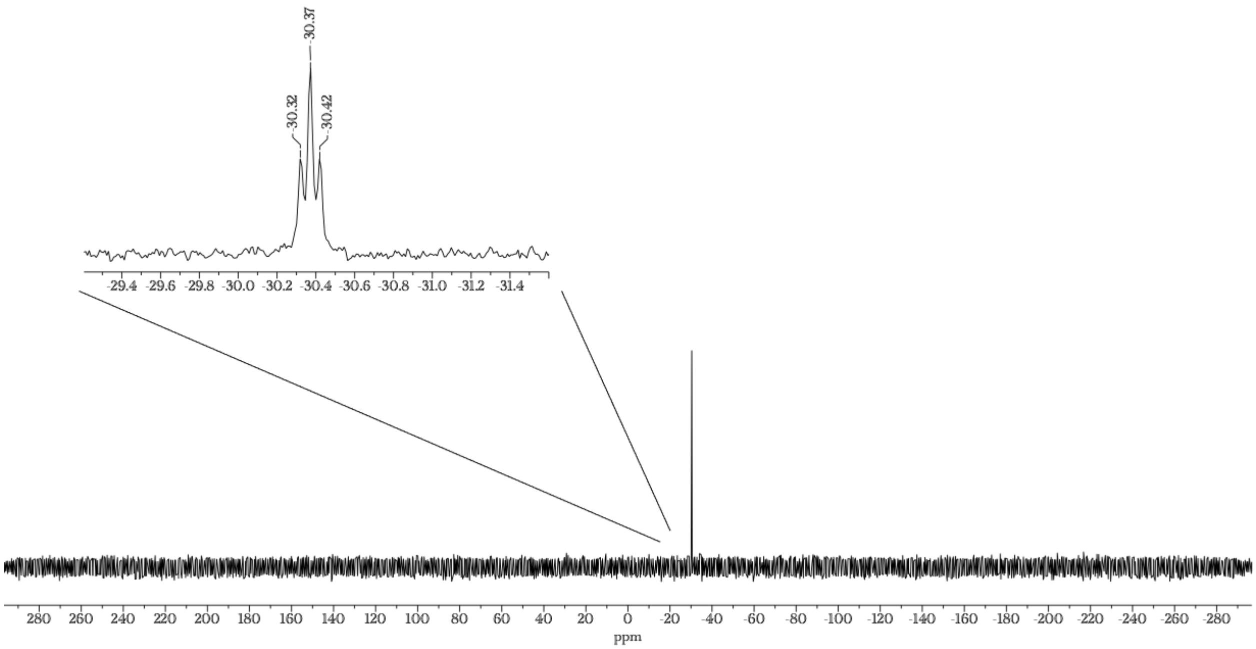

Figure S25: ^{31}P NMR (162 MHz, 298 K) spectrum of [**2•DMAP**][B(OC_6F_5)$_4$] in CD_2Cl_2.

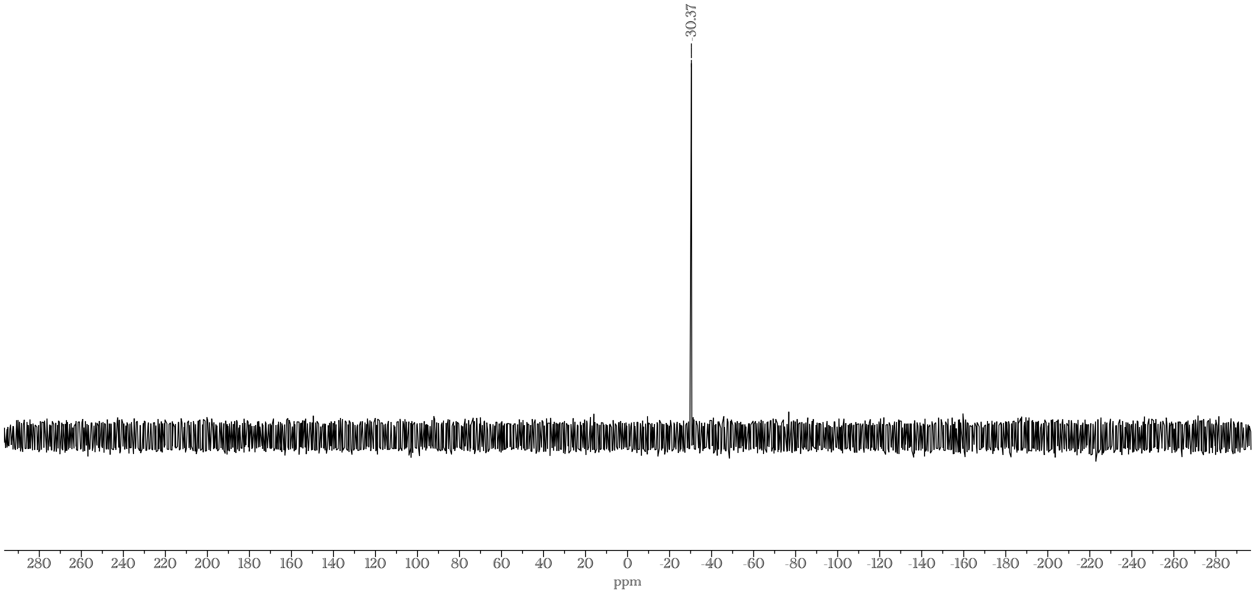

Figure S26: ^{31}P{^{1}H} NMR (162 MHz, 298 K) spectrum of [**2•DMAP**][B(OC_6F_5)$_4$] in CD_2Cl_2.

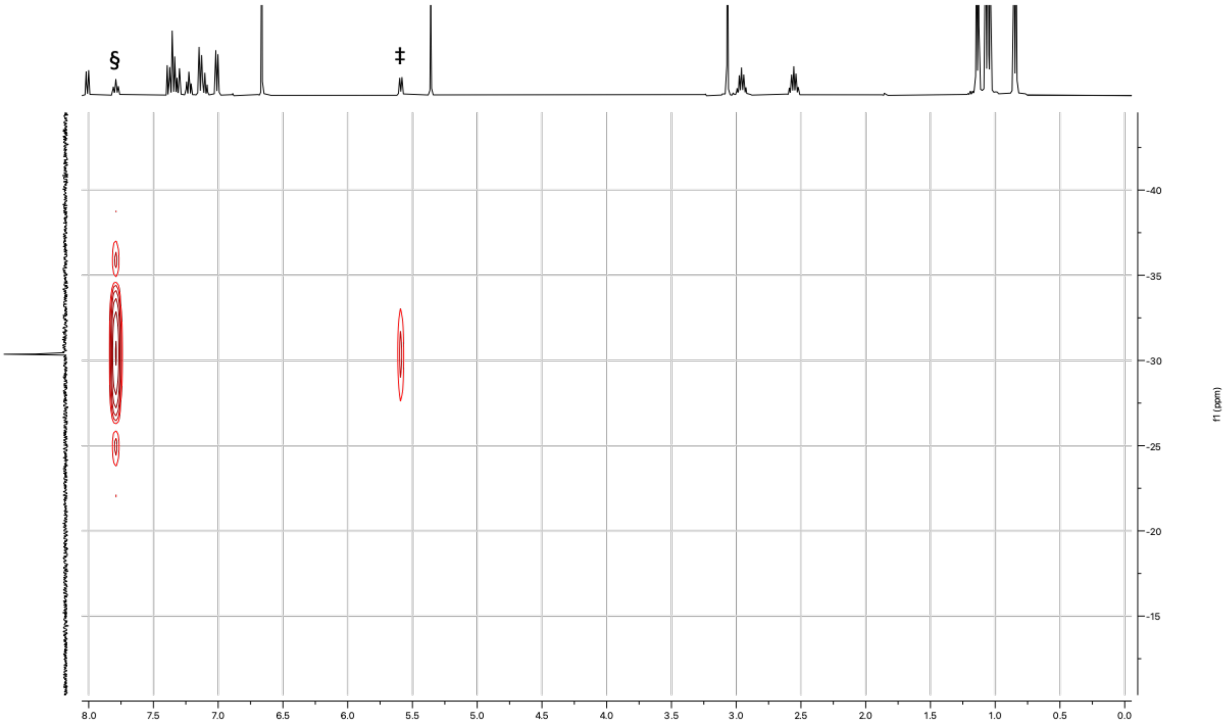

Figure S27: ^{1}H/^{31}P HMBC NMR spectrum of [**2•DMAP**][$B(OC_6F_5)_4$] in CD_2Cl_2. § marks the *o*-DMAP proton resonance, and ‡ marks the *m*-DMAP proton resonance.

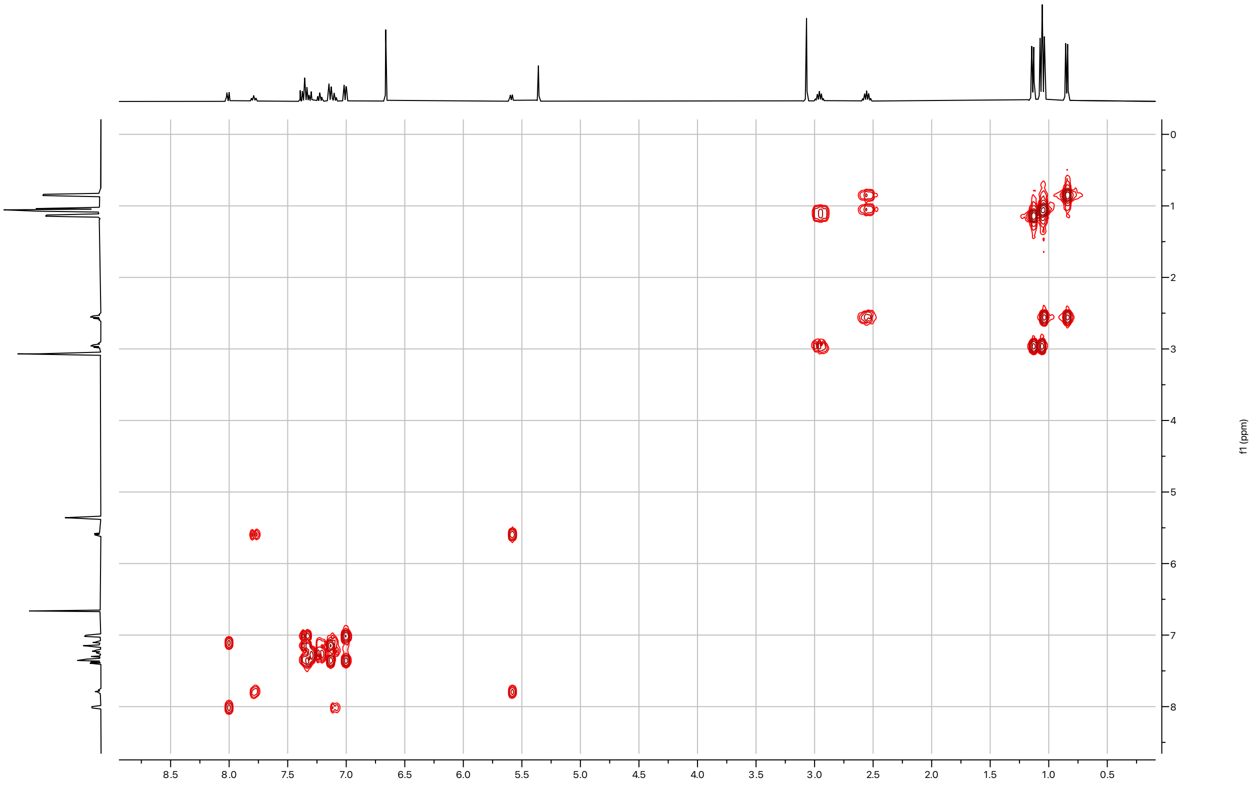

Figure S28: ^{1}H COSY NMR spectrum of [**2•DMAP**][$B(OC_6F_5)_4$] in CD_2Cl_2.

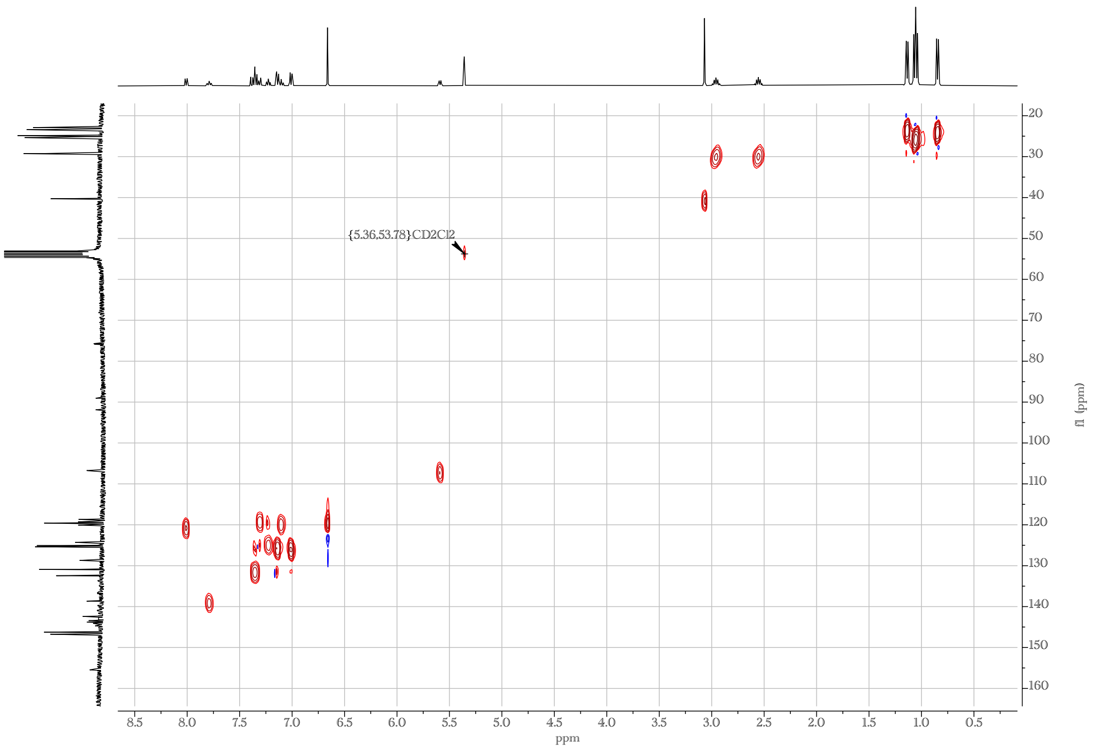

Figure S29: $^{1}H/^{13}C\{^{1}H\}$ HSQC NMR spectrum of [**2•DMAP**][$B(OC_6F_5)_4$] in CD_2Cl_2.

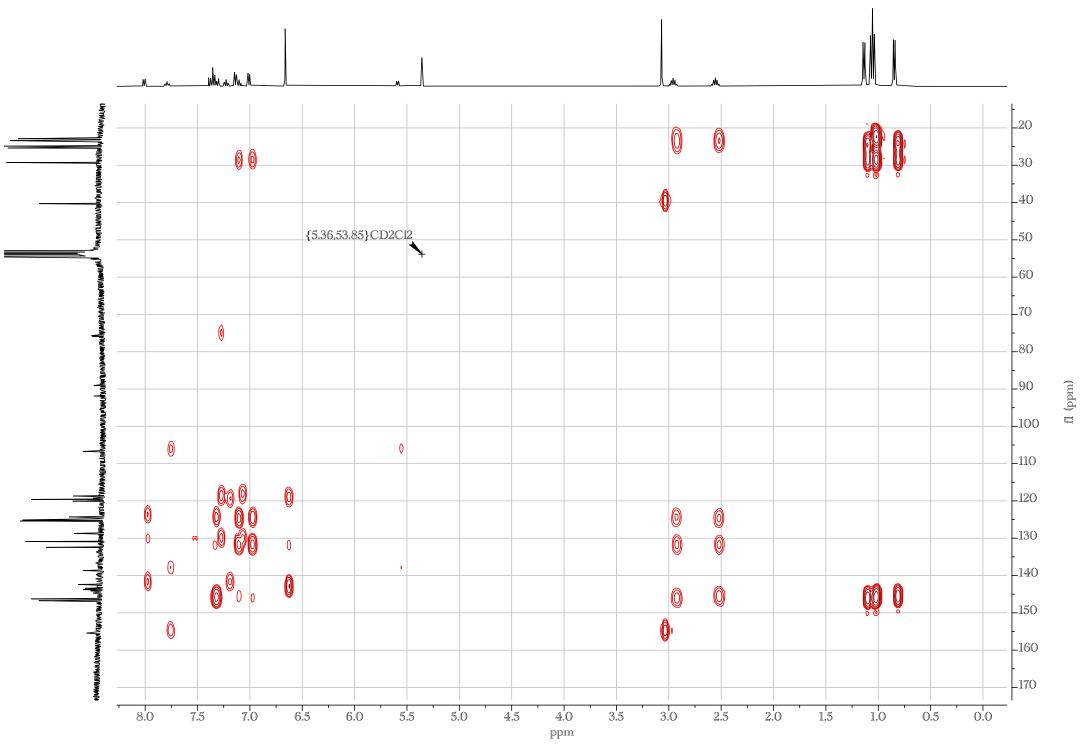

Figure S30: $^{1}H/^{13}C\{^{1}H\}$ HMBC NMR spectrum of [**2•DMAP**][$B(OC_6F_5)_4$] in CD_2Cl_2.

2.4 Preparation of azaphosphete salt [3][B(OC$_6$F$_5$)$_4$]

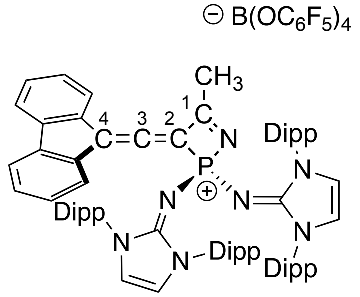

[**2**][B(OC$_6$F$_5$)$_4$] (25 mg, 0.0142 mmol, 1.00 equiv) was dissolved in pre-cooled DCM (-40°C), and an excess of acetonitrile (75 uL, 1.4 mmol, 100 equiv) was added. The reaction was allowed to heat to 55°C for 72 hours. Once cooled, the solvent was evaporated in vacuo and the residue was washed with pentane (3 x 2 mL). The title compound was isolated as a light orange powder.

Yield: 25 mg (0.0142 mmol, quantitative).

^{1}H NMR (CDCl$_3$, 700 MHz, 298 K) δ (ppm) = 7.75 (d, $^{3}J_{HH}$ = 7.8 Hz, 2H, CH fluorenyl), 7.43 (t, $^{3}J_{HH}$ = 7.8 Hz, 2H, CH fluorenyl), 7.39 (t, $^{3}J_{HH}$ = 7.8 Hz, 4H, CH Dipp; *para*), 7.24 (t, $^{3}J_{HH}$ = 7.5 Hz, 2H, CH fluorenyl), 7.16 (d, $^{3}J_{HH}$ = 7.9 Hz, 2H, CH fluorenyl), 7.14 (d, $^{3}J_{HH}$ = 7.8 Hz, 4H, CH Dipp; *meta*), 7.07 (d, $^{3}J_{HH}$ = 7.8 Hz, 4H, CH Dipp; *meta*), 6.63 (s, 4H, N-C<u>H</u>=C<u>H</u>=N), 2.62 (sept, $^{3}J_{HH}$ = 6.9 Hz, 4H, C<u>H</u>(CH$_3$)$_2$), 2.51 (sept, $^{3}J_{HH}$ = 6.8 Hz, 4H, C<u>H</u>(CH$_3$)$_2$), 1.35, (d, $^{4}J_{HP}$ = 4.1 Hz, 3H, P-N=C(C<u>H</u>$_3$)), 1.16 (d, $^{3}J_{HH}$ = 6.8 Hz, 12H, CH(C<u>H</u>$_3$)$_2$), 1.02 (d, $^{3}J_{HH}$ = 6.8 Hz, 12H, CH(C<u>H</u>$_3$)$_2$), 0.87 (d, $^{3}J_{HH}$ = 6.9 Hz, 12H, CH(C<u>H</u>$_3$)$_2$), 0.76 (d, $^{3}J_{HH}$ = 6.8 Hz, 12H, CH(C<u>H</u>$_3$)$_2$).

^{11}B NMR (CDCl$_3$, 128 MHz, 297 K) δ (ppm) = 1.2

^{13}C {^{1}H} NMR (CDCl$_3$, 101 MHz, 298 K) δ (ppm) = 187.9 (d, $^{2}J_{CP}$ = 24 Hz, P-C=<u>C</u>=CR$_2$, position 3), 187.3 (d, $^{2}J_{CP}$ = 25 Hz, P-N=<u>C</u>(CH$_3$), position 1), 145.9, (C$_q$ Dipp: *ortho*), 145.4 (C$_q$ Dipp: *ortho*), 143.0 (d, $^{2}J_{CP}$ = 24 Hz, N-C-N), 142.3 (dm, $^{1}J_{CF}$ = 238 Hz, CF B(OC$_6$F$_5$)$_4$: *ortho*), 139.6 (C$_q$ fluorenyl), 137.8 (dm, $^{1}J_{CF}$ = 245 Hz, CF B(OC$_6$F$_5$)$_4$: *para*), 136.1 (J_{CP} = 10 Hz, C$_q$ fluorenyl), 135.0 (dm, $^{1}J_{CF}$ = 243 Hz, CF B(OC$_6$F$_5$)$_4$: *meta*), 133.5 (m, B(OC$_6$F$_5$)$_4$: *ipso*), 131.6 (Dipp: *ipso*) 130.7 (CH Dipp: *para*),130.0 (CH fluorenyl), 127.0 (CH fluorenyl), 125.0 and 124.9 (CH Dipp; *meta*), 123.7 (CH fluorenyl), 121.2 (CH fluorenyl), 120.5 (d, $^{1}J_{CP}$ = 70 Hz, P-<u>C</u>=C=CR$_2$, position 2), 118.5 (N-<u>C</u>H=<u>C</u>H=N), 110.1 (d, $^{3}J_{CP}$ = 20 Hz, P-C=C=<u>C</u>R$_2$, position 4) 29.0 (<u>C</u>H(CH$_3$)$_2$), 29.0(<u>C</u>H(CH$_3$)$_2$), 25.1 (CH(<u>C</u>H$_3$)$_2$), 24.6 (CH(<u>C</u>H$_3$)$_2$), 23.9 (CH(<u>C</u>H$_3$)$_2$), 22.5 (CH(<u>C</u>H$_3$)$_2$), 21.1 (d, $^{3}J_{CP}$ = 42 Hz, P-N=C(<u>C</u>H$_3$).

^{19}F NMR (CDCl$_3$, 282 MHz, 298 K) δ (ppm) = -157.1 (d, $^{3}J_{FF}$ = 19.8 Hz, 8F, CF B(OC$_6$F$_5$)$_4$: *ortho*), -163.4 – -169.8 (m, 8F, CF B(OC$_6$F$_5$)$_4$: *meta*), -171.6 (t, , $^{3}J_{FF}$ = 22.4 Hz, 4F, CF B(OC$_6$F$_5$)$_4$: *para*).

^{31}P NMR (CDCl$_3$, 101 MHz, 298 K) δ (ppm) = -18.7

HR-ESI-MS: Calculated for [C$_{71}$H$_{83}$N$_7$P]$^+$ ([**3**]$^+$) *m/z* = 1064.6442, found: *m/z* =1064.6452.

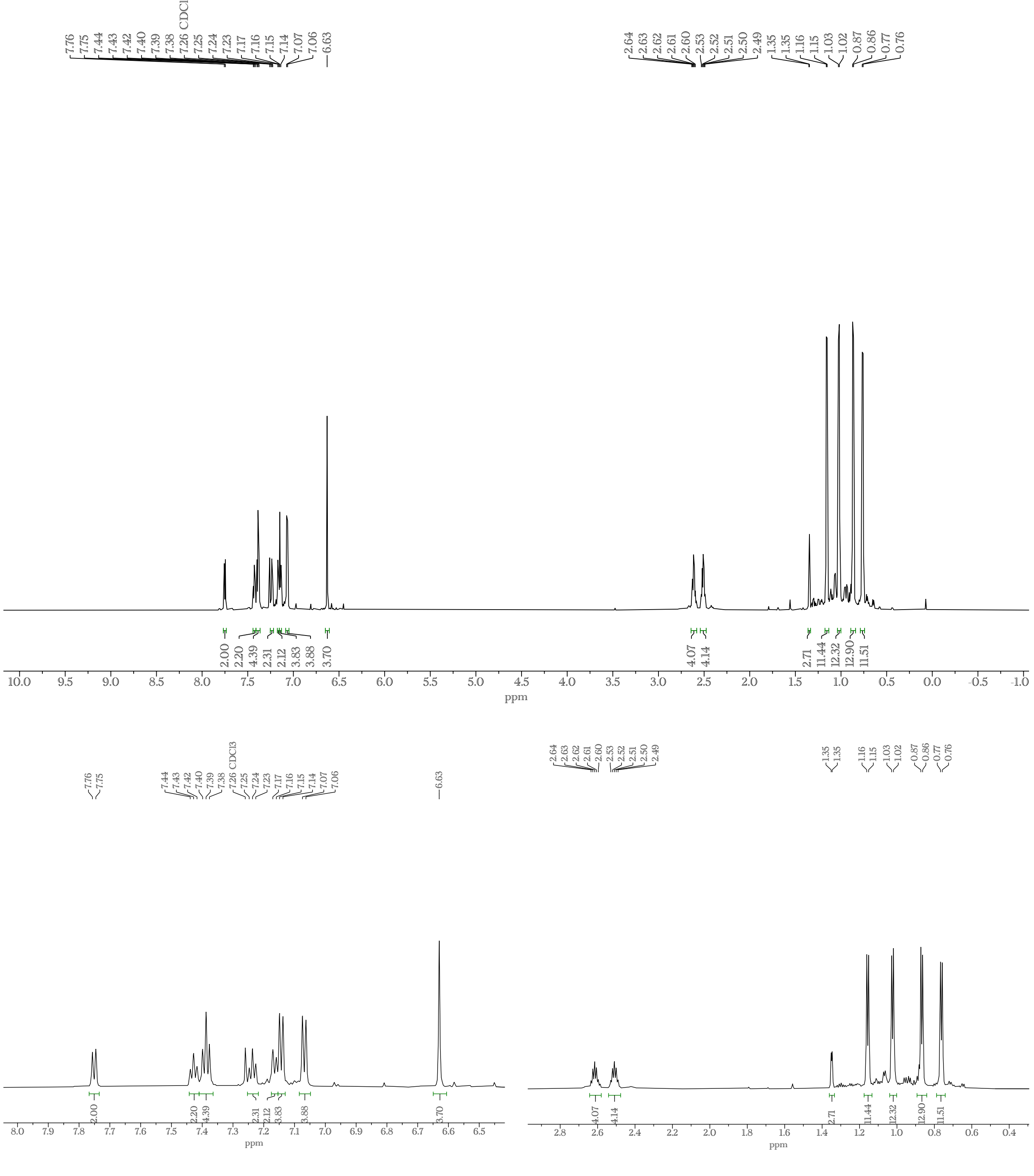

Figure S31: ^{1}H NMR (700 MHz, 298 K, $CDCl_3$) spectrum of [**3**][$B(OC_6F_5)_4$].

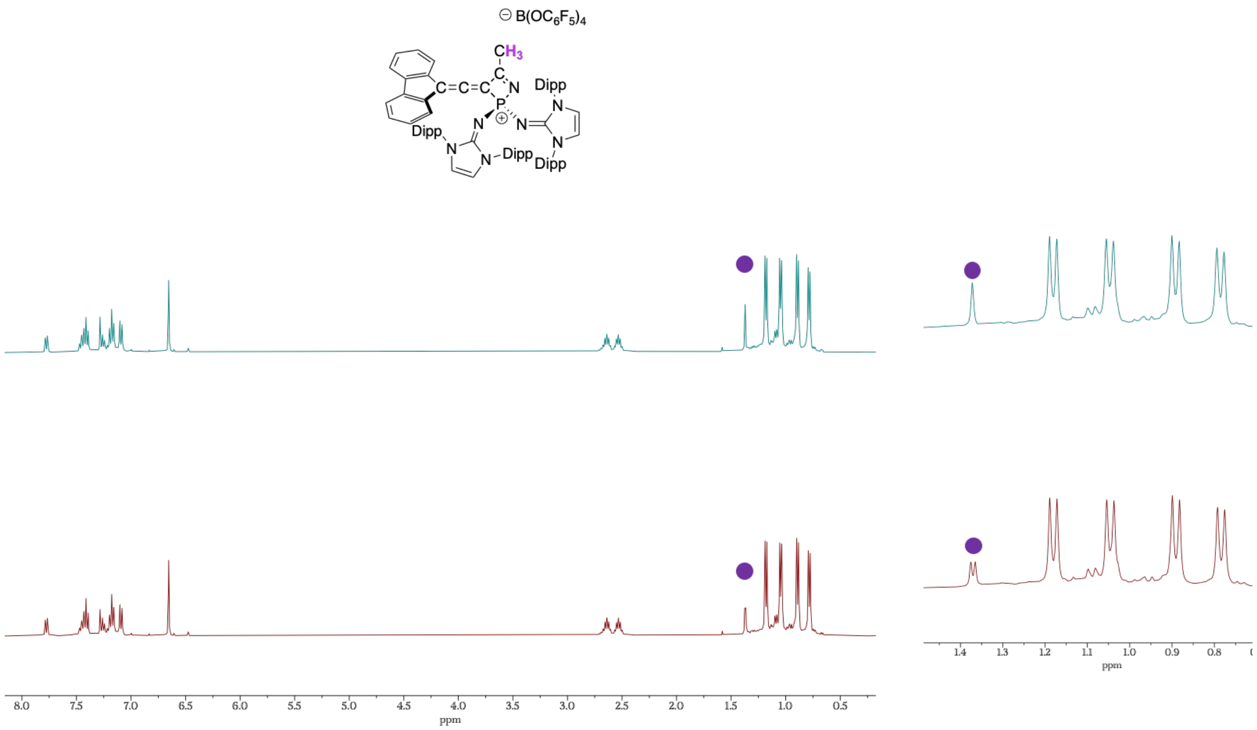

Figure S32: Stacked ^{1}H {^{31}P} NMR (top) and ^{1}H NMR (bottom) (400 MHz, 298 K, $CDCl_3$) spectra of [**3**][$B(OC_6F_5)_4$]. The purple circle marks the -CH_3 proton resonance.

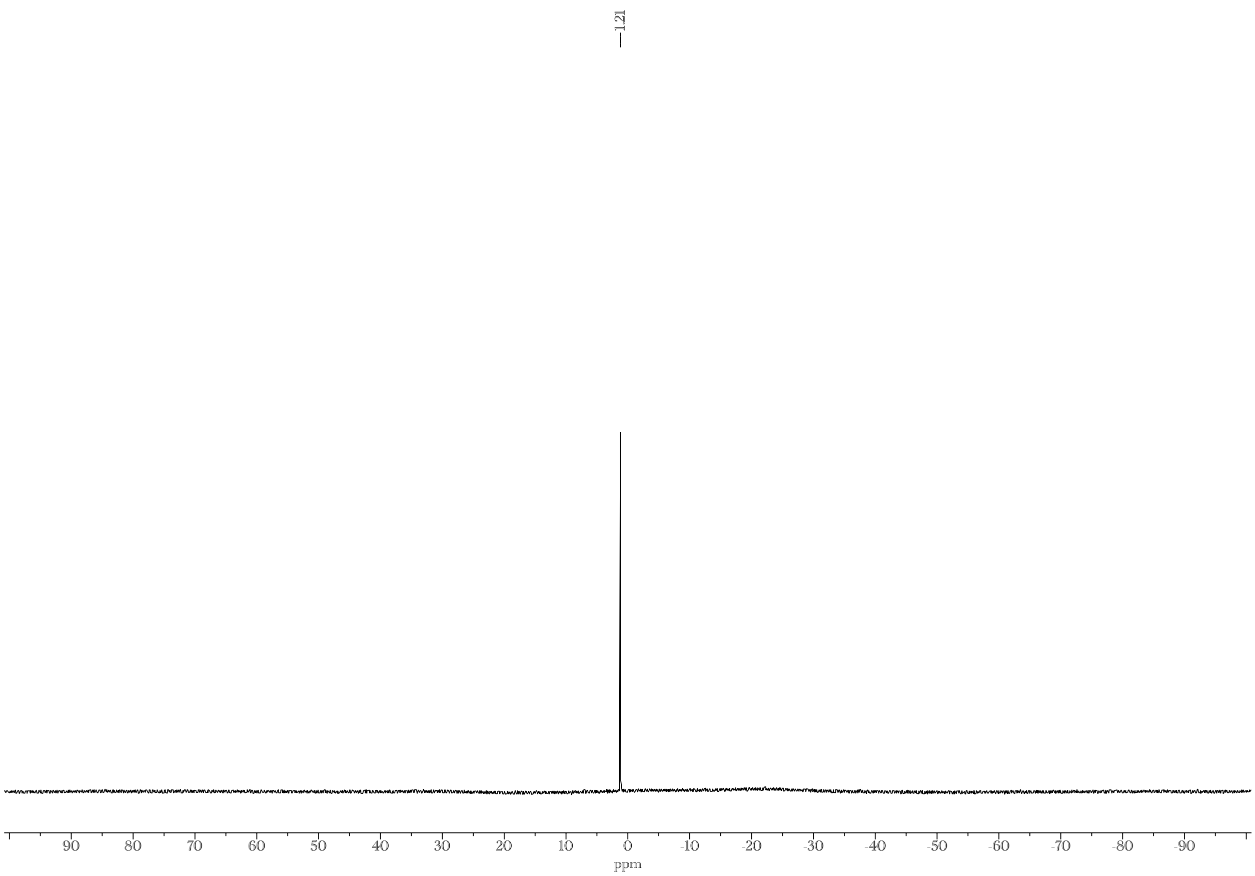

Figure S33: ^{11}B NMR (128 MHz, 297 K) spectrum of [**3**][$B(OC_6F_5)_4$] in $CDCl_3$.

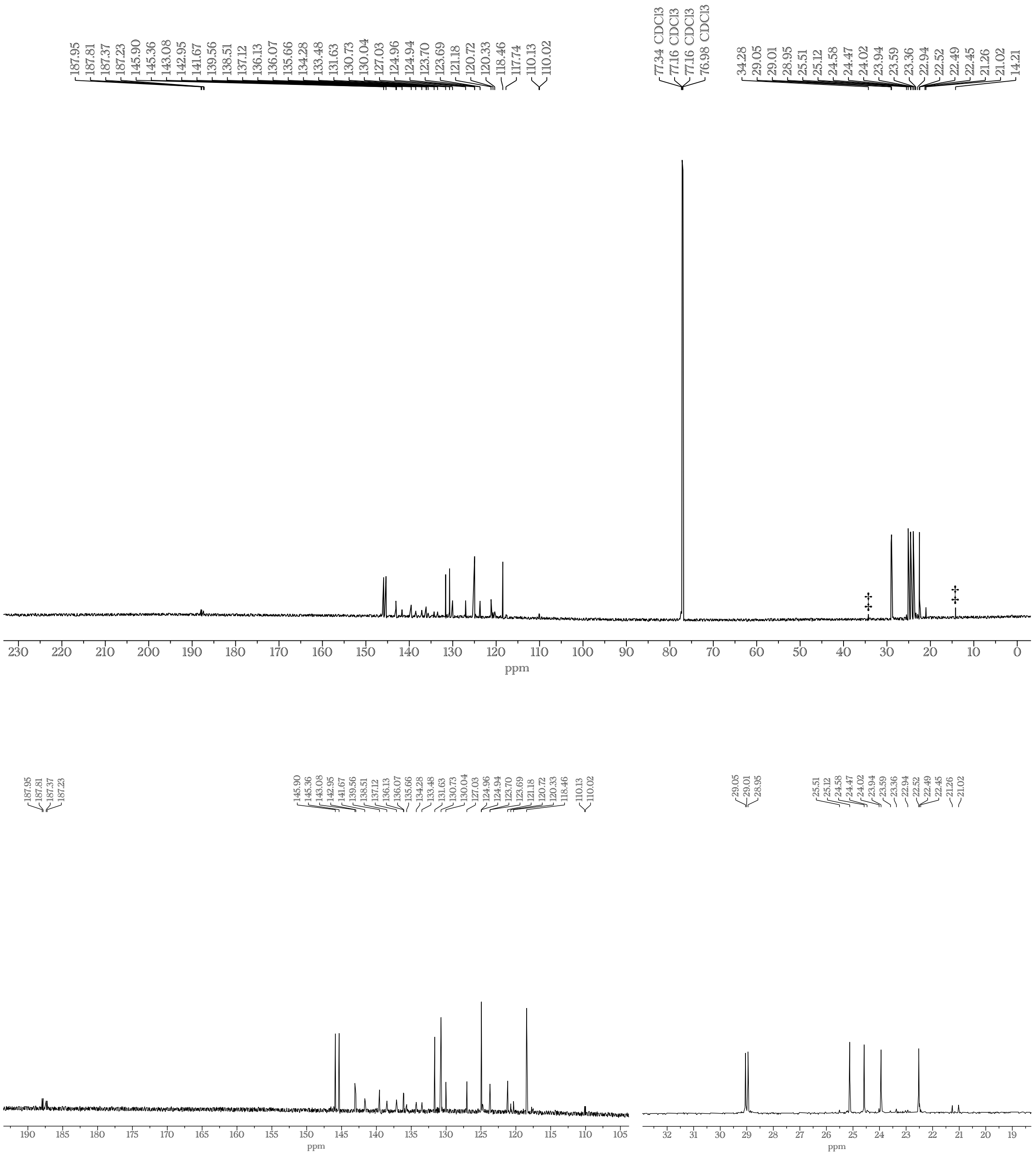

Figure S34: $^{13}C\{^{1}H\}$NMR (168 MHz, 298 K) spectrum of [**3**][B(OC_6F_5)$_4$] in $CDCl_3$. ‡ corresponds to trace pentane.

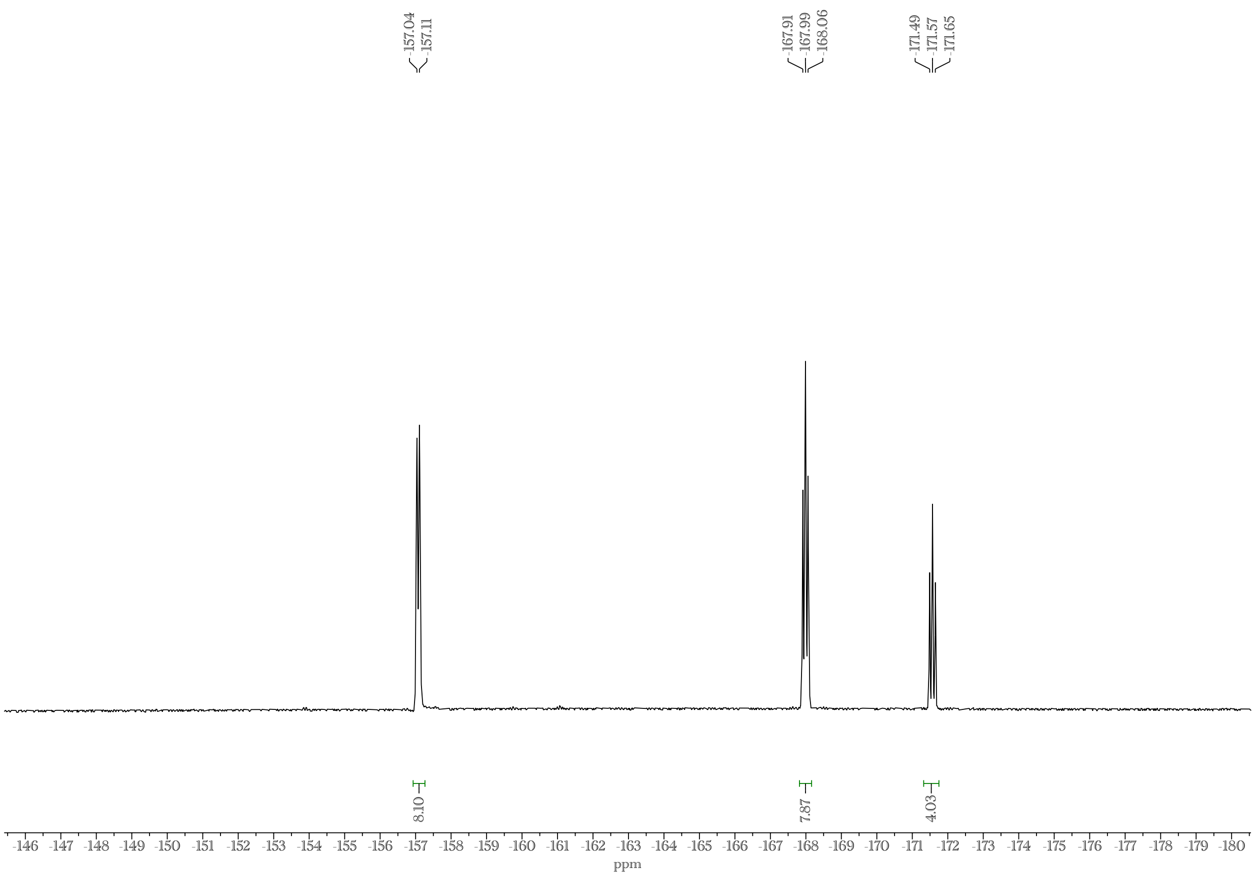

Figure S35: ^{19}F NMR (282 MHz, 296K) spectrum of [**3**][$B(OC_6F_5)_4$] in $CDCl_3$.

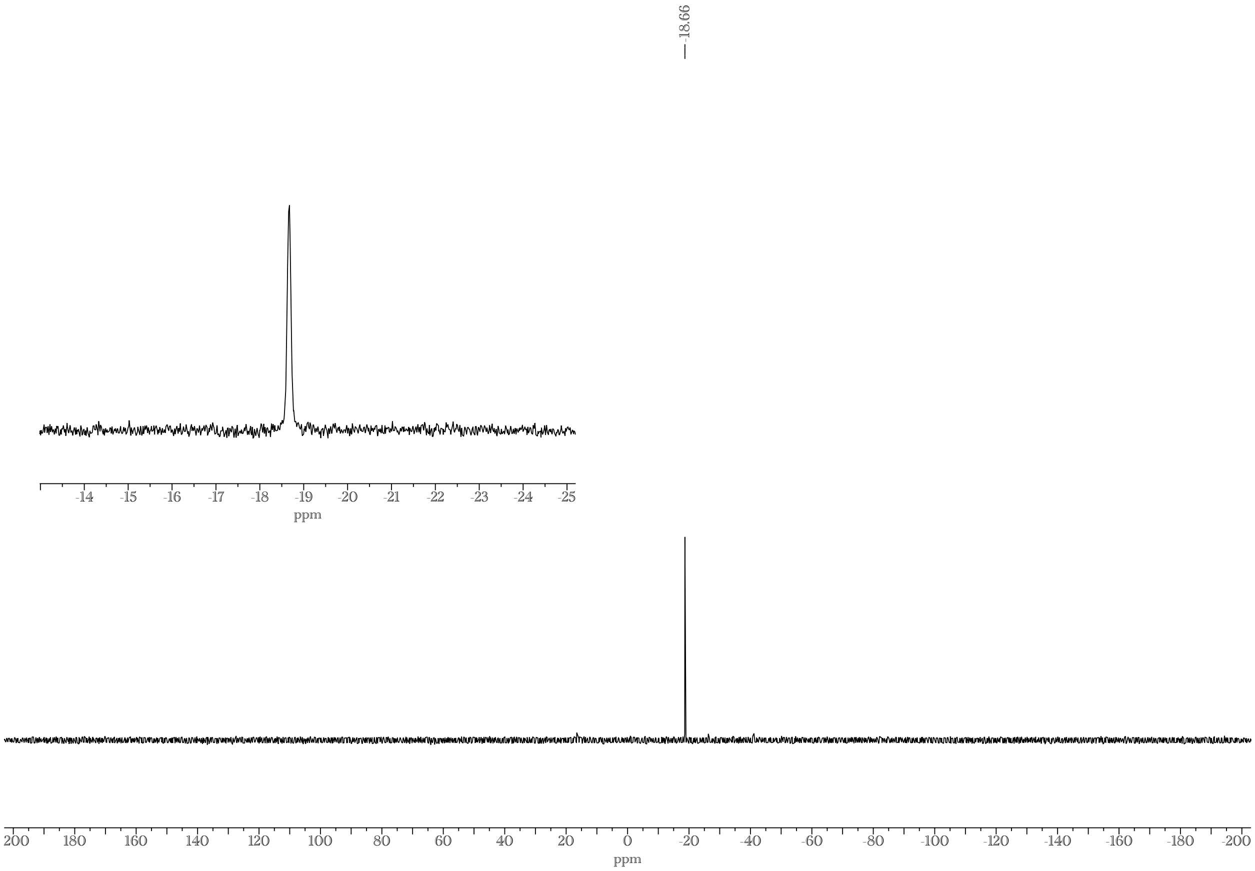

Figure S36: $^{31}P\{^{1}H\}$ NMR (162 MHz, 298 K) and ^{31}P NMR spectrum (zoom in) of [**3**][$B(OC_6F_5)_4$] in $CDCl_3$.

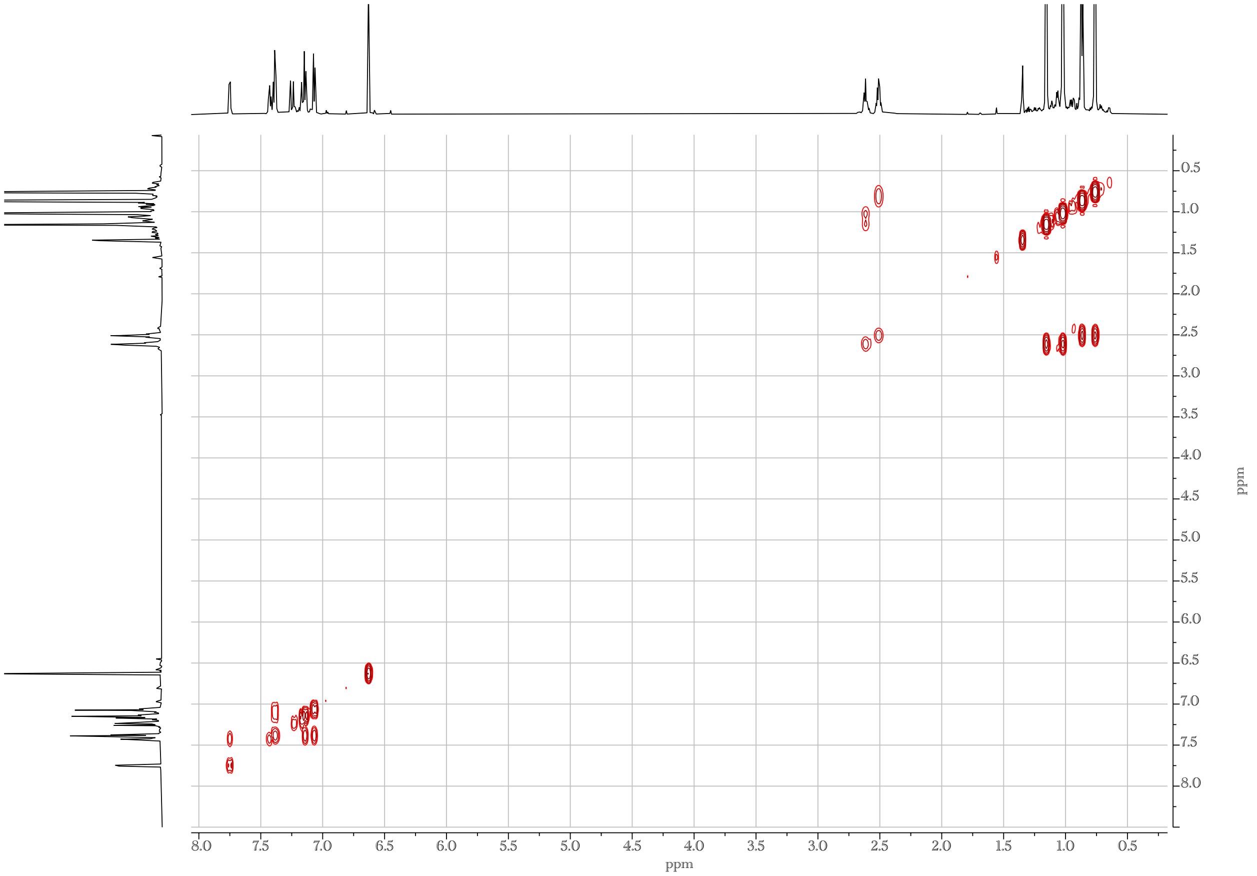

Figure S37: $^1H/^1H$ COSY NMR spectrum of [**3**][$B(OC_6F_5)_4$] in $CDCl_3$.

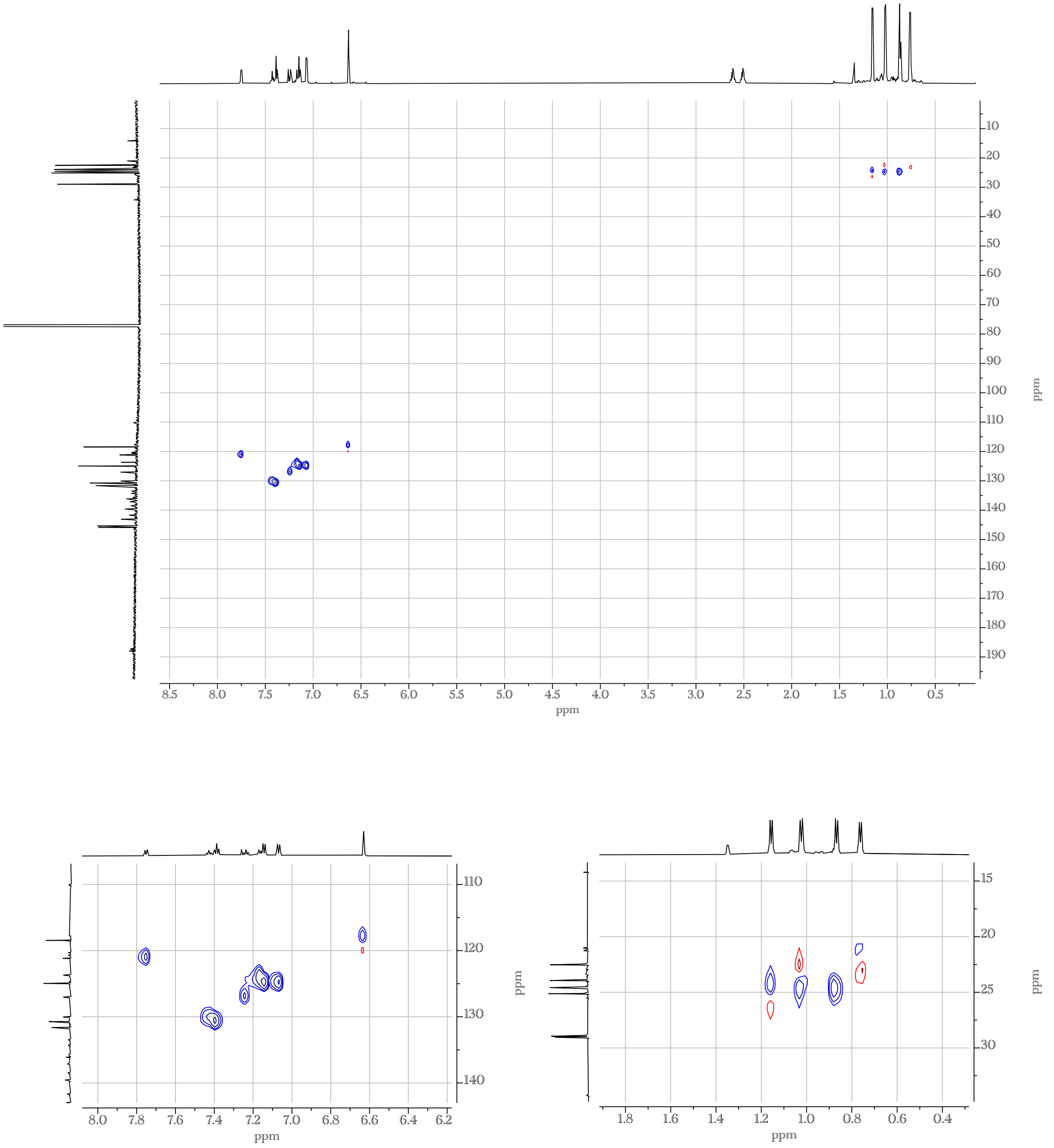

Figure S38: $^{1}H/^{13}C\{^{1}H\}$ HSQC DEPT NMR spectrum (with several zoomed perspectives) of [**3**][$B(OC_6F_5)_4$] in $CDCl_3$.

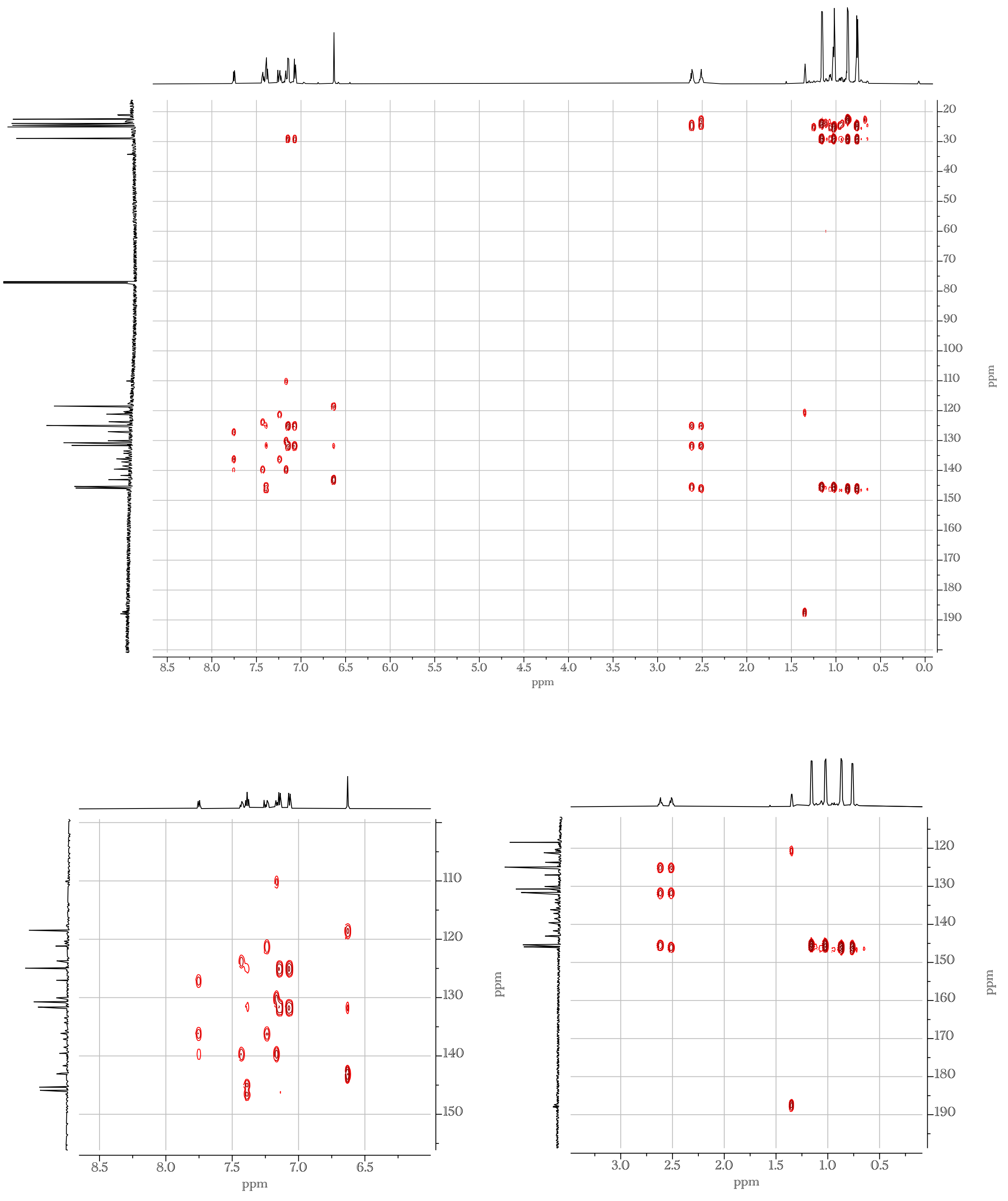

Figure S39: $^{1}H/^{13}C\{^{1}H\}$ HMBC NMR spectrum (with several zoomed perspectives) of [**3**][$B(OC_6F_5)_4$] in $CDCl_3$.

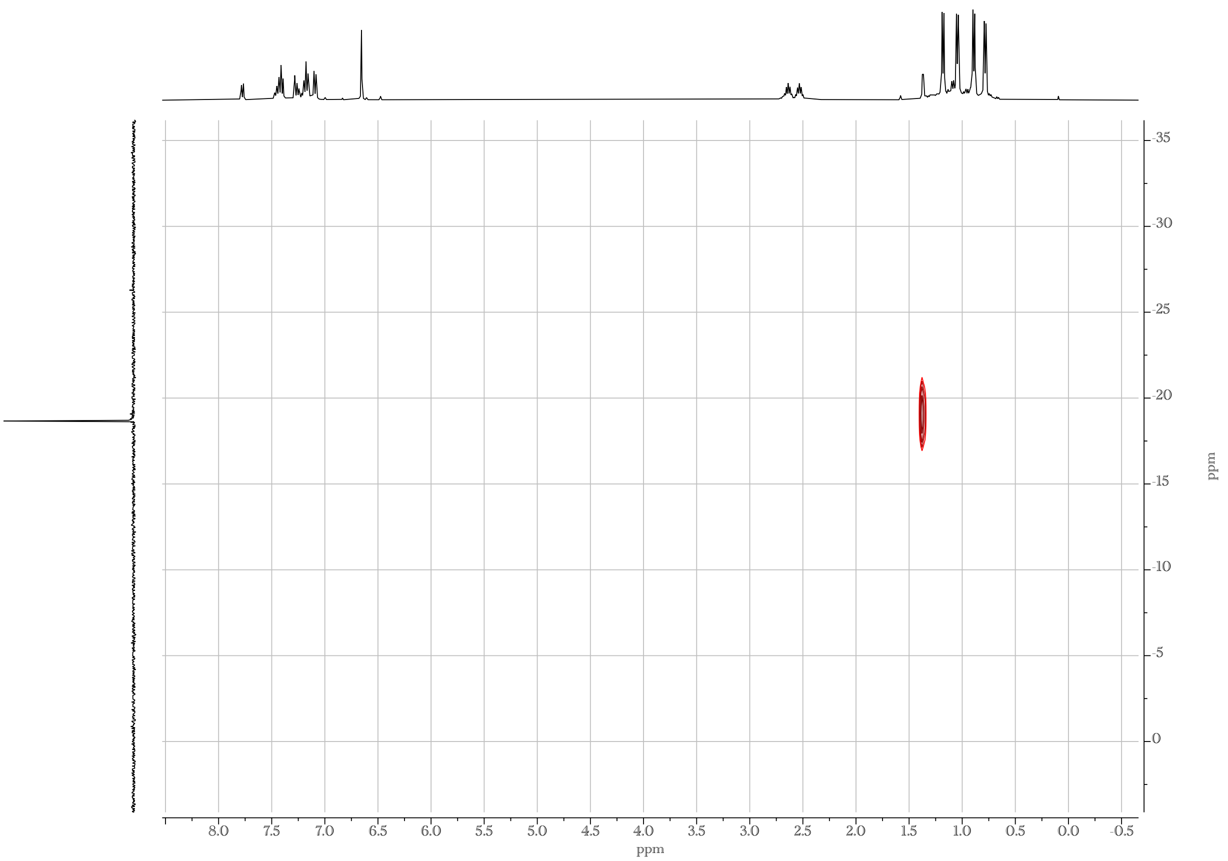

Figure S40: $^{1}H/^{31}P$ HMBC NMR spectrum of [**3**][B(OC_6F_5)$_4$] in $CDCl_3$.

2.5 Isomerization of [2][B(OC_6F_5)$_4$] to [2^{cyclo}][B(OC_6F_5)$_4$]

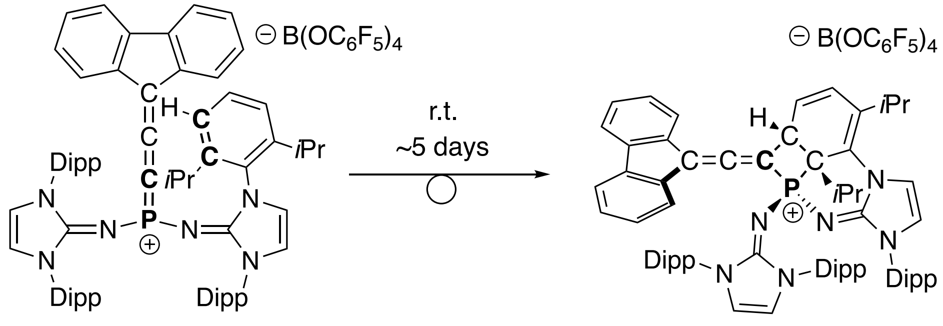

[**2**]$^+$ [**2**^{cyclo}]$^+$

A solution of [**2**][B(OC_6F_5)$_4$] in dichloromethane was left to stand in the dark for 5 days. A colour change of dark red to light red was noted. The product was obtained as a pale pink solid. The rearrangement/ isomerization reaction also occurs with the same selectively when polar halogenated solvents are used, including with trifluorotoluene, orthodifluorobenzene (1,2-DFB) and orthodichlorobenzene (1,2-DCB). Note: In THF, [**2**][B(OC_6F_5)$_4$] partially isomerizes to [**2**^{cyclo}][B(OC_6F_5)$_4$] but also decomposes to a new compound with a ^{31}P NMR resonance at 39.8 ppm which could not be identified. The decomposition in THF occurs in a ratio of roughly 2 to 1 (**[2^{cyclo}]$^+$** to unknown).

The characterized reaction was performed in CD_2Cl_2.

Note: partial assignment of the ^{13}C NMR was performed using 2D experiments.

^{1}H NMR (CD_2Cl_2, 400 MHz, 298 K) δ (ppm) = 7.80 (d, J_{HH} = 7.7, 1H, CH fluorenyl), 7.77 (d, J_{HH} = 7.7, 1H, CH fluorenyl), 7.47–7.36 (m, 2H CH fluorenyl, 1H CH Dipp: *para*), 7.32–7.22 (m, overlapped 2H CH fluorenyl, 2H CH Dipp; *para*, 2 CH Dipp; *meta*), 7.16–7.10 (m, overlapped 2H CH fluorenyl, 2H CH Dipp: *meta*), 7.01 (s, 2H, N-CH=CH-N, NIDipp), 6.90 – 6.97 (br, 2H, CH Dipp; *meta*), 6.72 (d, J_{HH} = 2.6, 1H, N-CH=CH-N, NIDipp dearomatized), 6.50 (d, J_{HH} = 2.6, 1H, N-CH=CH-N, NIDipp dearomatized), 6.05 (d, J_{HH} = 9.5 Hz, 1H, cyclohexadiene CH), 5.81 (dd, J_{HH} = 9.5 Hz, J_{HH} = 6.5 Hz, 1H, cyclohexadiene CH), 3.21 (sept, J_{HH} = 6.8 Hz, 1H, C$\underline{H}$(CH_3)$_2$), 2.82 (dd, J_{HH} = 6.5 Hz, J_{PH} = 4.6 Hz, 1H, phosphacyclobutane C$\underline{H}$), 2.80 – 2.71 (br, 1H, C$\underline{H}$(CH_3)$_2$), 2.63 (sept, J_{HH} = 6.8 Hz, 2H, C$\underline{H}$(CH_3)$_2$), 2.47–2.42 (overlapped septets, 2H, C$\underline{H}$(CH_3)$_2$), 2.26 (sept, J_{HH} = 9.5 Hz, 1H, C$\underline{H}$(CH_3)$_2$), 1.41 (m, 1H, phosphacyclobutane C-C$\underline{H}$(CH_3)$_2$), 1.30–1.22 (m, 15H, CH(C$\underline{H}_3$)$_2$), 1.12 (d, J = 6.6 Hz, 3H, CH(C$\underline{H}_3$)$_2$), 1.07 (m, 6H, CH(C$\underline{H}_3$)$_2$), 0.98 (d, J = 6.9 Hz, 3H, CH(C$\underline{H}_3$)$_2$), 0.85–0.82 (m, 9H, CH(C$\underline{H}_3$)$_2$), 0.75–0.70 (m, 6H, CH(C$\underline{H}_3$)$_2$), 0.68 (d, J_{HH} = 6.9 Hz, 3H, CH(C$\underline{H}_3$)$_2$), 0.61 (d, J_{HH} = 6.7 Hz, 3H, phosphacyclobutane C-CH(C$\underline{H}_3$)$_2$).

^{11}B NMR (CD_2Cl_2, 96 MHz, 298 K) δ (ppm) = 1.2.

^{13}C {^{1}H} NMR (CD_2Cl_2, 101 MHz, 298 K) δ (ppm) = 194.2 (d, $^2J_{CP}$ = 10 Hz, allene C2), 152.7 (C_q C=N-P of NIDipp), 146.8, 146.8, 146.7, 146.3, 146.3, 146.1 and 146.0 (C_q aromatic), 142.7 (d, $^2J_{CP}$ = 7 Hz, C_q of cyclohexadiene), 142.1 (dm, $^1J_{CF}$ = 247 Hz, CF B(OC_6F_5)$_4$: *ortho*), 139.6 (d, J_{CP} = 2 Hz), 138.9 (d, 2 Hz), 137.7 (dm, $^1J_{CF}$ = 244 Hz, CF B(OC_6F_5)$_4$: *para*), 137.6 (d, J_{CP} = 8

Hz,) 137.6 (d, J_{CP} = 8 Hz),134.9 (dm, $^1J_{CF}$ = 242 Hz, CF $B(OC_6F_5)_4$: *meta*), 133.3 (m, $B(OC_6F_5)_4$: *ipso*), 131.7, 131.5, 130.6 and 130.5 (CH aromatic), 129.6 (d, J_{CP} = 2 Hz), 129.5 (d, J_{CP} = 2 Hz), 127.9, 127.7 (d, J_{CP} = 2 Hz), 127.3 (d, J_{CP} = 2 Hz), 125.2, 125.0 (d, J_{CP} = 4 Hz, cyclohexadiene CH), 124.7, 124.6, 124.4, 123.9, 123.6 (d, J_{CP} = 2 Hz), 122.5 (d, J_{CP} = 2 Hz), 122.2 (d, J_{CP} = 11 Hz), 121.3, 121.1, 119.4 (N-$\underline{C}$H=$\underline{C}$H-N, NIDipp), 116.9 (N-$\underline{C}$H=$\underline{C}$H-N, NIDipp dearomatized), 113.9 ($^4J_{CP}$ = 2 Hz, N-$\underline{C}$H=$\underline{C}$H-N, NIDipp dearomatized), 110.5 (d, $^1J_{CP}$ = 60 Hz, allene C1), 52.5 (overlapped with CD_2Cl_2, phosphacyclobutane P-$\underline{C}$-CH(CH_3)$_2$), 47.3 ($^2J_{CP}$ = 5 Hz, phosphacyclobutane $\underline{C}$H), 34.9 (d, $^2J_{CP}$ = 4 Hz, phosphacyclobutane P-C-$\underline{C}$H(CH_3)$_2$), 29.7 ($\underline{C}$H(CH_3)$_2$), 29.3 ($\underline{C}$H(CH_3)$_2$), 29.2 ($\underline{C}$H(CH_3)$_2$), 28.9 ($\underline{C}$H(CH_3)$_2$), 28.2 ($\underline{C}$H(CH_3)$_2$), 26.1 (CH($\underline{C}H_3$)$_2$), 25.6 (CH($\underline{C}H_3$)$_2$), 25.4 (CH($\underline{C}H_3$)$_2$), 24.5 (CH($\underline{C}H_3$)$_2$), 23.4 (CH($\underline{C}H_3$)$_2$), 23.3 (CH($\underline{C}H_3$)$_2$), 23.2 (CH($\underline{C}H_3$)$_2$), 23.1 (CH($\underline{C}H_3$)$_2$), 22.9 (CH($\underline{C}H_3$)$_2$), 22.1 (CH($\underline{C}H_3$)$_2$), 21.3 (CH($\underline{C}H_3$)$_2$), 20.2, ($^3J_{CP}$ = 3 Hz, P-C-CH($\underline{C}H_3$)$_2$), 19.8 (CH($\underline{C}H_3$)$_2$), 19.8 (CH($\underline{C}H_3$)$_2$), 17.7 (d, $^3J_{CP}$ = 13 Hz, P-C-CH($\underline{C}H_3$)$_2$).

^{19}F NMR (CD_2Cl_2, 376 MHz, 298 K) δ (ppm) = -157.9 (d, $^3J_{FF}$ = 19.6 Hz, 8F, CF $B(OC_6F_5)_4$: *ortho*), -168.6 (t, $^3J_{FF}$ = 20.8 Hz, 8F, CF $B(OC_6F_5)_4$: *meta*), -172.1 (t, $^3J_{FF}$ = 22.3 Hz, 4F, CF $B(OC_6F_5)_4$: *para*).

^{31}P NMR (CD_2Cl_2, 121 MHz, 298 K) δ (ppm) = 16.3.

HR-ESI-MS: Calculated for $[C_{69}H_{80}N_6P]^+$ ([**2^{cyclo}**]$^+$) = 1023.6182, found: m/z =1023.6154, Calculated for $[B(OC_6F_5)_4]^-$ = 742.9570, found: m/z = 742.9585.

Single crystal X-ray diffraction analysis: Colourless single crystals of [**2^{cyclo}**][$B(OC_6F_5)_4$] were obtained by slow evaporation of a concentrated CH_2Cl_2 solution.

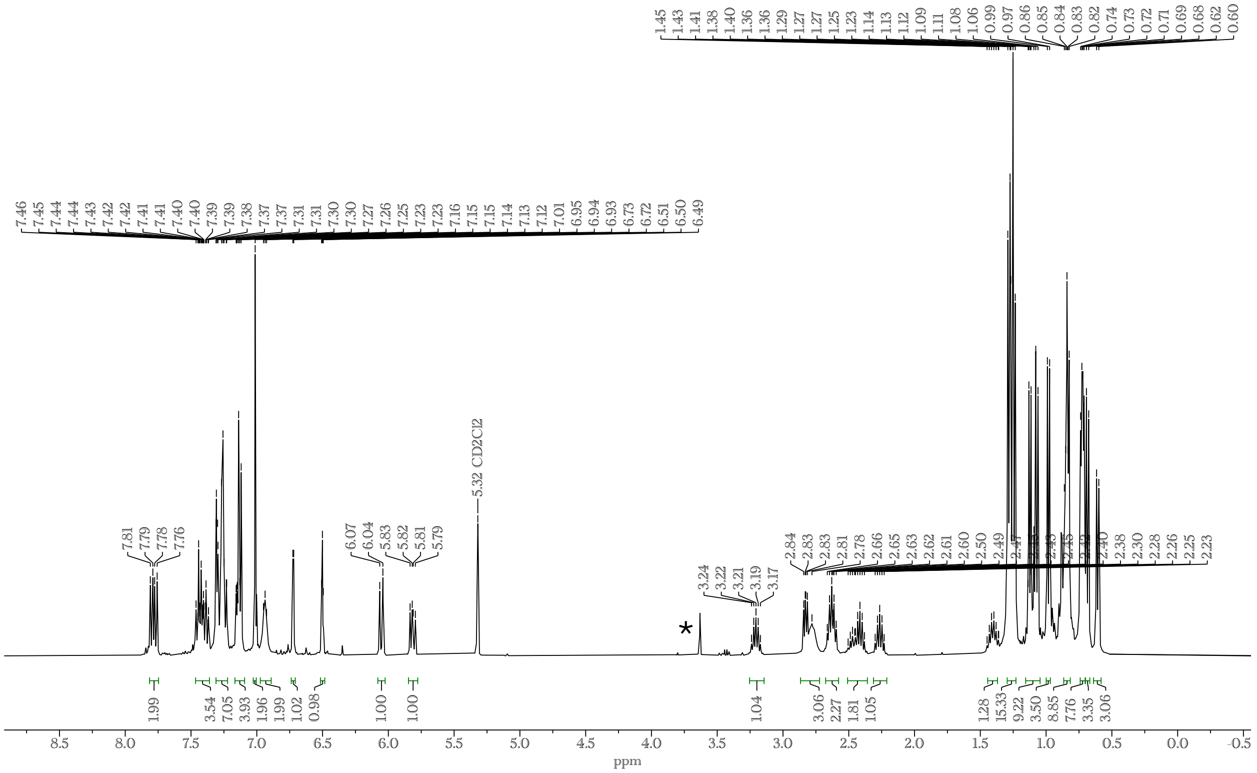

Figure S41: ^{1}H NMR (400 MHz, 298 K) spectrum of [$\mathbf{2}^{\mathbf{cyclo}}$][$B(OC_6F_5)_4$] in CD_2Cl_2. The asterisk* marks an unassigned impurity.

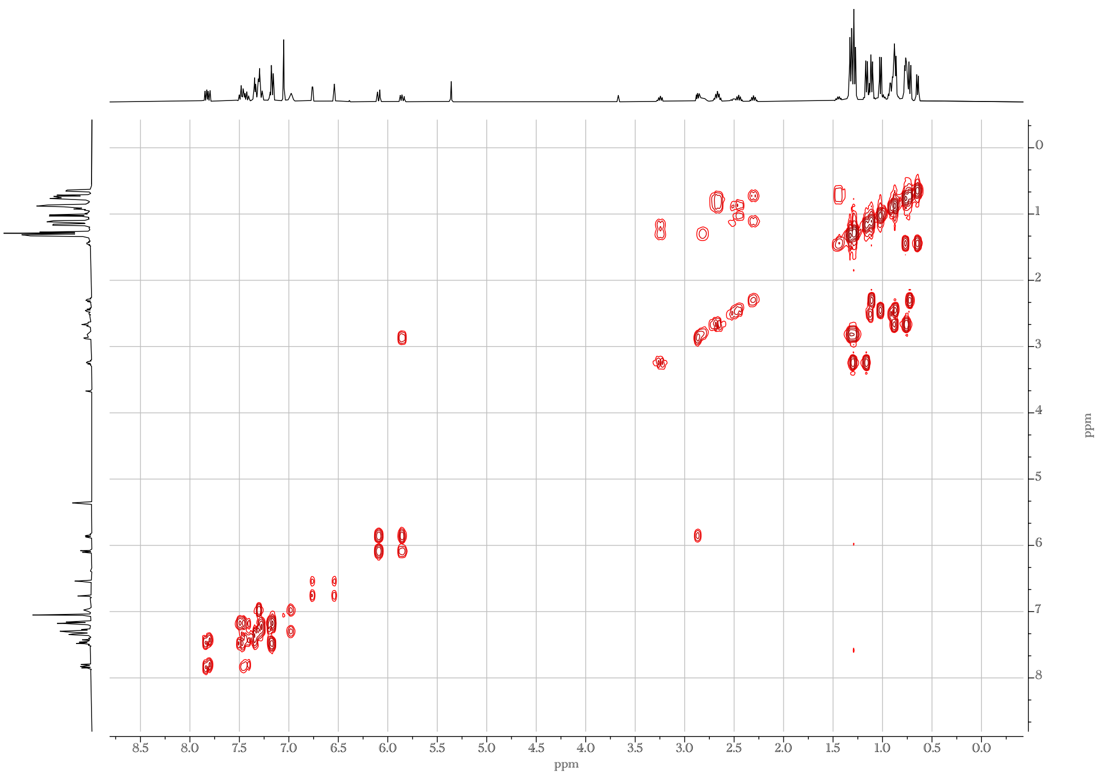

Figure S42: ^{1}H COSY NMR spectrum of [$\mathbf{2}^{\mathbf{cyclo}}$][$B(OC_6F_5)_4$] in CD_2Cl_2.

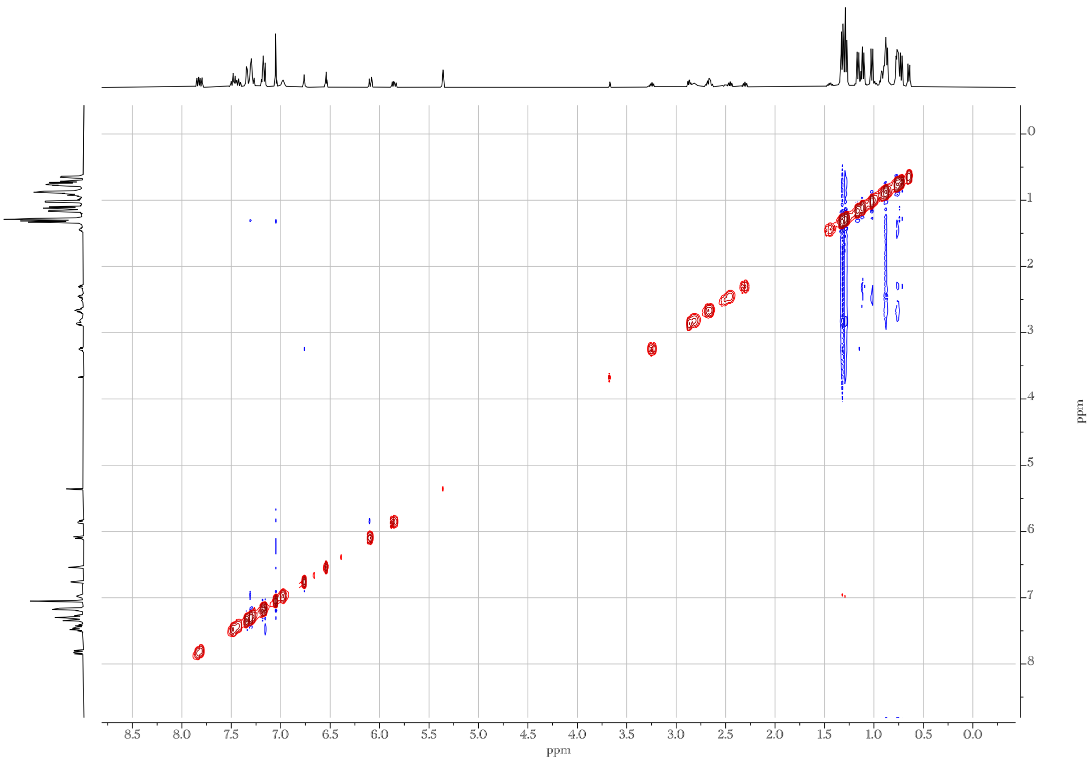

Figure S43: ^{1}H NOESY NMR spectrum of [**2**$^{\mathbf{cyclo}}$][$B(OC_6F_5)_4$] in CD_2Cl_2.

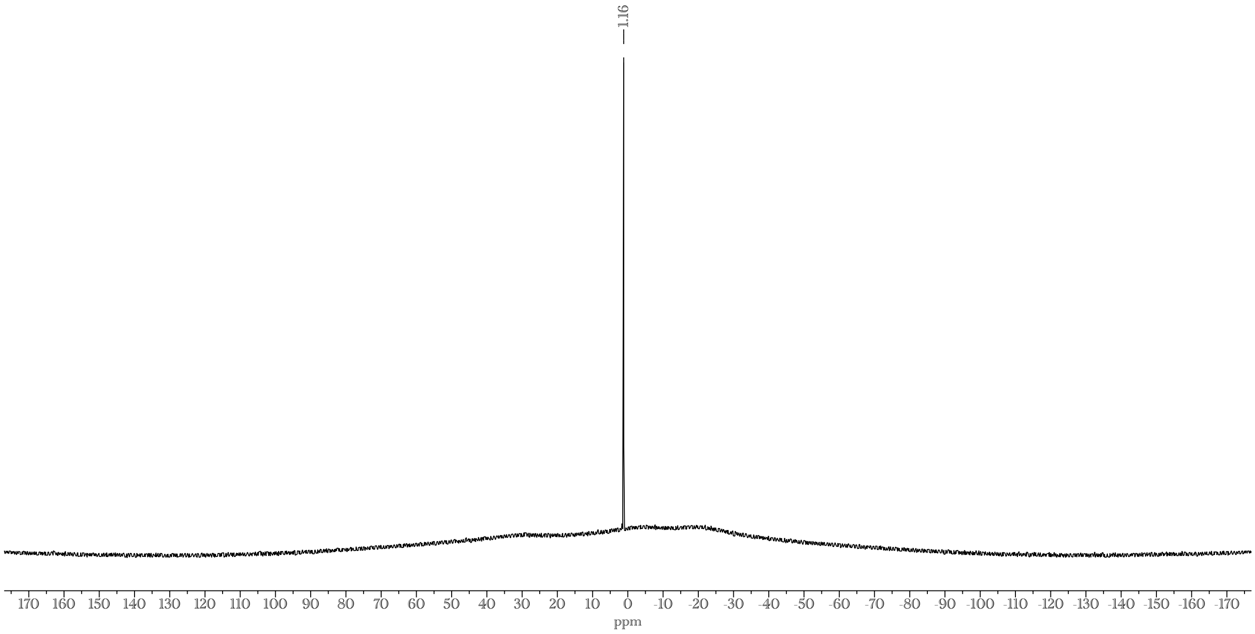

Figure S44: $^{11}B\{^1H\}$ NMR (96 MHz, 298 K) spectrum of [$\mathbf{2}^{\mathbf{cyclo}}$][$B(OC_6F_5)_4$] in CD_2Cl_2.

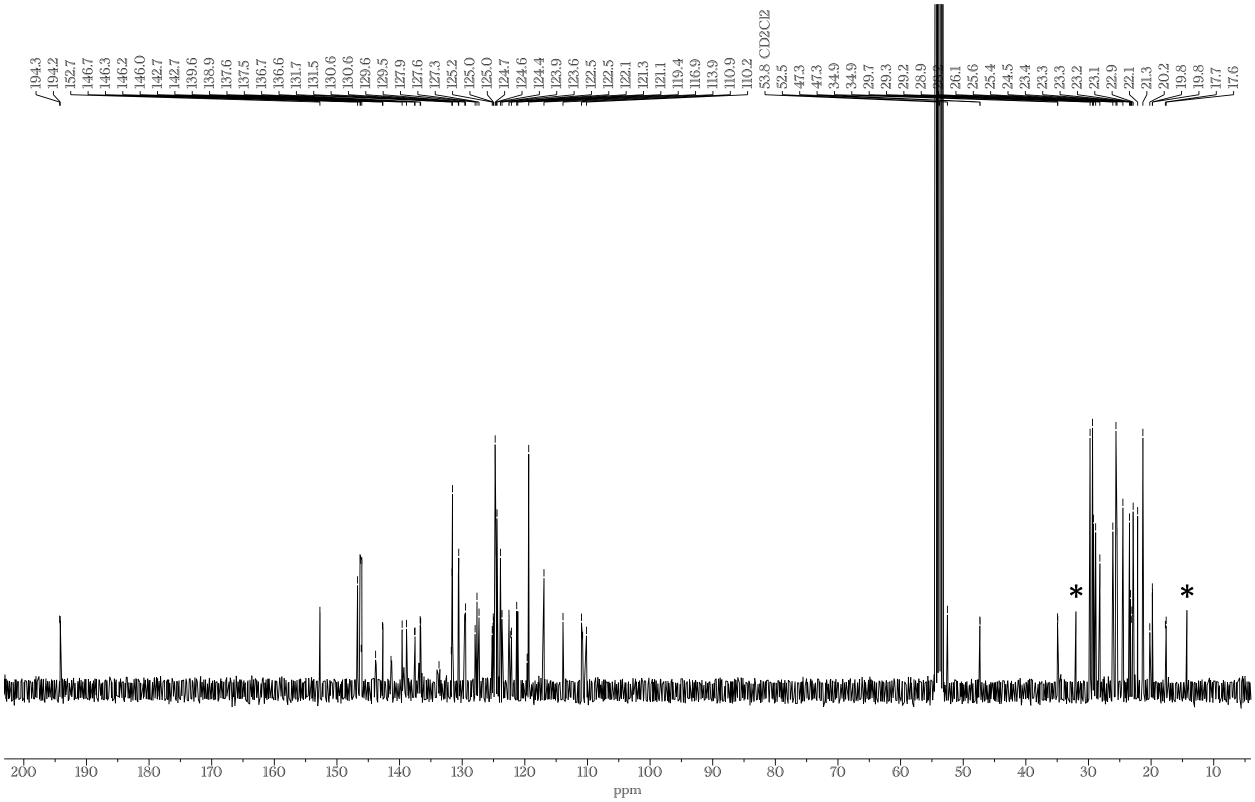

Figure S45: ^{13}C NMR (101 MHz, 298 K) spectrum of [$\mathbf{2}^{\mathbf{cyclo}}$][$B(OC_6F_5)_4$] in CD_2Cl_2. The asterisks* mark an unassigned impurity.

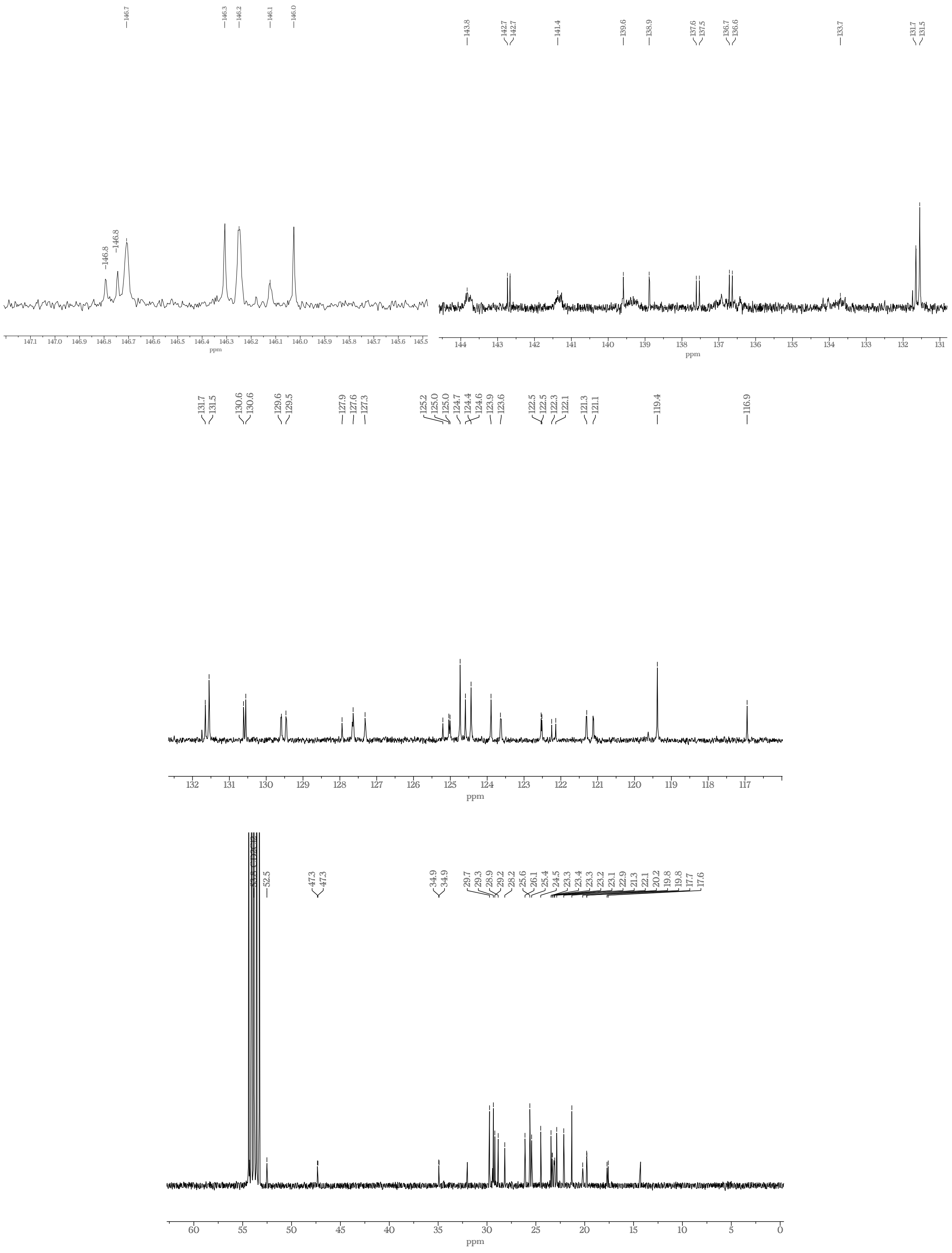

Figure S46: Enhanced zoom of ^{13}C NMR spectrum of $[\mathbf{2}^{\mathbf{cyclo}}][B(OC_6F_5)_4]$ in CD_2Cl_2.

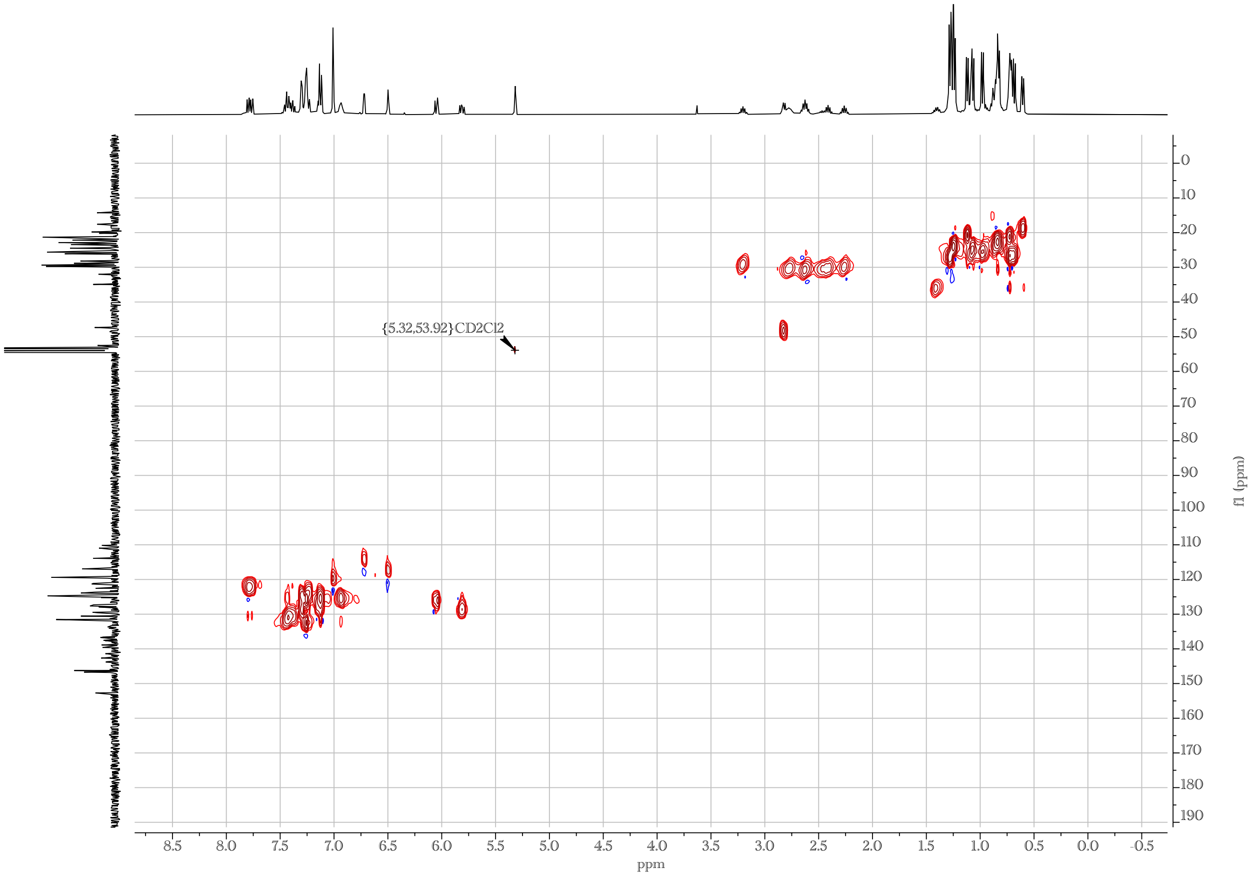

Figure S47: $^{1}H/^{13}C\{^{1}H\}$ HSQC NMR spectrum of [**2**$^{\mathbf{cyclo}}$][$B(OC_6F_5)_4$] in CD_2Cl_2.

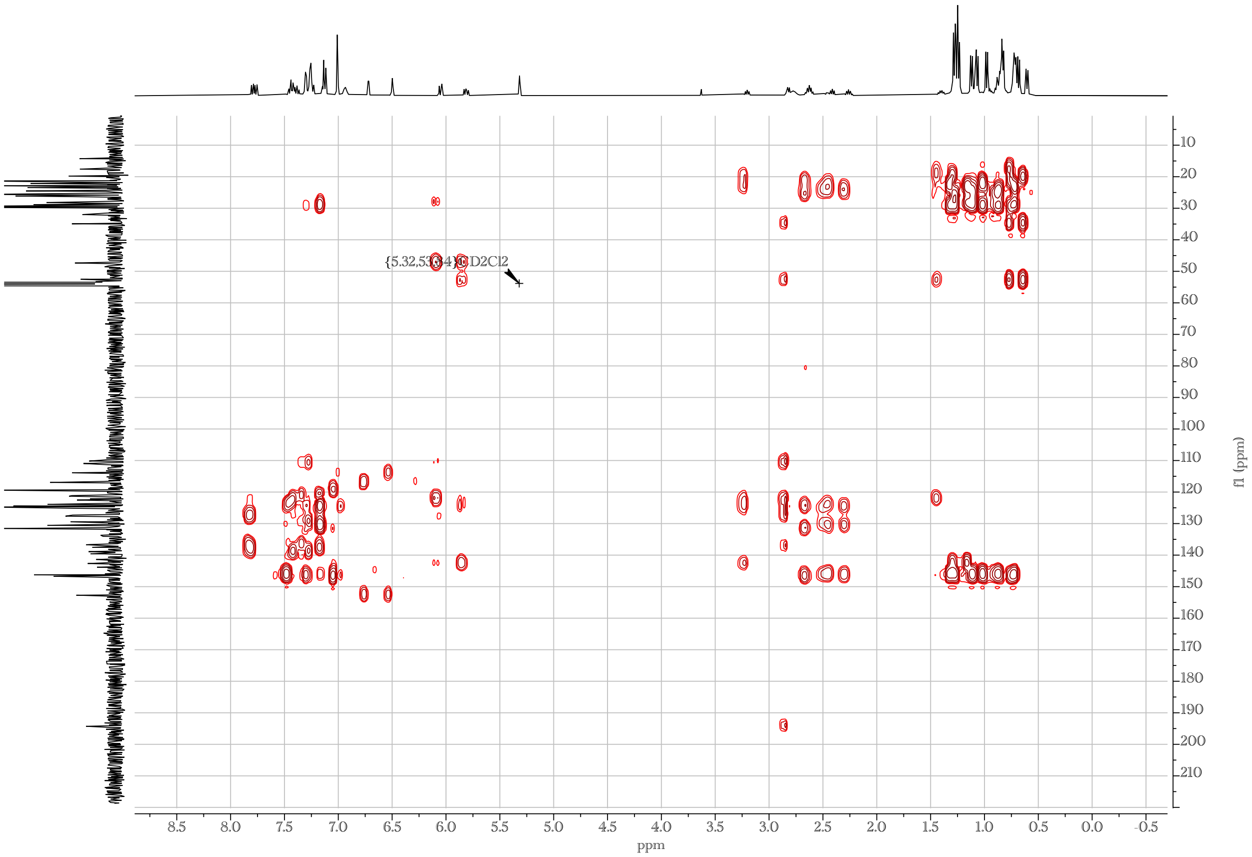

Figure S48: $^{1}H/^{13}C\{^{1}H\}$ HMBC NMR spectrum of [**2**^{cyclo}][$B(OC_6F_5)_4$] in CD_2Cl_2.

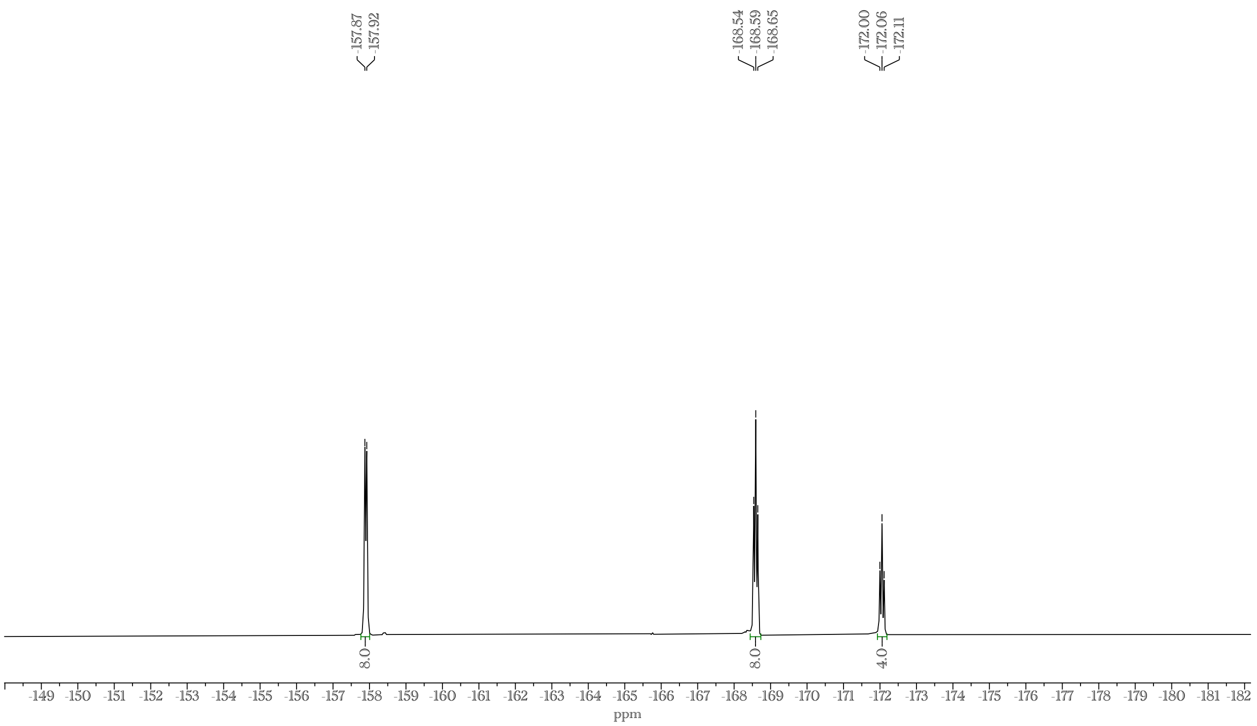

Figure S49: $^{19}F\{^{1}H\}$ NMR (376 MHz, 298 K) spectrum of [**2**$^{\mathbf{cyclo}}$][$B(OC_6F_5)_4$] in CD_2Cl_2.

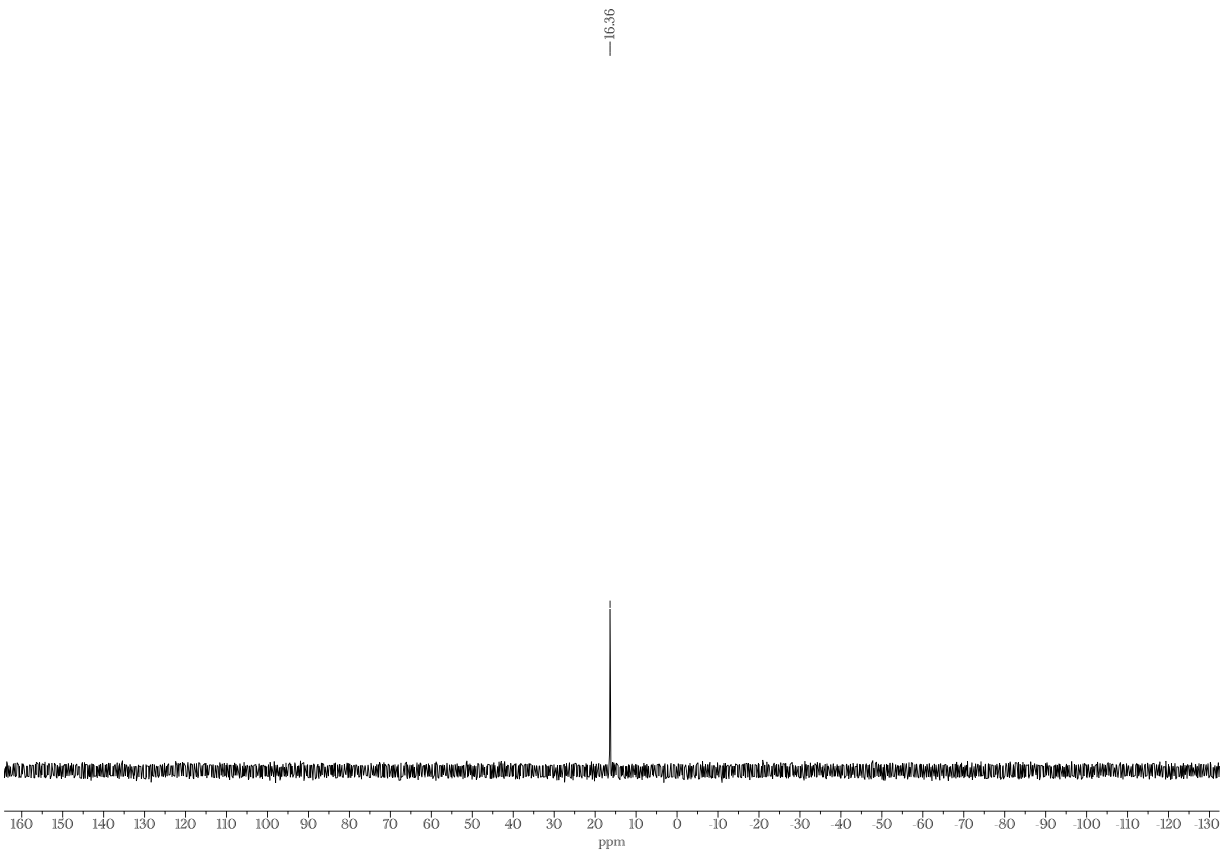

Figure S50: $^{31}P\{^{1}H\}$ NMR (162 MHz, 298 K) spectrum of [**2**$^{\mathbf{cyclo}}$][$B(OC_6F_5)_4$] in CD_2Cl_2.

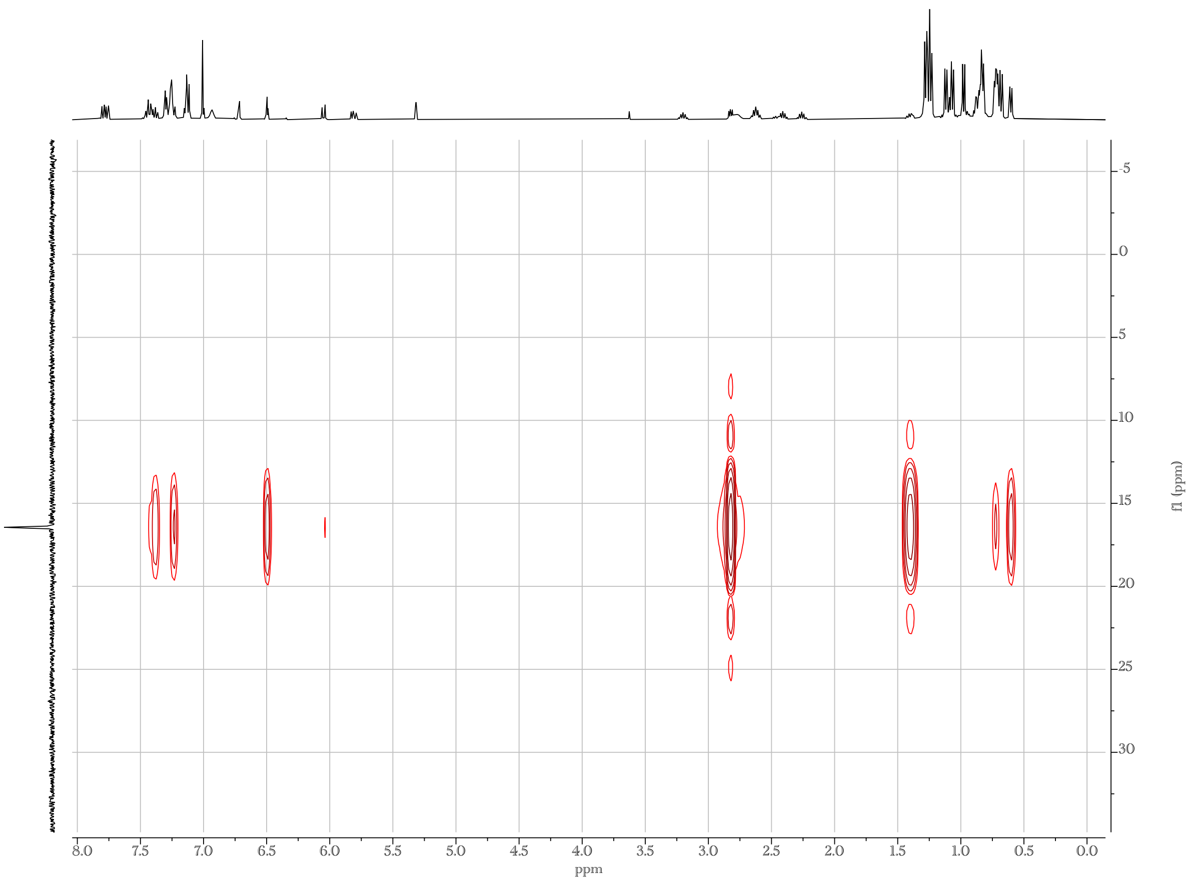

Figure S51: $^{1}H/^{31}P$ HMBC NMR spectrum of [$\mathbf{2}^{\mathbf{cyclo}}$][$B(OC_6F_5)_4$] in CD_2Cl_2.

2.6 Stability of [2^{cyclo}][$B(OC_6F_5)_4$] to ambient atmosphere

A J-Young NMR tube containing a solution of [**2**^{cyclo}][$B(OC_6F_5)_4$] in CD_2Cl_2 was opened to ambient atmosphere, and its decomposition monitored over 16 hours. The ^{1}H and ^{31}P NMR suggest minimal/ no decomposition of the phosphorus cation, while the ^{19}F NMR spectrum indicates a decomposition pathway for the borate anion.

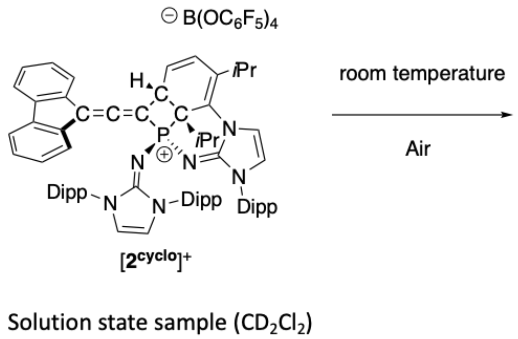

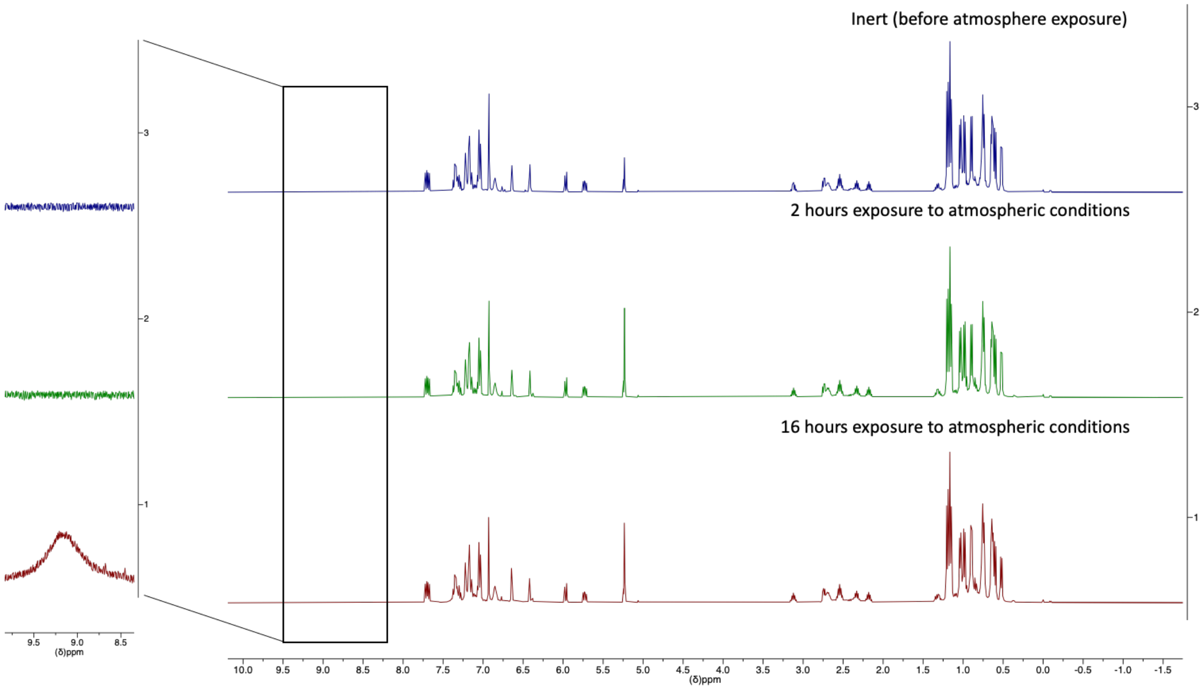

Figure S52: ^{1}H NMR spectra of a solution state sample of [**2**^{cyclo}][$B(OC_6F_5)_4$] in CD_2Cl_2 before and after exposure to air for up to 16 hours.

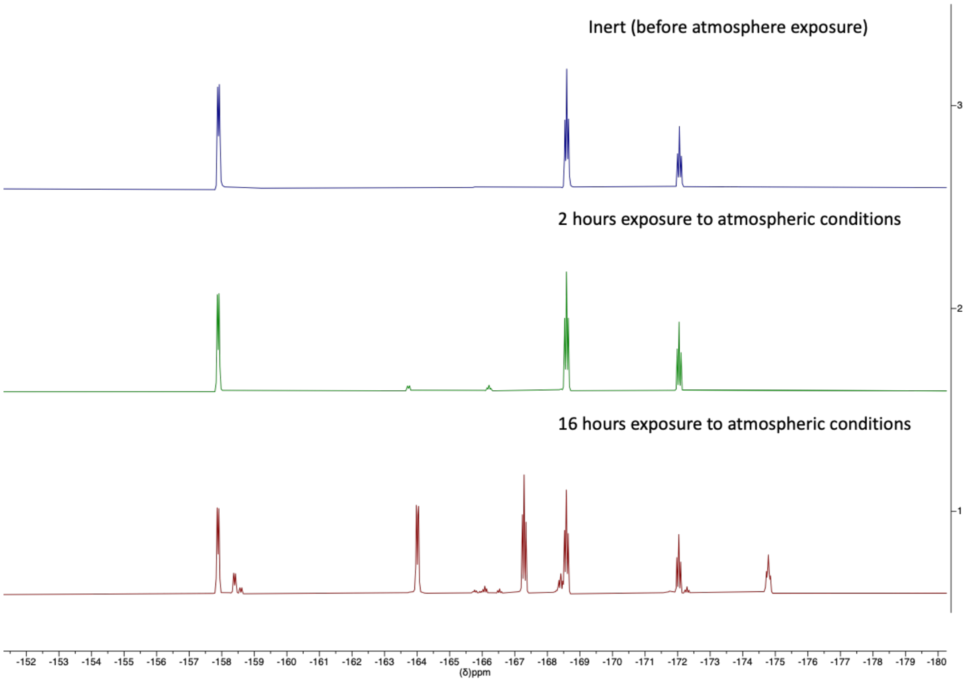

Figure S53: $^{19}F\{^1H\}$ NMR spectra of a solution state sample of [$\mathbf{2}^{\mathbf{cyclo}}$][$B(OC_6F_5)_4$] in CD_2Cl_2 before and after exposure to air for up to 16 hours.

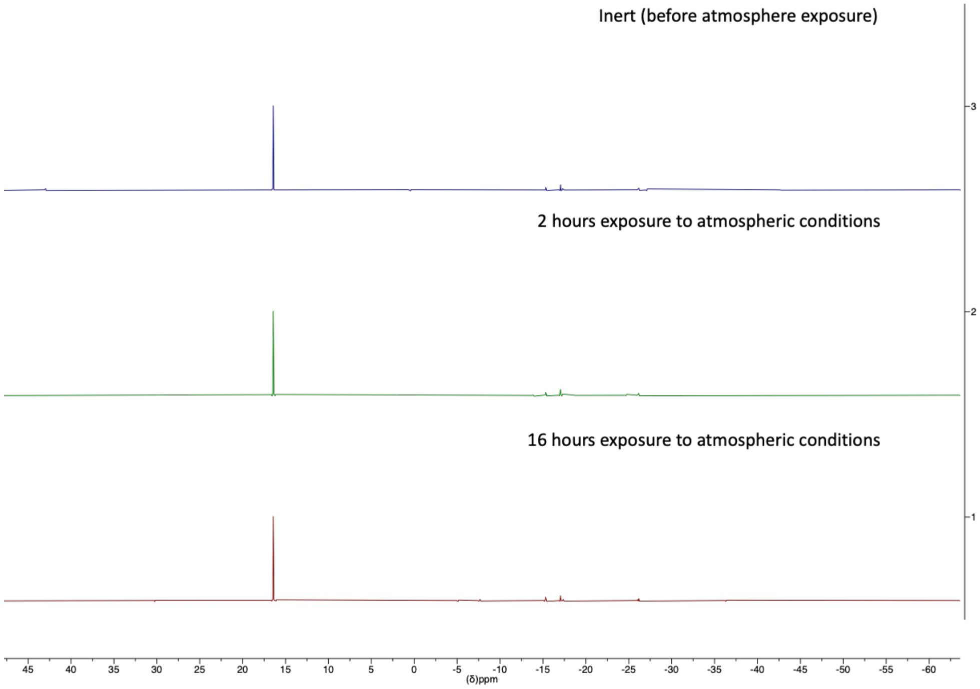

Figure S54: $^{31}P\{^1H\}$ NMR spectra of a solution state sample of [$\mathbf{2}^{\mathbf{cyclo}}$][$B(OC_6F_5)_4$] in CD_2Cl_2 before and after exposure to air for up to 16 hours.

2.7 Cycloreversion of [$\mathbf{2^{cyclo}}$]$^+$; Trapping reaction with DMAP

To a J-Young NMR tube containing a solution of [$\mathbf{2^{cyclo}}$][$B(OC_6F_5)_4$] (10 mg, 0.0057 mmol) in 1,2-DFB, 10 equivalents of DMAP (6.9 mg 0.057 mmol) were added. The reaction was monitored by heteronuclear NMR spectroscopy, beginning with a heating period of 40 °C, then increasing the temperature in 10-degree increments. Complete cycloreversion occurs at ~60 °C over a total time period of approximately 16 hours. Once conversion was complete, the NMR sample was reduced to a solid residue and directly reconstituted in deuterated acetonitrile.

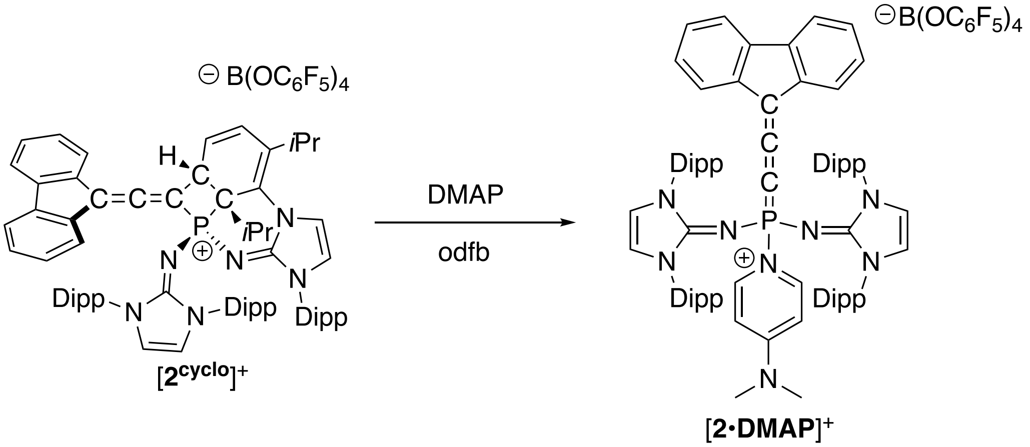

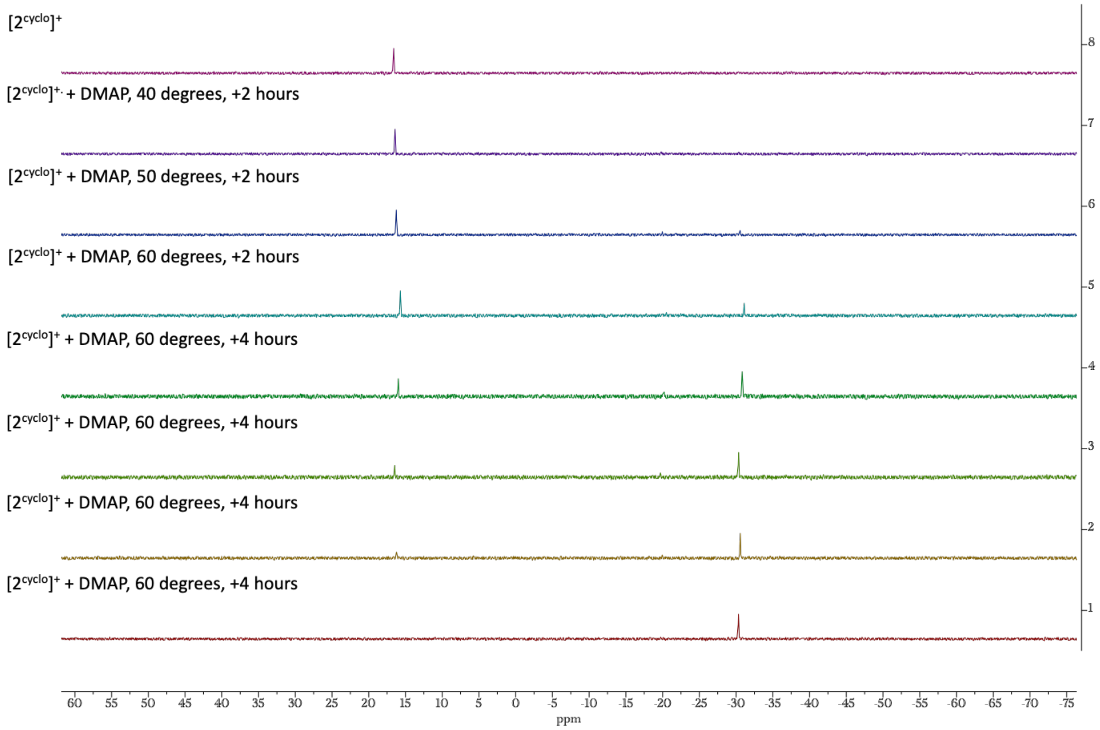

Figure S55: Stacked $^{31}P\{^1H\}$ NMR spectra monitoring the conversion of [$\mathbf{2^{cyclo}}$][$B(OC_6F_5)_4$] to [$\mathbf{2^{cyclo}}$][$B(OC_6F_5)_4$] in 1,2-DFB.

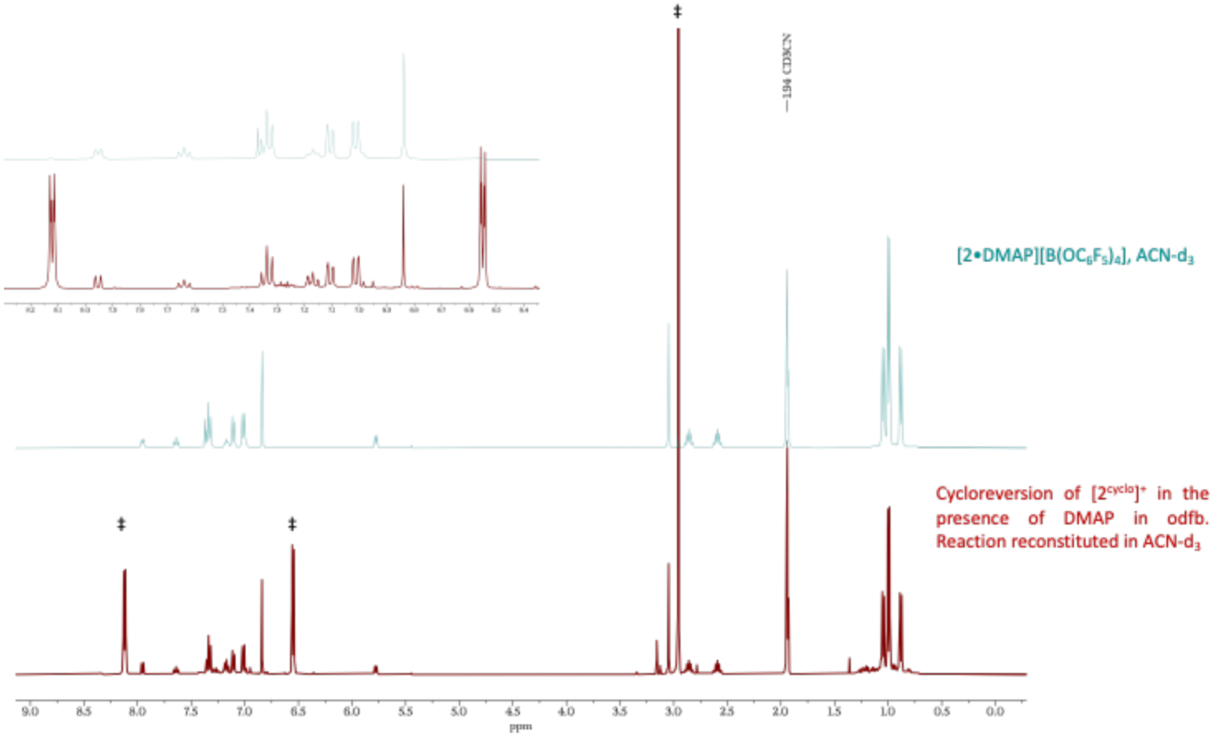

Figure S56: Stacked ^{1}H NMR spectra, the top trace corresponds to an isolated sample of [**2**^{cyclo}][B(OC_6F_5)$_4$] in ACN-d_3, the bottom trace corresponds to experiment 1.3. Following the reaction of [**2**^{cyclo}][B(OC_6F_5)$_4$] with DMAP, 1,2-DFB was removed under reduced pressure and the residue was directly dissolved in acetonitrile-d_3. The ‡'s reflects the ^{1}H NMR resonances of excess DMAP.

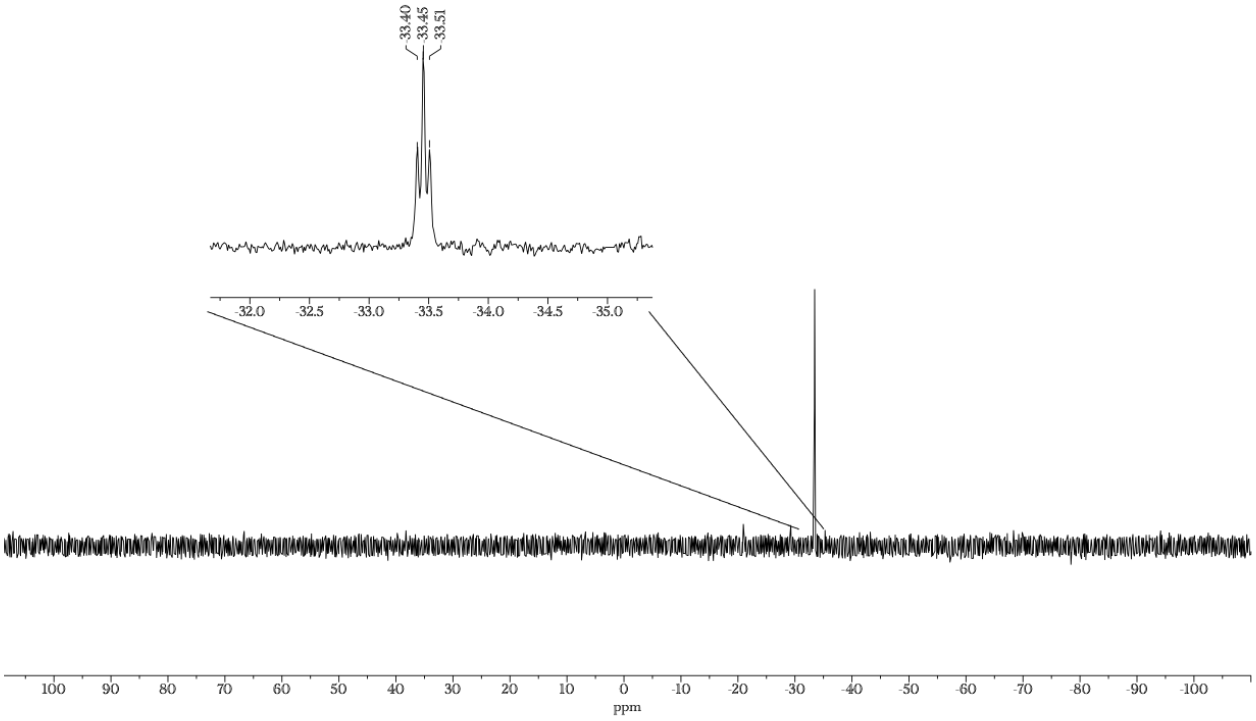

Figure S57: ^{31}P NMR spectra following the reaction of [$\mathbf{2}^{\mathbf{cyclo}}$][$B(OC_6F_5)_4$] with DMAP, where 1,2-DFB was removed under reduced pressure and the residue was directly dissolved in acetonitrile-d_3.

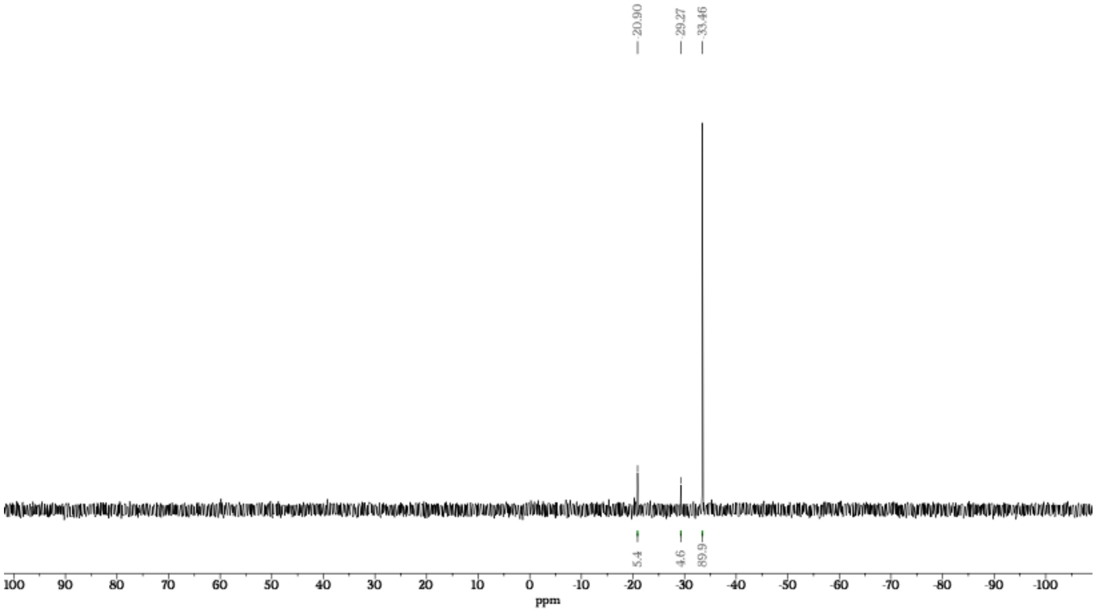

Figure S58: $^{31}P\{^1H\}$ NMR spectra following the reaction of [$\mathbf{2}^{\mathbf{cyclo}}$][$B(OC_6F_5)_4$] with DMAP, where 1,2-DFB was removed under reduced pressure and the residue was directly dissolved in acetonitrile-d_3.

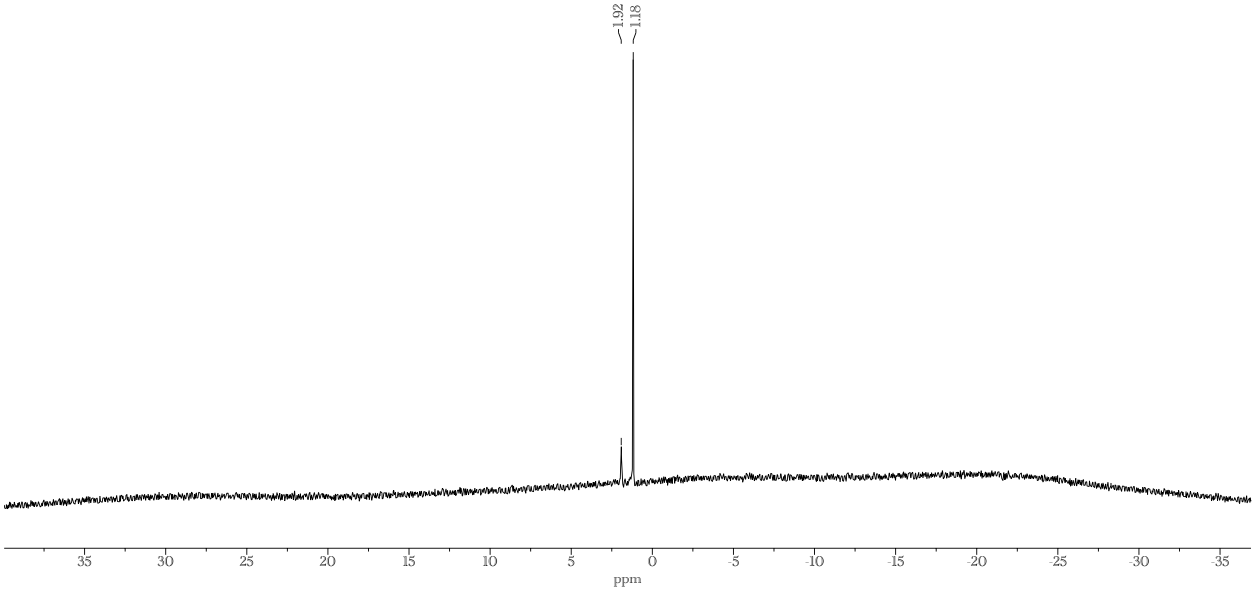

Figure S59: $^{11}B\{^{1}H\}$ NMR following the reaction of [$\mathbf{2}^{\mathbf{cyclo}}$][$B(OC_6F_5)_4$] with DMAP, where 1,2-DFB was removed under reduced pressure and the residue was directly dissolved in acetonitrile-d_3.

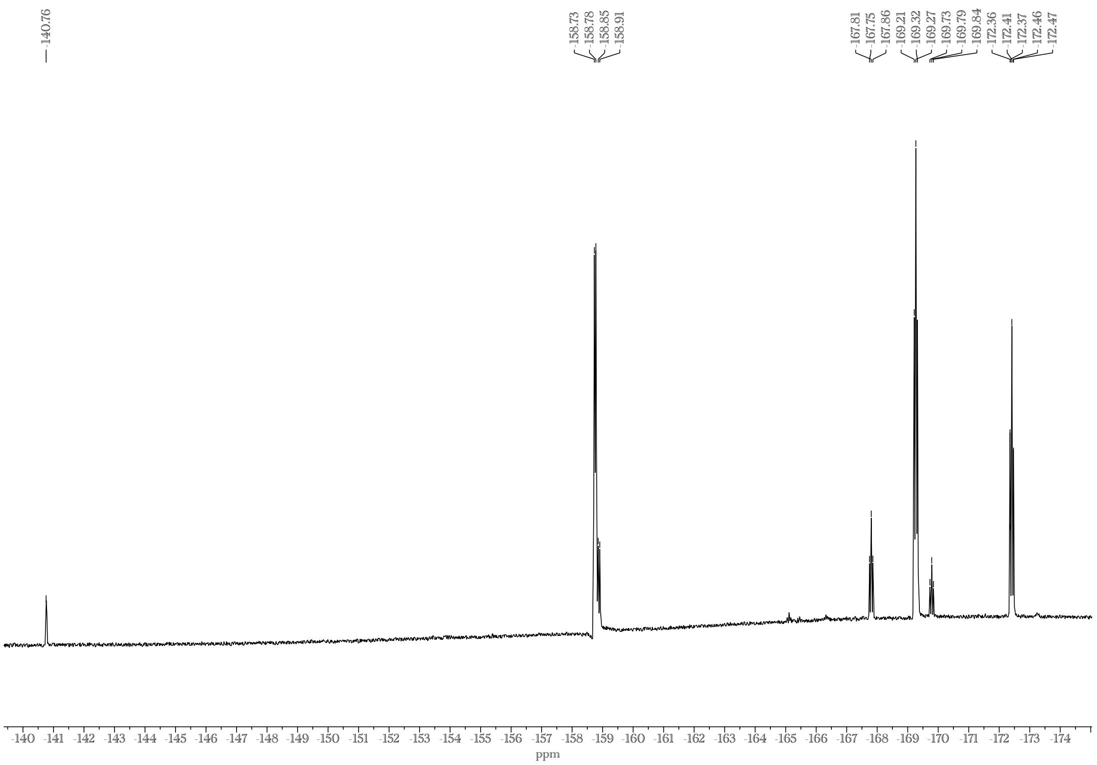

Figure S60: $^{19}F\{^{1}H\}$ NMR following the reaction of [$\mathbf{2}^{\mathbf{cyclo}}$][$B(OC_6F_5)_4$] with DMAP, where 1,2-DFB was removed under reduced pressure and the residue was directly dissolved in acetonitrile-d_3.

2.8 Attempted Determination of Gutmann Beckett Acceptor number for [2][B(OC$_6$F$_5$)$_4$]

Experiments for the determination of a Gutmann Beckett Acceptor Number[43] (AN) was attempted by a modified procedure.[44,45] A cooled (-40 °C) CD_2Cl_2 solution of [**2**][B(OC$_6$F$_5$)$_4$] (0.019 mmol) was combined with a cooled (-40 °C) CD_2Cl_2 solution of excess Et_3PO (TEPO) (2 equiv) and then allowed to gradually warm to room temperature. The solutions were precooled to minimize [**2**]$^+$ to [**2**^{cyclo}]$^+$ conversion. The mixture was prepared in a J-Young tube and monitored by NMR approximately two hours after combining.

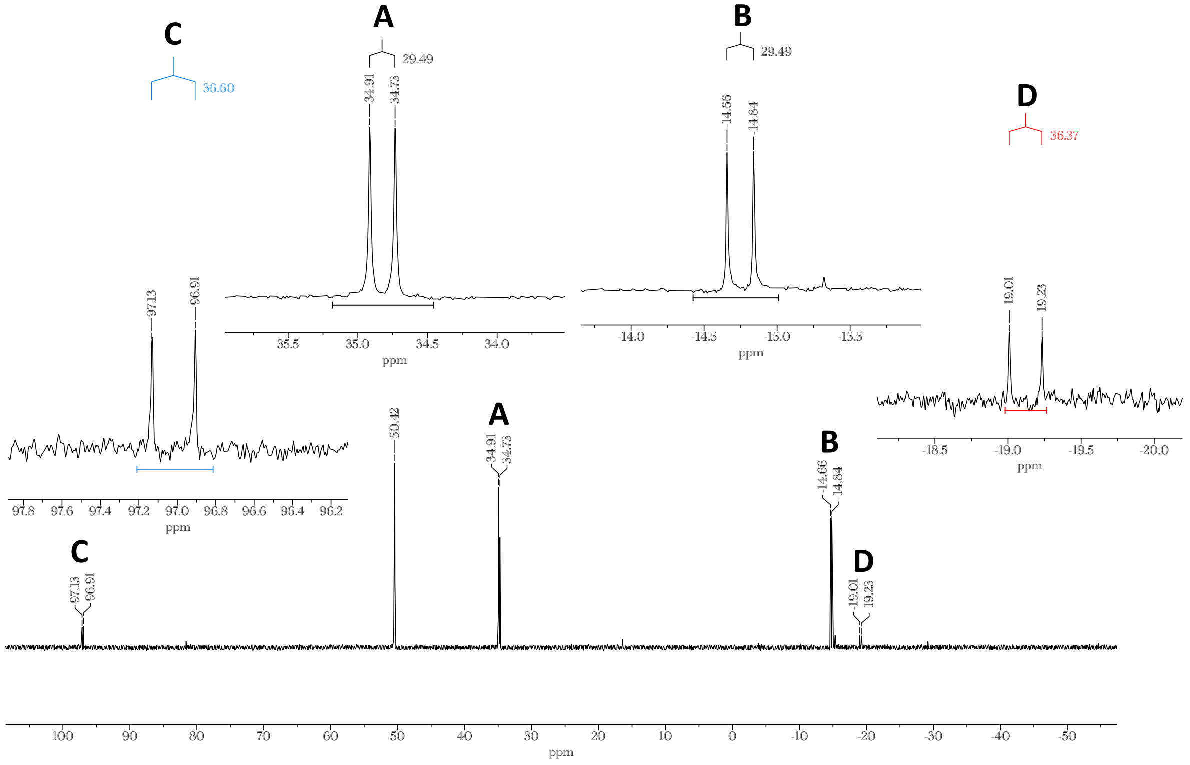

Figure S61: $^{31}P\{^1H\}$ NMR spectrum following the addition of TEPO to [**2**][B(OC$_6$F$_5$)$_4$] in CD_2Cl_2.

The reaction gives rise primarily to a set of doublet resonances (δ (ppm) = +34.8 **A** and δ = −14.6 **B**) in the $^{31}P\{^1H\}$ NMR spectrum which exhibit a J_{PP} coupling constant of 29 Hz. Excess TEPO can also be seen in the reaction mixture (50.4 ppm). The principles of the Gutmann Beckett method imply that the Lewis Adduct of TEPO and [**2**]$^+$ would be expected to give rise to atleast one ^{31}P NMR resonance which is downfield of TEPO. This is due to deshielding of the ^{31}P nuclei of the TEPO probe upon Lewis acid coordination. Employing the acceptor number formula, the resonances belonging to the ^{31}P resonances **A** and **B** in the reaction mixture would both give negative acceptor numbers (AN = –0.14 and –14.6), suggesting that the product is unlikely a Lewis acid base adduct of [**2**]$^+$ and TEPO. Minimal amounts of a second product are observed in the baseline of the $^{31}P\{^1H\}$ NMR spectrum (δ (ppm) = +97.0 **C** and –19.1**D**) which exhibit a J_{PP} coupling constant of 36 Hz.

$$AN = \frac{\delta_{interacting\ TEPO} - 41.0}{86.14 - 41.0}\ x\ 100$$

2.9 Hydrolysis study of [2][B(OC$_6$F$_5$)$_4$] to [**2•H$_2$O**][B(OC$_6$F$_5$)$_4$].

A red single crystal of [**2**][B(OC$_6$F$_5$)$_4$] was exposed to ambient atmosphere conditions at room temperature while suspended in paratone oil. After ~5 minutes, a gradual colour change was observed, from red to yellow. Once the crystal had completely transformed in colour, it was remounted on the diffractometer, providing insight into connectivity of the hydrolysis product [**2•H$_2$O**][B(OC$_6$F$_5$)$_4$].

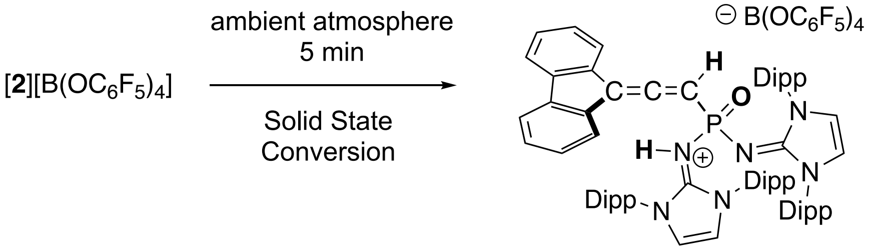

[**2·H$_2$O**][B(OC$_6$F$_5$)$_4$]

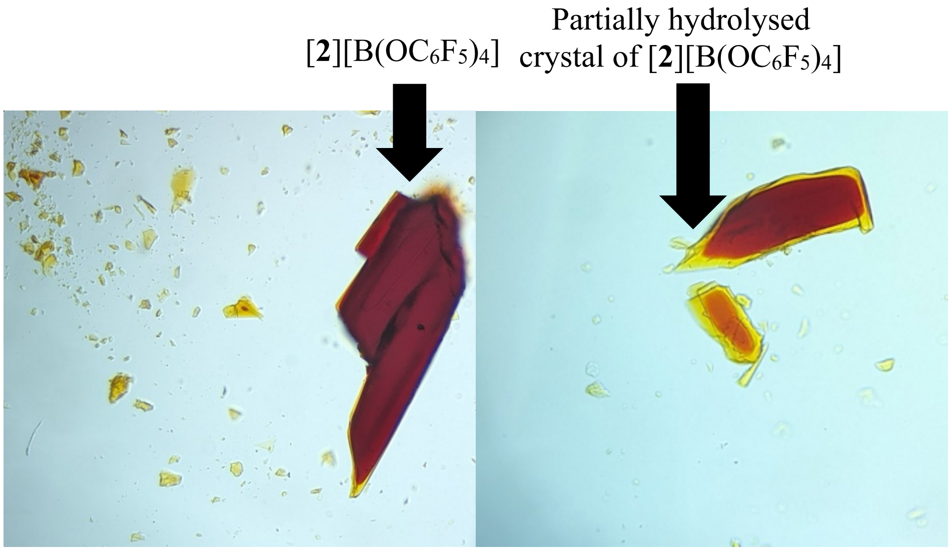

Figure S62: Pictures observing the solid state conversion of [**2**][B(OC$_6$F$_5$)$_4$] to [**2•H$_2$O**][B(OC$_6$F$_5$)$_4$]**.**

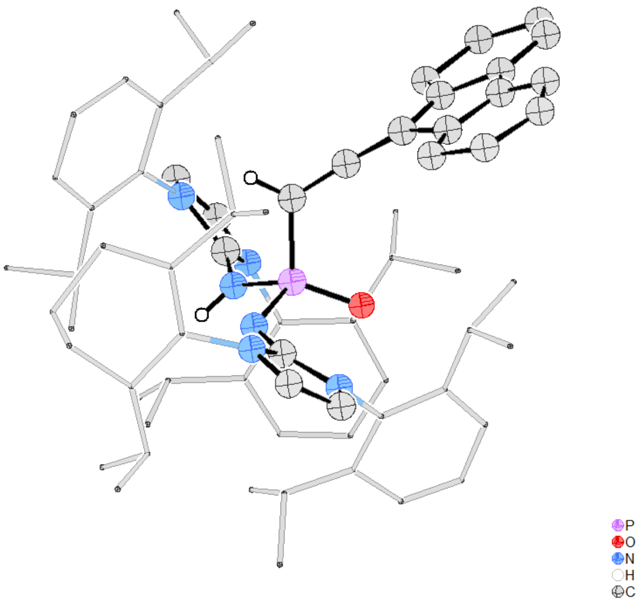

Figure S63: Solid state molecular atom arrangement of [**2•H_2O**]$^+$. Dipp groups are shown in wireframe and the anion is excluded for clarity. Atoms are shown in ball and stick.

NMR Characterization of [**2•H$_2$O**][B(OC$_6$F$_5$)$_4$].

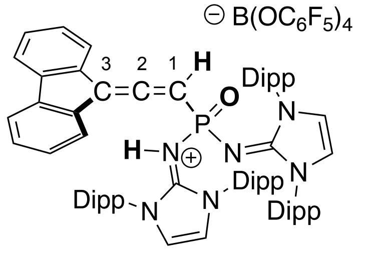

A single drop of water was added via syringe to a suspension of [**2**][B(OC$_6$F$_5$)$_4$] (~10 mg) in pentane which was stored under argon. The flask was shaken and periodically sonicated until the vibrant red colour of [**2**]$^+$ completely subsided (took approximately 1 hour). Removal of the solvent and excess water afforded a beige powder which was directly dissolved in CD$_2$Cl$_2$ for NMR analysis.

^{1}H NMR (CD$_2$Cl$_2$, 400 MHz, 298 K) δ (ppm) = 7.79 (d, J_{HH} = 7.6, 2H, CH fluorenyl), 7.55 −7.45 (br, 4H, CH Dipp; *para*), 7.41 (t, J_{HH} = 7.6, 2H, CH fluorenyl), 7.28 (t, J_{HH} = 7.6, 2H, CH fluorenyl) 7.35 −7.15 (br, 8H, CH Dipp; *meta*), 7.10 (t, J_{HH} = 7.6, 2H, CH fluorenyl), 7.05−6.80 (br, 4H, N-C<u>H</u>=C<u>H</u>-N), 5.40 (broad s, 1H, allene CH), 2.65−2.35 (broad m, 8H, C<u>H</u>(CH$_3$)$_2$), 1.15−1.10 (broad doublet, 12H, CH(C<u>H</u>$_3$)$_2$), 1.05−0.90 (br, 24H, CH(C<u>H</u>$_3$)$_2$), 0.88−0.80 (broad doublet, 12H, CH(C<u>H</u>$_3$)$_2$).

Note: N-H proton is fluxional between the two exocyclic imine nitrogen atoms and cannot be detected at room temperature. At lower temperatures, the N-H proton appears as a broad singlet within the range 4.5-4.7 ppm. The fluctuation causes broadening of all the NHI related protons.

^{11}B NMR (CD$_2$Cl$_2$, 96 MHz, 296 K) δ (ppm) = 1.6.

^{13}C NMR (CD$_2$Cl$_2$, 176 MHz, 298 K) δ (ppm) = 207.4 (s, P-HC=<u>C</u>=CR$_2$, position 2), 146.7 (C$_q$ Dipp: *ortho*), 142.6 (dm, $^{1}J_{CF}$ = 245 Hz, CF B(OC$_6$F$_5$)$_4$: *ortho*), 139.0 (CH fluorenyl), 138.2 (dm, $^{1}J_{CF}$ = 246 Hz, CF B(OC$_6$F$_5$)$_4$: *para*), 136.9 (d, $^{4}J_{PC}$ = 7 Hz, CH fluorenyl),135.2 (dm, $^{1}J_{CF}$ = 241 Hz, CF B(OC$_6$F$_5$)$_4$: *meta*), 133.7 (m, CF B(OC$_6$F$_5$)$_4$: *ipso*), 129.1 (CH Dipp: *para*), 127.2, (CH fluorenyl), 126.0−124.5 (br, CH, Dipp; *meta*),123.8 (br, CH fluorenyl), 121.0, (CH fluorenyl), 107.7 (d, $^{3}J_{CP}$ = 16 Hz, position 3), 96.5 (d, $^{1}J_{CP}$ = 160 Hz, P-H<u>C</u>=C=CR$_2$, position 1), 29.5 (br, (<u>C</u>H(CH$_3$)$_2$), 25.3 (CH(<u>C</u>H$_3$)$_2$), 23.0 (br, CH(<u>C</u>H$_3$)$_2$)

Note: N-H proton fluctuation broadens the carbon resonances of the NHI substituents. The quaternary carbons including the Dipp *ipso* carbon, the central guanidine carbon (N$_2$<u>C</u>=N) and the NHI backbone carbons (N-<u>C</u>H=<u>C</u>H-N) are too broad to be definitively assigned.

^{19}F NMR (CD$_2$Cl$_2$, 282 MHz, 296 K) δ (ppm) = -157.9 (d, $^{3}J_{FF}$ = 19.8 Hz, 8F, CF B(OC$_6$F$_5$)$_4$: *ortho*), -168.6 (m, 8F, CF B(OC$_6$F$_5$)$_4$: *meta*), -172.0 (m, 4F, CF B(OC$_6$F$_5$)$_4$: *para*).

^{31}P NMR (CD$_2$Cl$_2$, 121 MHz, 296 K) δ (ppm) = -15.3.

HR-ESI-MS: Calculated for [C$_{69}$H$_{82}$N$_6$OP]$^+$ ([**2•H$_2$O**]$^+$) *m/z* = 1041.6286, found: *m/z* =1041.6288

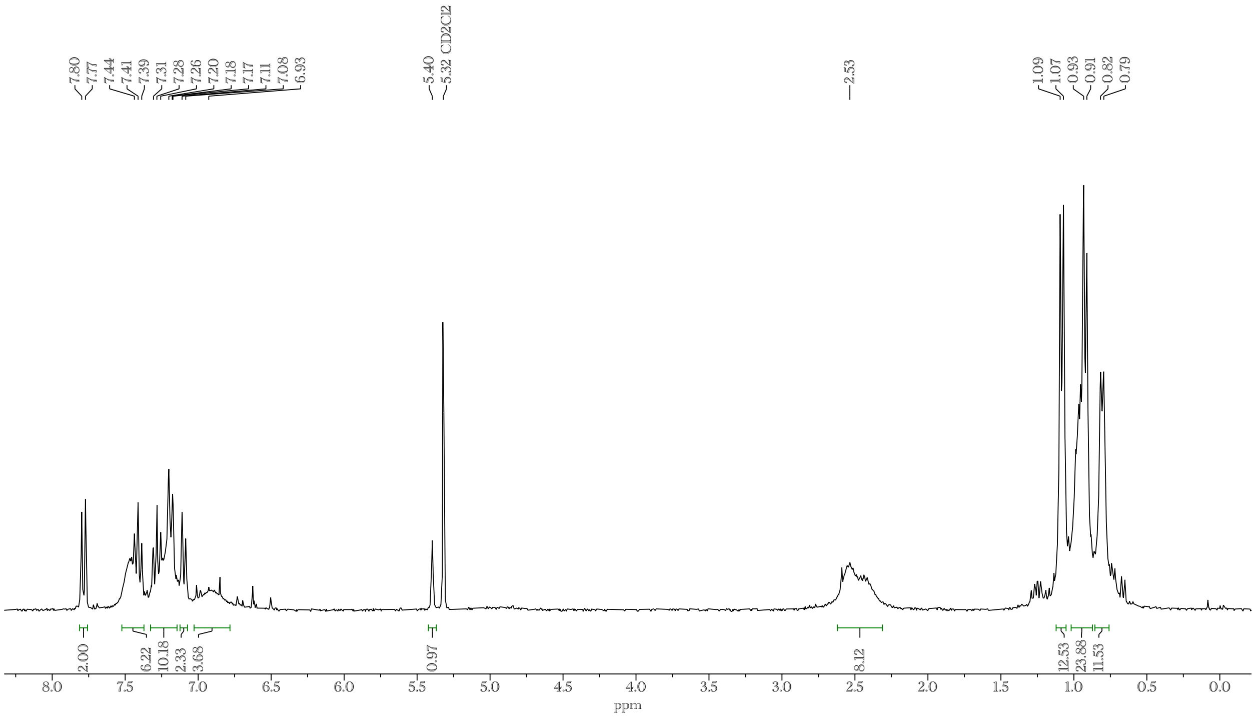

Figure S64: ^{1}H NMR (300 MHz, 298 K) spectrum of [**2•H$_2$O**][B(OC$_6$F$_5$)$_4$] in CD$_2$Cl$_2$.

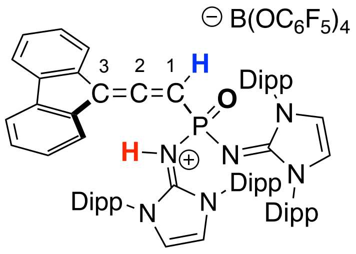

[**2•H₂O**][B(OC₆F₅)₄]

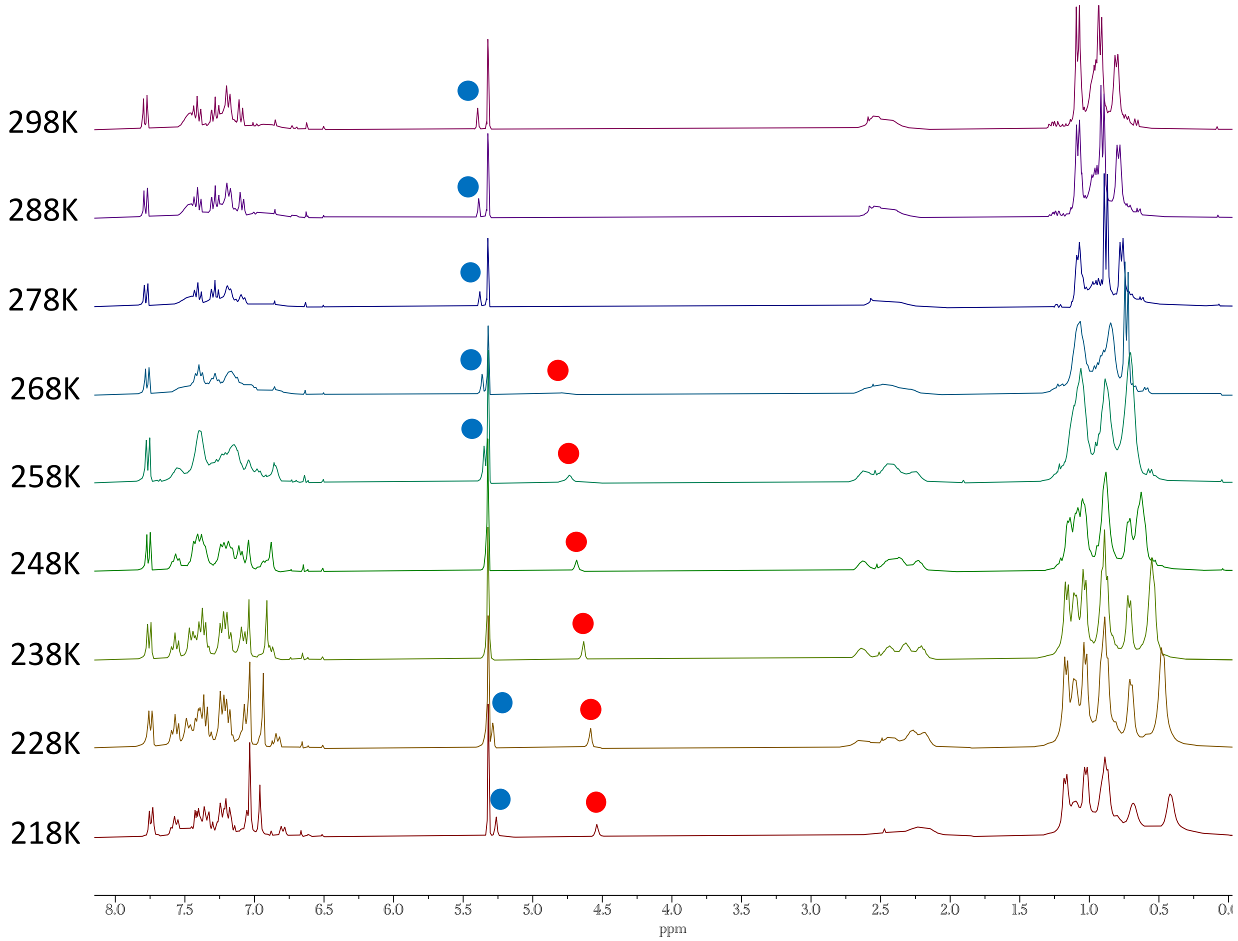

Figure S65: Stacked ^{1}H NMR (300 MHz) spectra of [**2•H₂O**][B(OC₆F₅)₄] acquired at different temperatures in CD_2Cl_2. Note that the allenic proton peak designated by the blue circle overlaps with the residual solvent for the acquisitions at 248K and 238K.

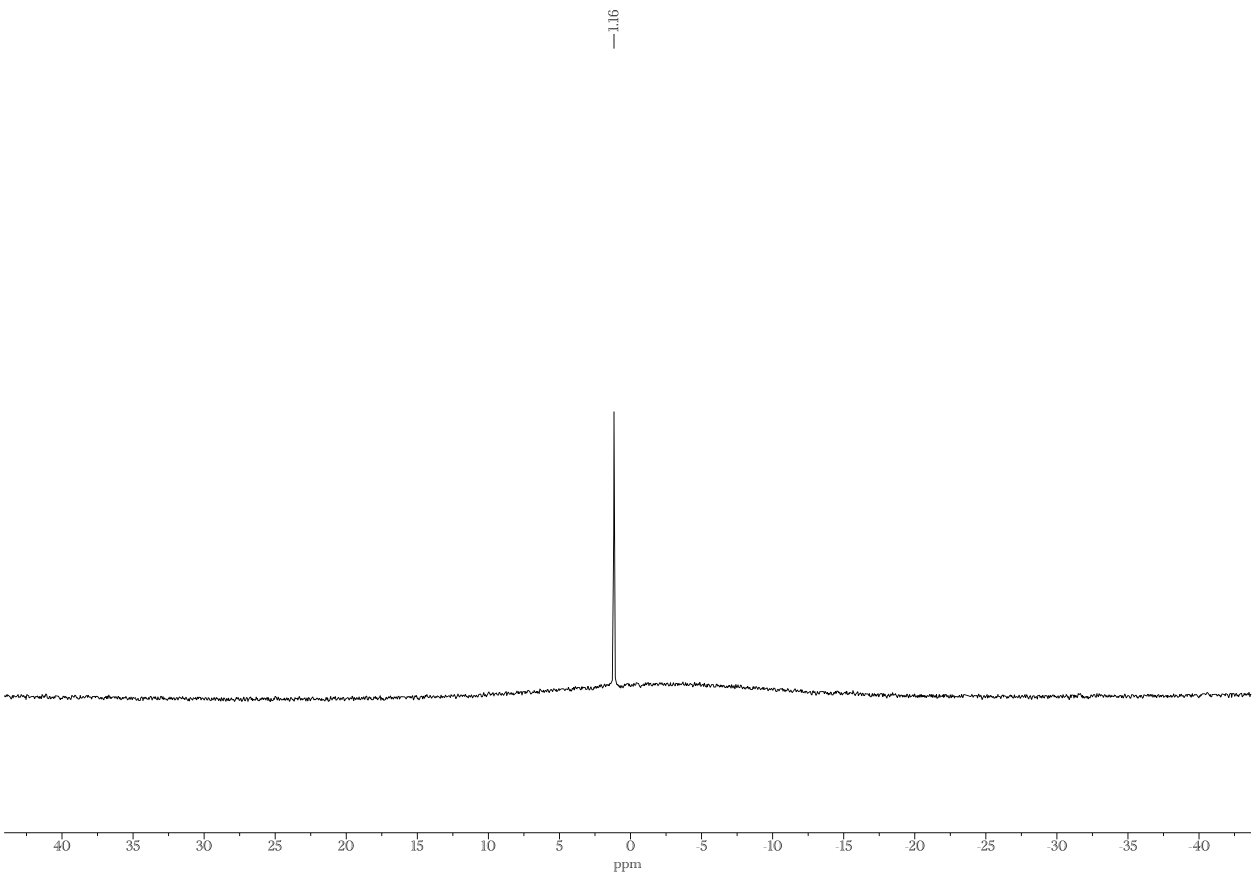

Figure S66: ^{11}B NMR (96 MHz, 296 K) spectrum of [**2•H$_2$O**][B(OC$_6$F$_5$)$_4$] in CD$_2$Cl$_2$.

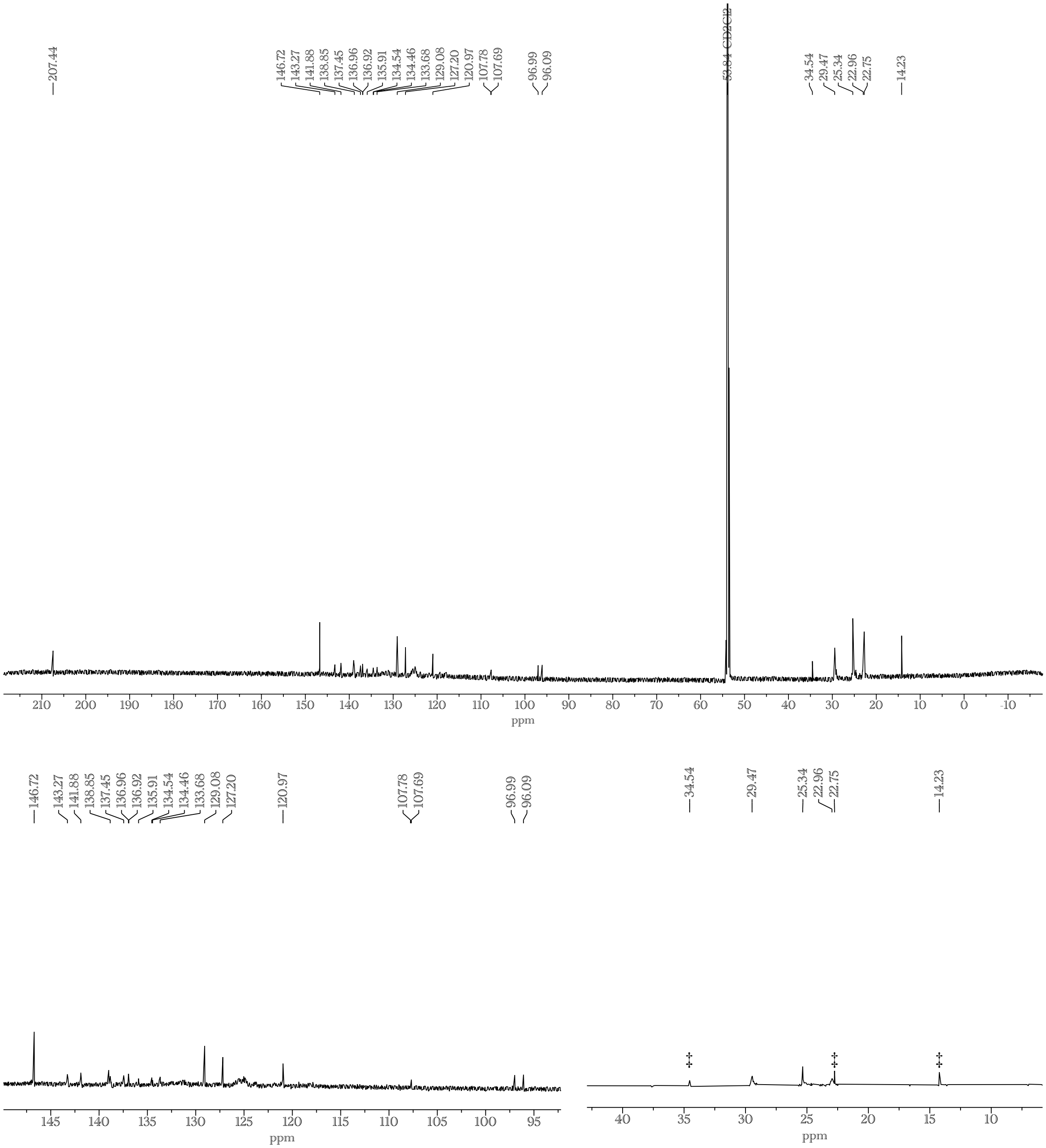

Figure S67: $^{13}C\{^1H\}$ NMR (176 MHz, 296 K) spectrum of [**2•H$_2$O**][B(OC$_6$F$_5$)$_4$] in CD_2Cl_2. . ‡ corresponds to trace pentane.

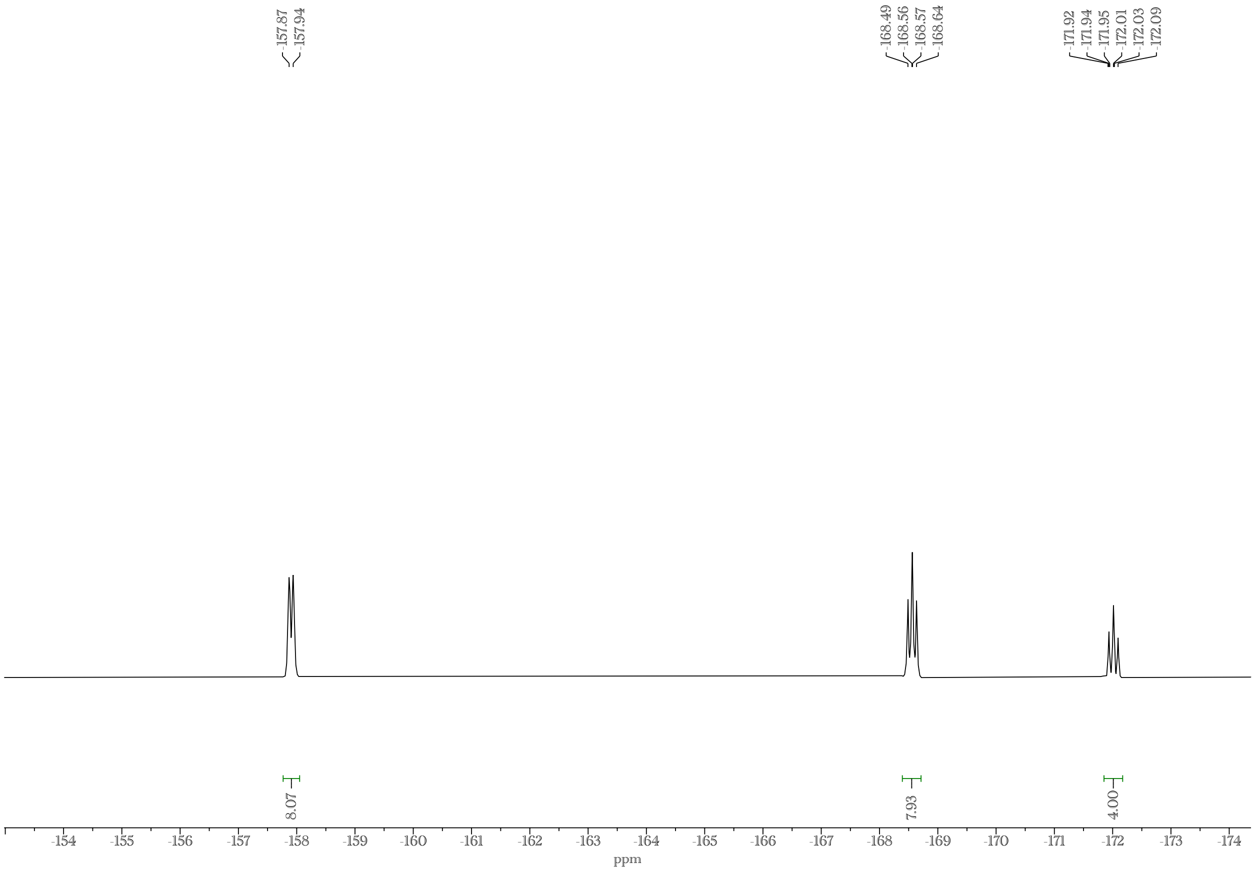

Figure S68: ^{19}F NMR (282 MHz, 296 K) spectrum of [**2•H$_2$O**][$B(OC_6F_5)_4$] in CD_2Cl_2.

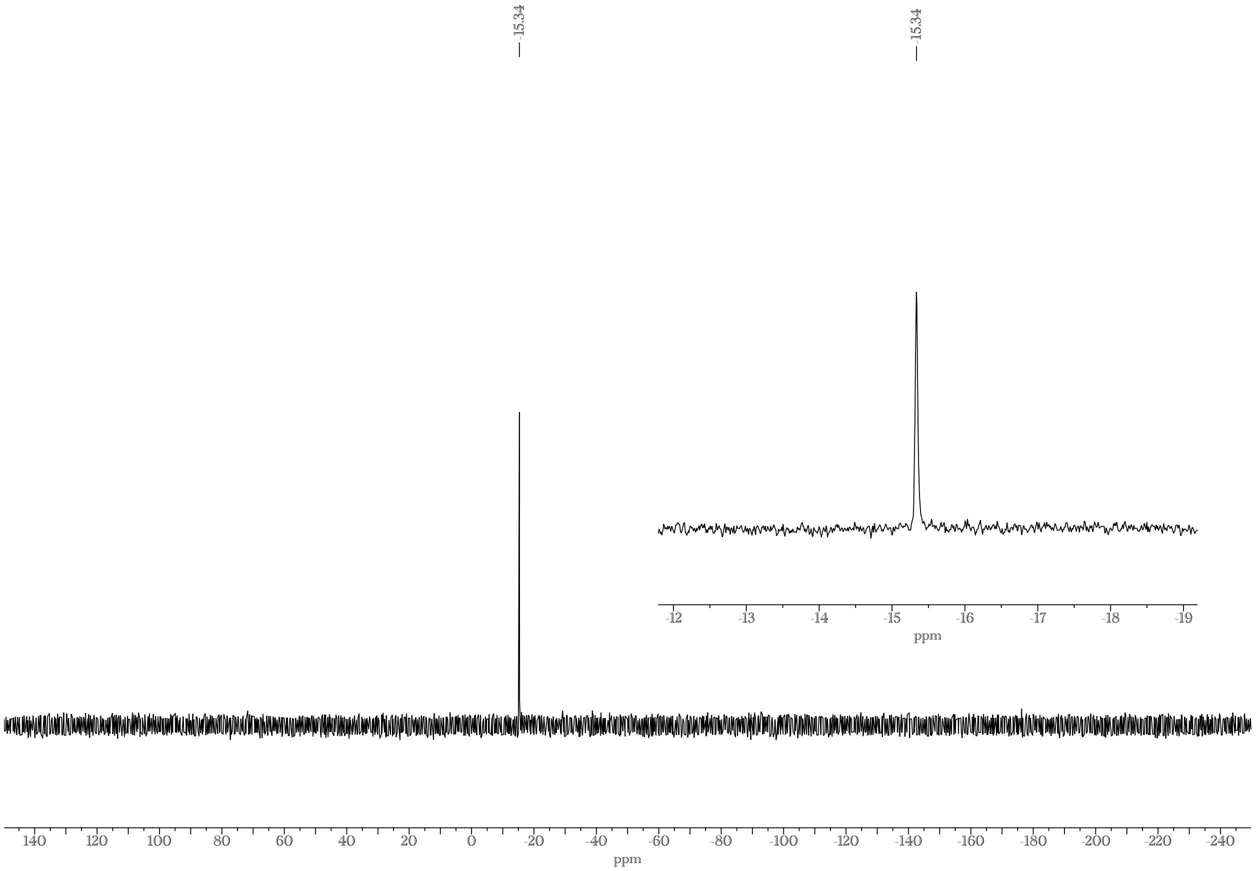

Figure S69: ^{31}P NMR (121 MHz, 296 K) spectrum with zoom in of [**2•H$_2$O**][B(OC$_6$F$_5$)$_4$] in CD$_2$Cl$_2$.

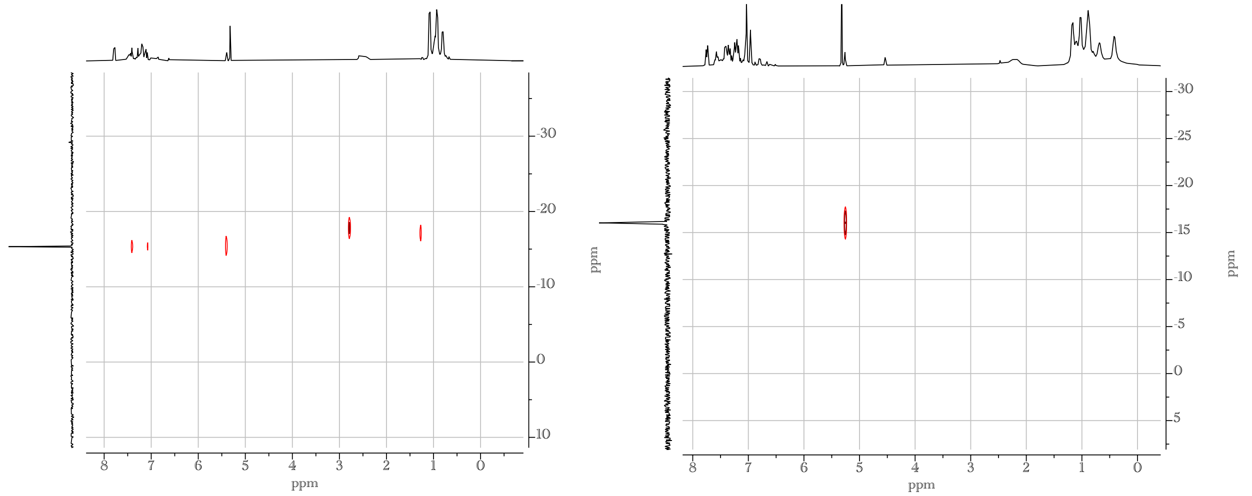

Figure S70: $^{1}H/^{31}P$ HMBC NMR spectrum of [**2•H$_2$O**][B(OC$_6$F$_5$)$_4$] at room temperature (left) vs $^{1}H/^{31}P$ HMBC NMR spectrum at 218K. In both cases, correlations between ^{31}P and the N-H proton are not detected.

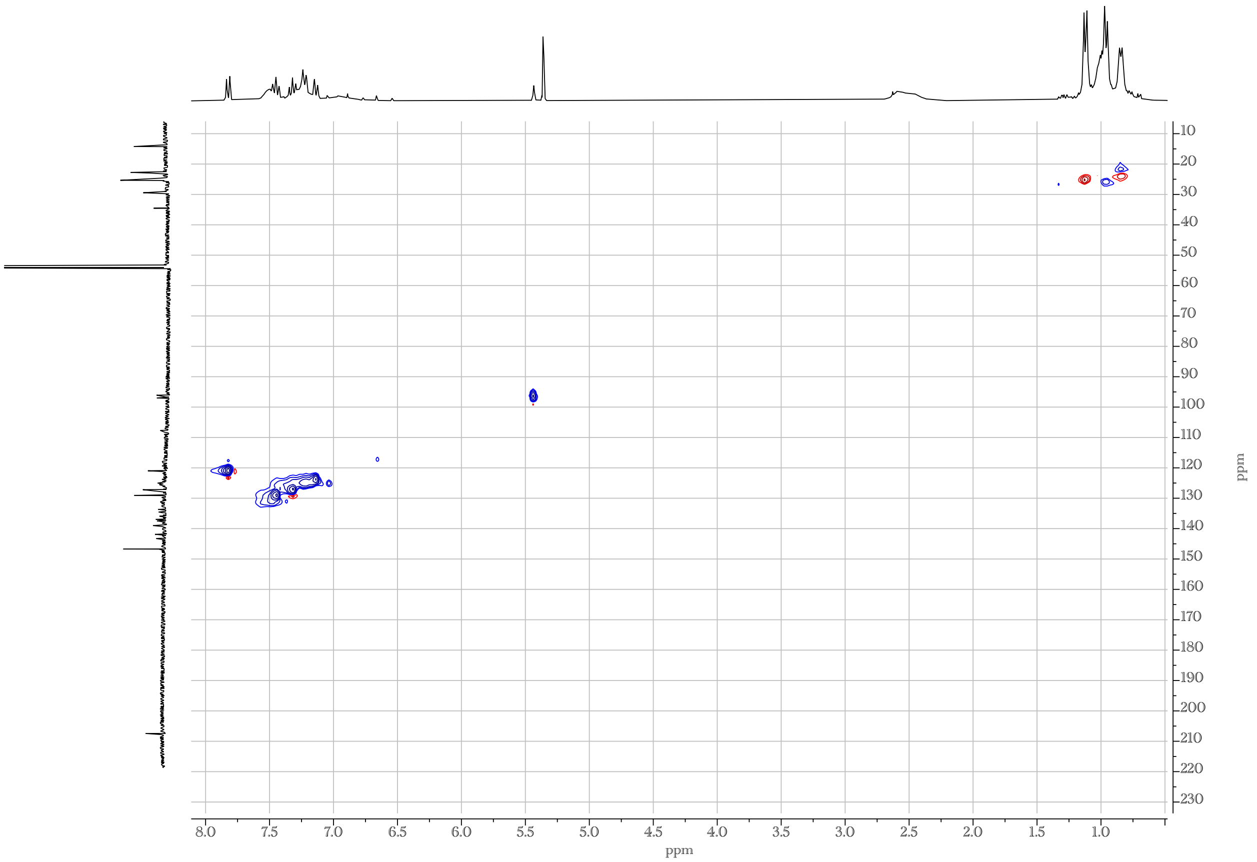

Figure S71: $^1H/^{13}C\{^1H\}$ HSQC NMR spectrum of [**2•H$_2$O**][B(OC$_6$F$_5$)$_4$] in CD$_2$Cl$_2$.

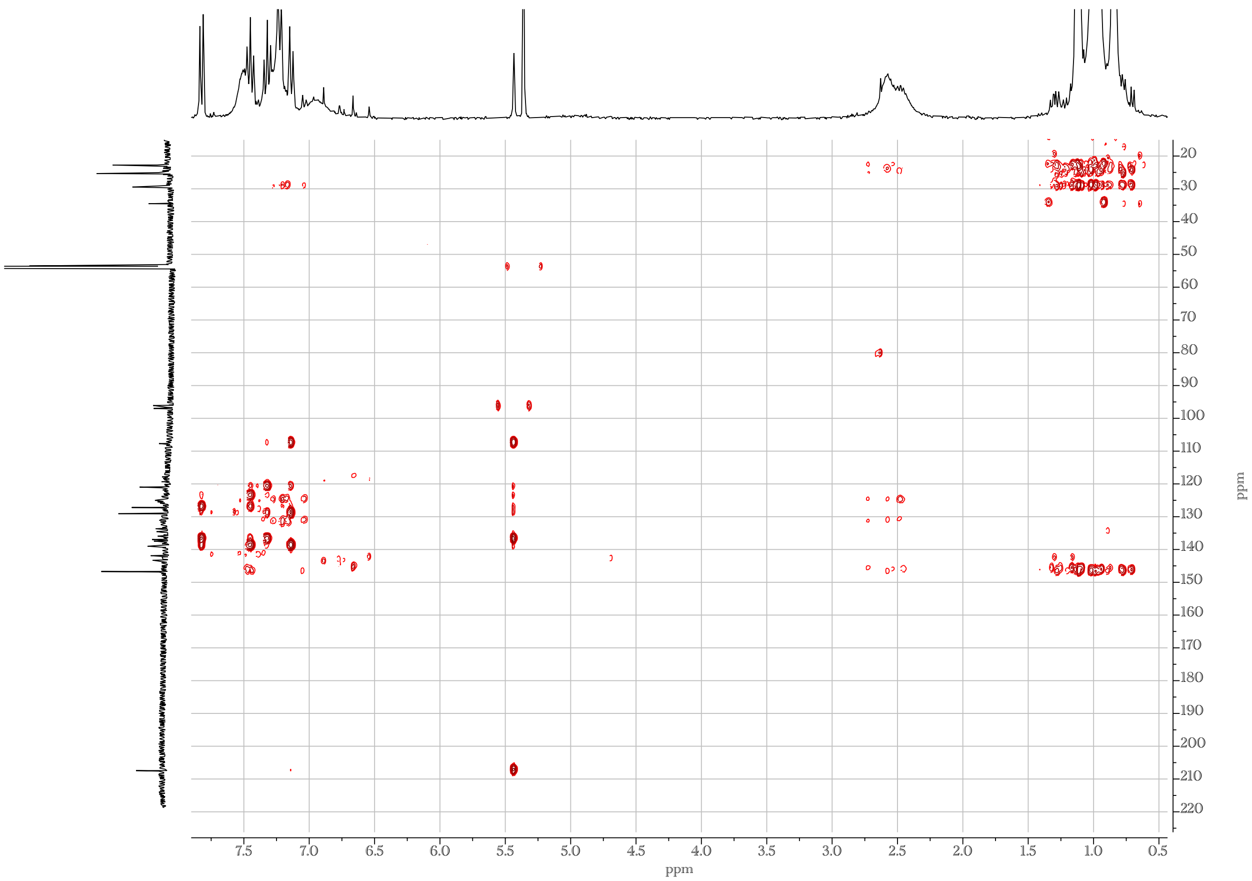

Figure S72: $^{1}H/^{13}C\{^{1}H\}$ HMBC NMR spectrum of [**2•H$_2$O**][B(OC$_6$F$_5$)$_4$] in CD$_2$Cl$_2$.

3.0 X-ray Diffraction Studies

Single-crystal X-ray diffraction data were collected on a Bruker APEX-II CCD detector or with a Bruker D8 QUEST PHOTON III C14, both using Mo-Kα radiation sources (λ = 0.71073 Å). Crystals were selected under oil, mounted on either glass fibre or nylon loops and then immediately placed in a cold stream of N_2 on a diffractometer. The APEX2[46] and APEX4[47] software was used to operate the diffractometers. The data was integrated with SAINT15 [48] and corrected for absorption effects based on Gaussian numerical integration and scaled with SADABS.[49] Using Olex2,[50] the structures were solved with the Superflip[51] Olex2.solve [52], ShelXS [53], ShelXD [53] or ShelXT[54] using charge flipping, direct, or dual methods. The refinement was done with ShelXL[53] using Least Squares minimization or Olex2.refine[52] using Gauss-Newton minimization.

Ellipsoids are drawn at 50% probability and for clarity hydrogen atoms are omitted. If present, solvent molecules and disordered parts are shown for a complete structural depiction.

Crystallographic data has been deposited with the Cambridge Crystallographic Data Centre as supplementary publication no. CCDC-2371603 (**1**), CCDC-2371604 ([**2**][$B(OC_6F_5)_4$]), CCDC-2371602 [**2**$^{\mathbf{cyclo}}$][$B(OC_6F_5)_4$], CCDC-2371601 [**2•DMAP**][$B(OC_6F_5)_4$]. These data can be obtained free of charge via www.ccdc.cam.uk/data_request/cif (or from the CCDC, 12 Union Road, Cambridge CB2 1EZ, UK; fax: (+44) 1223-336-033; or deposit@ccdc.cam.ac.uk).

3.1 SCXRD Analysis of 1

Yellow single crystals of **1** obtained by storing a concentrated *n*-hexane solution at -40 °C. **1** crystallizes in the $P2_1/c$ space group and contains four molecule per unit cell. The assymetric unit contains one molecule of **1.**

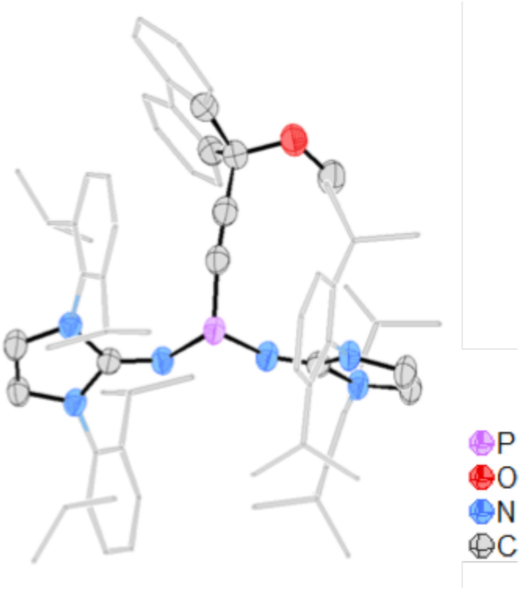

Figure S73: Molecular view of phosphine **1**. Dipp substituents and the fluorenyl moiety shown in wireframe.

Table S1: Crystal Structure Data of Compound **1**

CCDC deposition number	2371603	ρ_{calc}g/cm^3	1.143
Empirical formula	$C_{70}H_{83}N_6OP$	μ/mm^{-1}	0.092
Formula weight	1055.39	F(000)	2272
Temperature/K	173.00K	Crystal size/mm^3	0.18 × 0.13 × 0.015
Crystal system	Monoclinic	Radiation	MoK$_\alpha$ (λ = 0.71073)
Space group	$P2_1/c$	2Θ range for data collection/°	1.977 to 25.117
a/Å	23.702(3)	Index ranges	-28 ≤ h ≤ 28, -14 ≤ k ≤ 14, -26 ≤ l ≤ 25
b/Å	12.4334(14)	Reflections collected	102976
c/Å	21.898(2)	Independent reflections	10673 [R_{int} = 0.1083, R_{int} = 0.0744]
α/°	90	Data/restraints/parameters	10673/0/721
β/°	108.088(3)	Goodness-of-fit on F^2	1.016
γ/°	90	Final R indexes [I>2σ (I)]	R_1 = 0.0597, wR_2 = 0.1493
Volume/Å^3	6134.3(12)	Final R indexes [all data]	R_1 = 0.1086, wR_2 = 0.1804
Z	4	Largest diff. peak/hole/ eÅ^{-3}	0.35/-0.30

3.2 SCXRD Analysis of [2][B(OC_6F_5)$_4$]

Red single crystals of [**2**][B(OC_6F_5)$_4$] were obtained from a mixed CH_2Cl_2/ pentane solution which was stored at -40 °C. [**2**][B(OC_6F_5)$_4$] crystalizes in the orthorhombic Pccn space group and contains one molecule per unit cell.

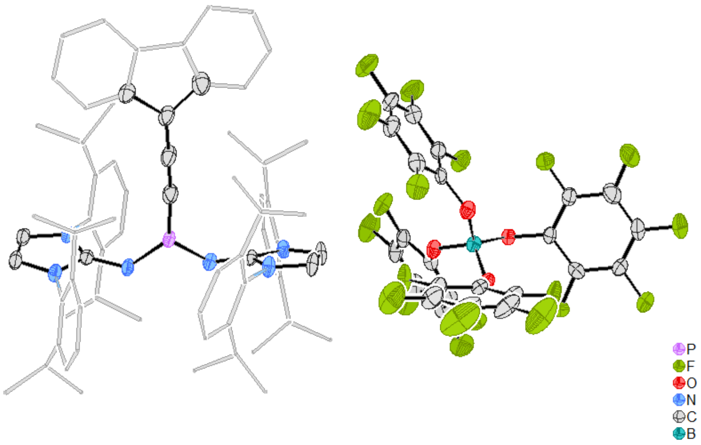

Figure S74: Molecular view of allenylidene phosphonium salt [**2**][B(OC_6F_5)$_4$]. Solvent mask program implemented in OLEX2 was applied to *n*-pentane.

Table S2: Crystal Structure Data of Compound [**2**][B(OC_6F_5)$_4$] • 3(n-pentane)

CCDC	2371604	ρ_{calc}g/cm^3	1.355
Empirical formula	$C_{108}H_{116}BF_{20}N_6O_4P$	μ/mm^{-1}	0.124
Formula weight	1983.84	F(000)	8304.0
Temperature/K	173.00K	Crystal size/mm^3	0.015 × 0.01 × 0.005
Crystal system	Orthorhombic	Radiation	MoK$_\alpha$ (λ = 0.71073)
Space group	*P*ccn	2Θ range for data collection/°	3.38 to 52.744
a/Å	25.7844(10)	Index ranges	$-32 \leq h \leq 32$, $-39 \leq k \leq 39$, $30 \leq l \leq 27$
b/Å	31.3106(13)	Reflections collected	254219
c/Å	24.0922(10)	Independent reflections	19878 [R_{int} = 0.1000, R_{sigma} = 0.0452]
α/°	90	Data/restraints/parameters	19878/0/1142
β/°	90	Goodness-of-fit on F^2	1.138
γ/°	90	Final R indexes [I>2σ (I)]	R_1 = 0.0956, wR_2 = 0.1972
Volume/Å^3	19450.2(14)	Final R indexes [all data]	R_1 = 0.1180, wR_2 = 0.2083
Z	8	Largest diff. peak/hole/ eÅ^{-3}	0.41/-0.51

3.3 SCXRD Analysis of [**2**cyclo][B(OC$_6$F$_5$)$_4$]

Colourless single crystals of [**2**cyclo][B(OC$_6$F$_5$)$_4$] were obtained by slow evaporation of a concentrated CH_2Cl_2 [**2**cyclo][B(OC$_6$F$_5$)$_4$] crystalizes in the P2$_1$/c space group and contains one molecule per unit cell.

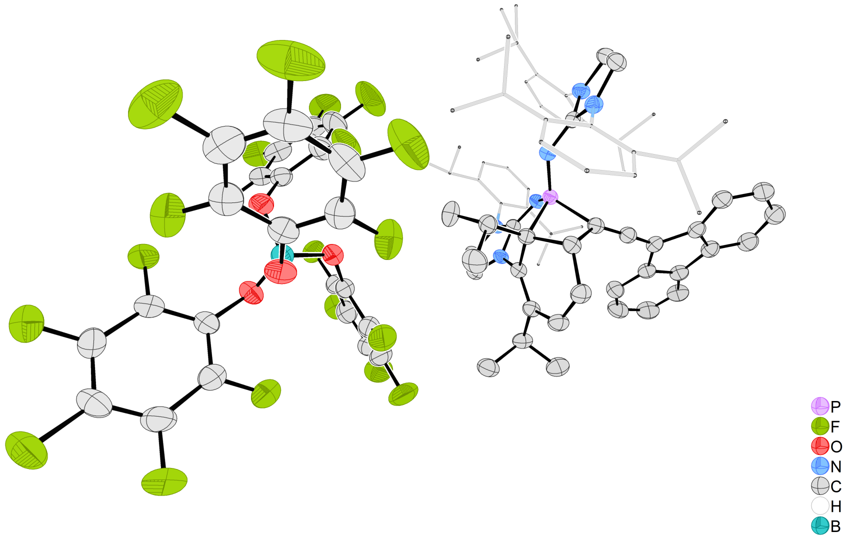

Figure S75: Molecular view of [**2**cyclo][B(OC$_6$F$_5$)$_4$].

Table S3: Crystal Structure Data of Compound [**2**cyclo][B(OC$_6$F$_5$)$_4$]

CCDC	2371601	ρ_{calc}g/cm^3	1.403
Empirical formula	$C_{93}H_{80}BF_{20}N_6O_4P$	μ/mm^{-1}	0.134
Formula weight	1767.71	F(000)	3648
Temperature/K	173.00K	Crystal size/mm^3	0.186 × 0.088 × 0.045
Crystal system	Monoclinic	Radiation	MoK$_\alpha$ (λ = 0.71073)
Space group	*P*2$_1$/*c*	2Θ range for data collection/°	3.614 to 50.866
a/Å	14.4058(6)	Index ranges	$-17 \le h \le 17$, $-26 \le k \le 26$, $-32 \le l \le 32$
b/Å	22.2218(11)	Reflections collected	145065
c/Å	26.6962	Independent reflections	15419 [R_{int} = 0.0983, R_{sigma} = 0.0518]
α/°	90	Data/restraints/parameters	15419/0/1153
β/°	101.691(2)	Goodness-of-fit on F^2	1.008
γ/°	90	Final R indexes [I>2σ (I)]	R_1 = 0.0488, wR_2 = 0.1131
Volume/Å^3	8368.8(7)	Final R indexes [all data]	R_1 = 0.0869, wR_2 = 0.1350
Z	4	Largest diff. peak/hole/ eÅ^{-3}	0.28/-0.30

3.4 SCXRD Analysis of [**2•DMAP**][B(OC_6F_5)$_4$]

Orange single crystals of [**2•DMAP**][B(OC_6F_5)$_4$] were obtained by slow evaporation of a concentrated ether solution. [**2•DMAP**][B(OC_6F_5)$_4$] crystalizes in the $P2_1/c$ space group and contains one molecule of [**2•DMAP**][B(OC_6F_5)$_4$] and one molecule of diethyl ether per unit cell.

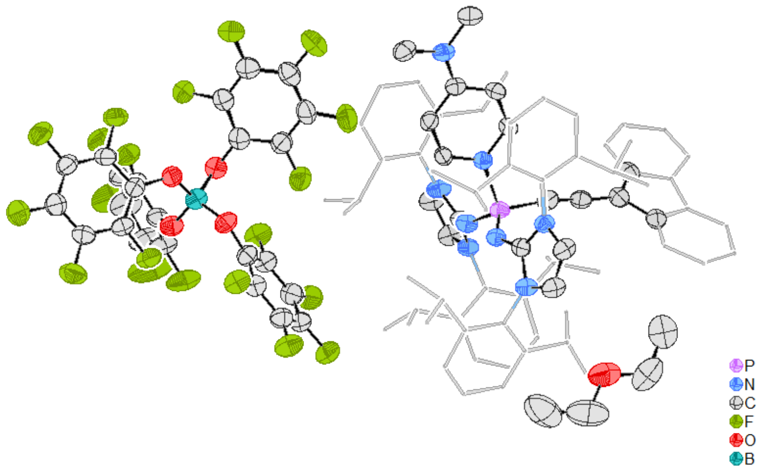

Figure S76: Molecular view of [**2•DMAP**][B(OC_6F_5)$_4$]. Solvent mask program implemented in OLEX2 was applied to diethyl ether.

Table S4: Crystal Structure Data of Compound [**2•DMAP**][B(OC_6F_5)$_4$]

CCDC	2371601	ρ_{calc}g/cm^3	1.358
Empirical formula	$C_{100}H_{90}BF_{20}N_8O_4P$ x $2C_4H_{10}O$	μ/mm^{-1}	0.125
Formula weight	2037.81	F(000)	2124
Temperature/K	173.00K	Crystal size/mm^3	0.14 × 0.09 × 0.06
Crystal system	Triclinic	Radiation	MoK$_\alpha$ (λ = 0.71073)
Space group	*P*-1 (no. 2)	2Θ range for data collection/°	2.246 to 25.110
a/Å	14.223(3)	Index ranges	$-16 \le h \le 16$, $-21 \le k \le 21$, $-24 \le l \le 24$
b/Å	17.793(4)	Reflections collected	121104
c/Å	20.317(4)	Independent reflections	17684 [R_{int} = 0.0714, R_{sigma} = 0.0517]
α/°	85.519 (5)	Data/restraints/parameters	17684/0/1272
β/°	76.625 (4)	Goodness-of-fit on F^2	1.032
γ/°	87.233 (5)	Final R indexes [I>2σ (I)]	R_1 = 0.0580, wR_2 = 0.1573
Volume/Å^3	4984.5 (18)	Final R indexes [all data]	R_1 = 0.0977, wR_2 = 0.1835
Z	2	Largest diff. peak/hole/ eÅ^{-3}	0.373/-0.457

4. Computational details:

All Density Functional Theory (DFT) calculations have been performed using the Gaussian 16 program package.[55] All geometry optimizations were performed at B3LYP [56] level of theory using Grimme's D3 dispersion model with Becke-Johnson Damping (D3-BJ) [57] using def2-TZVP [58] basis sets employing a universal solvation model based on density (SMD) for dichloromethane.[59] Frequency calculations confirmed with no imaginary frequencies confirmed the attainment of the stationary points on the potential energy surface (PES). UV-Vis absorption spectrum was simulated at B3LYP/6-311+G(d) using SMD for CH_2Cl_2. Transition state geometry produced a single imaginary frequency confirming a first-order saddle point on the PES. The Cartesian coordinates and energies of all optimized molecules are provided in a supplementary *.xyz file.

4.1 NBO, Mayer Bond Order, and Hirshfeld Charges

The % s/p-character of the phosphorus atom and cumulenic carbon atoms of [**2**]$^+$ were calculated by the natural bond orbital analyses using NBO 3.1 module as implemented in the Gaussian 16 programs in B3LYP/Lanl2dz method. [60] The Hirshfeld charges and the Mayer bond orders of [**2**]$^+$ were calculated using Multiwfn 3.7 software package.[61].Molecular orbitals and the electrostatic potential map were visualized using GaussView 6 program.

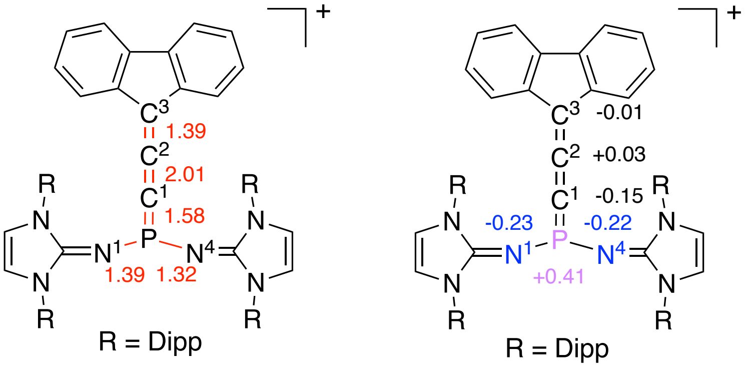

Figure S77: Mayer bond orders and Hirshfeld charges determined at B3LYP(D3-BJ)/def2-TZVP.

Table S5: NBO analysis of [**2**]$^+$, with Mayer bond orders, determined at B3LYP(D3-BJ)/def2-TZVP.

	Bond lengths (Å)		NBO calculations	
	Calculated	*Experimental*	Orbital contribution (%)	Mayer bond order
P(1) – C(1)	1.626	1.625	σ: $P_1(s^{35.19}p^{63.96}) – C_1(s^{47.01}p^{52.78})$<br>π: $P_1(s^{0.14}p^{98.56}) – C_1(s^{0.14}p^{99.60})$	1.58
C(1) – C(2)	1.264	1.250	σ: $C_1(s^{52.46}p^{47.34}) – C_2(s^{49.94}p^{49.90})$<br>π: $C_1(s^{0.19}p^{99.68}) – C_2(s^{0.02}p^{99.87})$	2.01
C(2) – C(3)	1.338	1.334	σ: $C_2(s^{49.98}p^{49.84}) – C_3(s^{36.52}p^{63.40})$<br>π: $C_2(s^{0.01}p^{99.84}) – C_3(s^{0.00}p^{99.94})$	1.39
P(1) – N(1)	1.585	1.557	σ: $P_1(s^{32.08}p^{67.02}) – N_4(s^{28.83}p^{70.73})$	1.39
P(1) – N(4)	1.592	1.560	σ: $P_1(s^{32.31}p^{66.77}) – N_1(s^{32.57}p^{67.05})$	1.32

Table S6: Hirshfeld Charges of [**2**]$^+$, determined at B3LYP(D3-BJ)/def2-TZVP.

**Atoms**	**Hirshfeld charges**
P(1)	(+) 0.41
N(1)	(-) 0.23
N(4)	(-) 0.22
C(1)	(-) 0.15
C(2)	(+) 0.03
C(3)	(-) 0.01

4.2 Electrostatic Potential Map and Molecular Orbitals

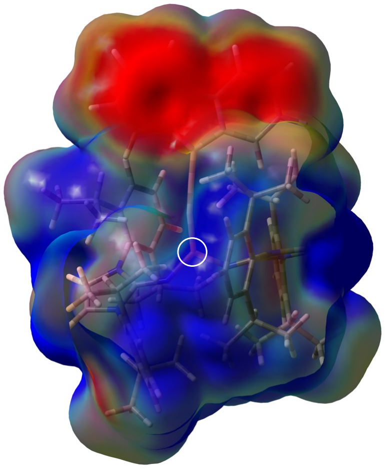

Figure S78: Electrostatic potential map of [**2**]$^{+}$ determined at B3LYP(D3-BJ)/def2-TZVP visualized with Gaussview 6.

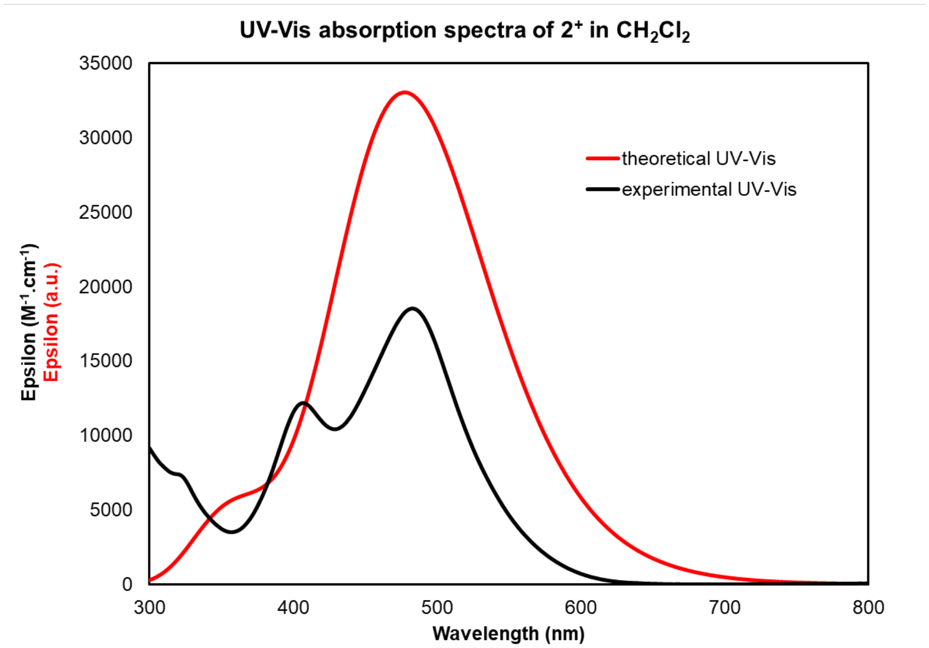

Figure S79: Experimental (in black, 5E-5 M in CH_2Cl_2) and simulated (in red, using SMD for CH_2Cl_2) UV-Vis absorption spectra of **2**$^{+}$.

Table S7: Time dependant Density Functional Theory (TD-DFT) simulation of **2**$^+$ in B3LYP/6-311+G(d) using SMD CH_2Cl_2 solvation.

Compound	Theoretical (experimental) λ in nm	Oscillator Strength	Transition Assignment
[**2**$^+$]	478.3 (483.0)	0.8074	275 (HOMO) --> 276 (LUMO)
	419.9 (407.0)	0.0022	274 (HOMO-1) --> 276 (LUMO)

Table S8: Selected molecular orbitals of [**2**]$^+$ at B3LYP(D3-BJ)/def2-TZVP visualized with GaussView 6 at isovalue = 0.02.

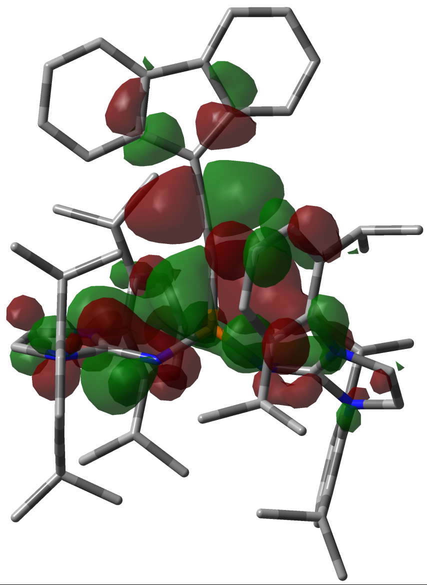	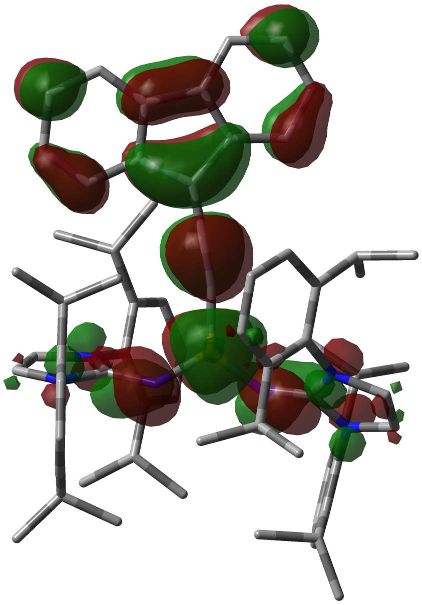
LUMO + 1 (-3.51 eV)	LUMO (-4.51 eV)

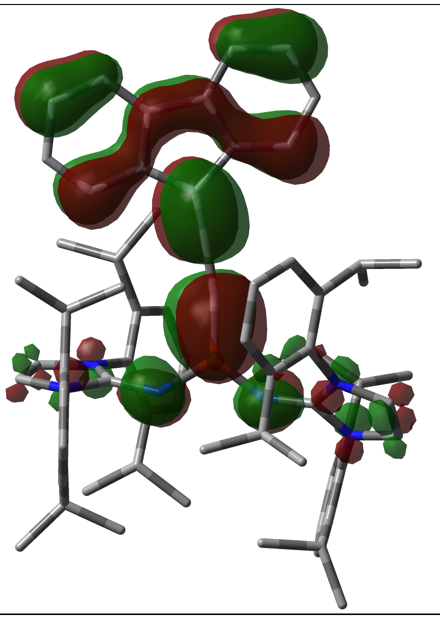	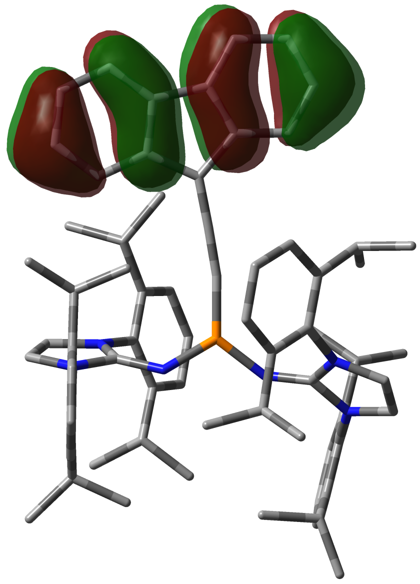
HOMO (-7.38 eV)	HOMO-1 (-7.90 eV)
HOMO-LUMO Gap = 2.87 eV	
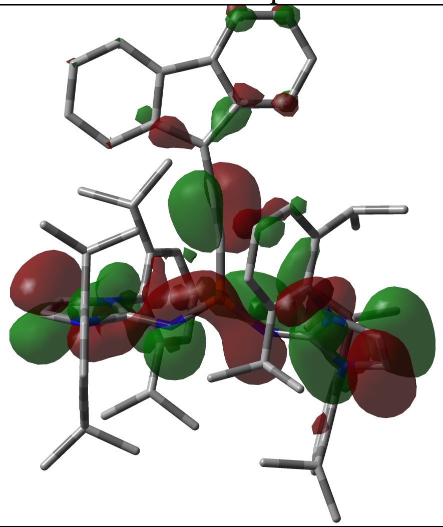	
HOMO-2	

4.3 Potential Energy Landscapes

Model systems [**2**]$^+_{\mathbf{Model}}$ and [**2**$^{\mathbf{cyclo}}$]$^+_{\mathbf{Model}}$ (three Dipp groups substituted with methyl groups on the endocyclic nitrogen atoms of the N-heterocyclic imine substituents) of the respective phosphonium cations [**2**]$^+$ and [**2**$^{\mathbf{cyclo}}$]$^+$ were calculated with B3LYP(D3-BJ)/ def2-TZVP.

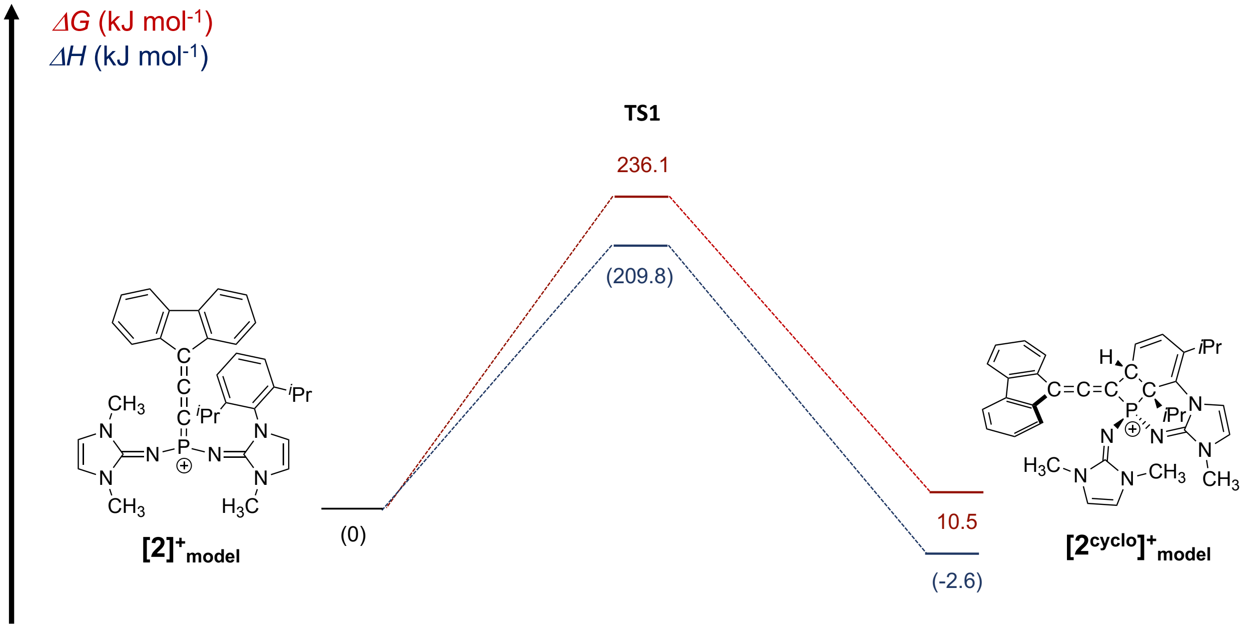

Figure S80: Energy profile (free energy in red, enthalpy in blue) of the reaction path [**2**]$^+_{\mathbf{model}}$→TS1→[**2**$^{\mathbf{cyclo}}$]$^+_{\mathbf{model}}$ determined at B3LYP(D3-BJ)/def2-TZVP in vacuum.

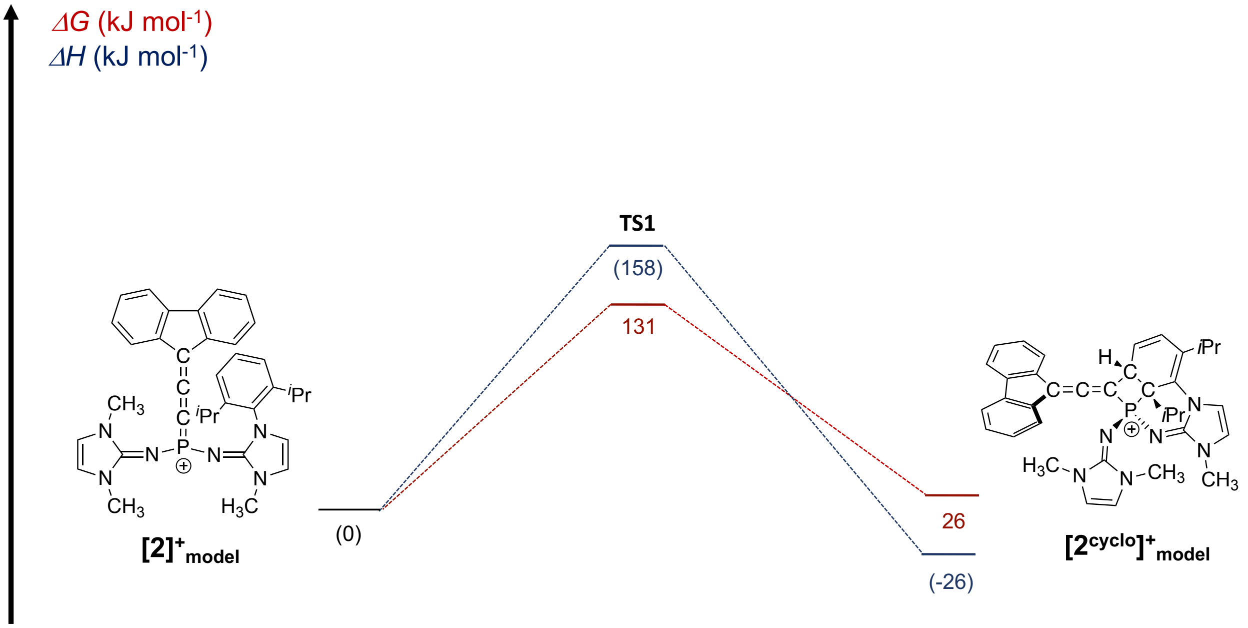

Figure S81: Energy profile (free energy in red, enthalpy in blue) of the reaction path [**2**]$^+_{\mathbf{model}}$→TS1→[**2**$^{\mathbf{cyclo}}$]$^+_{\mathbf{model}}$ determined at B3LYP(D3-BJ)/def2-TZVP in CH_2Cl_2 (SMD solvation model).

Table S9: Optimized geometries of the reaction [**2**]$^{+}$$_{\textbf{Model}}$ (left)→**TS1**(middle)→[**2**$^{\textbf{cyclo}}$]$^{+}$$_{\textbf{Model}}$ (right) obtained at B3LYP (D3-BJ)/ def2-TZVP level of theory.

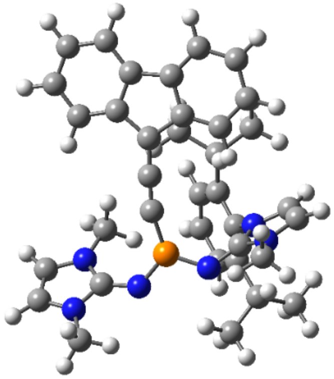	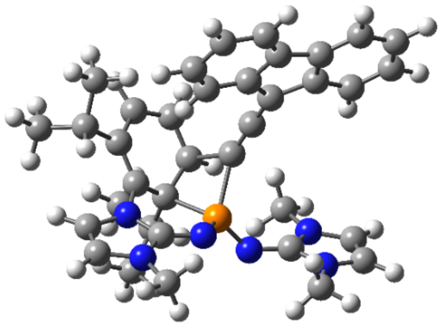	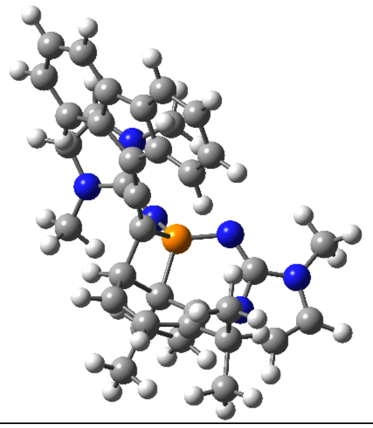
[**2**]$^{+}$ $_{\textbf{model}}$	**TS1**	[**2**$^{\textbf{cyclo}}$]$^{+}$ $_{\textbf{model}}$

Table S10: Thermochemistry values for, [**2**]$^{+}$$_{\textbf{model}}$, **TS1** and [**2**$^{\textbf{cyclo}}$]$^{+}$$_{\textbf{model}}$ in vacuum.

Vacuum	ΔG		ΔH	
	kcal/mol	kJ/mol	kcal/mol	kJ/mol
[**2**]$^{+}$	0	0	0	0
TS1	56.5	236.1	50.2	209.8
[**2**$^{\textbf{cyclo}}$]$^{+}$	2.5	10.5	-0.6	-2.6

Table 11: Thermochemistry values for, [**2**]$^{+}$$_{\textbf{model}}$, **TS1** and [**2**$^{\textbf{cyclo}}$]$^{+}$$_{\textbf{model}}$ in CH_2Cl_2.

DCM	ΔG		ΔH	
	kcal/mol	kJ/mol	kcal/mol	kJ/mol
[**2**]$^{+}$	0	0	0	0
TS1	31.4	131.2	37.7	157.4
[**2**$^{\textbf{cyclo}}$]$^{+}$	6.3	26.2	-6.3	-26.2

Calculated fluoride ion affinity (FIA) of [2]$^{+}$

The fluoride ion affinity (FIA) of [**2**]$^{+}$ was calculated in the gas phase and in dichloromethane. For the latter the SMD (dichloromethane) solvent correction was applied. The FIAs were calculated according to the reported procedure by Christe [62] anchored to COF_2.

Table S12: Fluoride ion affinities of [**2**]$^{+}$ in kJ/mol, determined by the Christe method**.**

Method:	Gas phase	Solvated, SMD (CH_2Cl_2)
Reference:	COF_2	COF_2
[**2**]$^{+}$	655	366

5.0 References

[41] P. Löwe, M. A. Wünsche, F. R. S. Purtscher, J. Gamper, T. S. Hofer, L. F. B. Wilm, M. B. Röthel, F. Dielmann, *Chem. Sci.* **2023**, 14, 7928-7935.

[42] D. Naumann, H. Butler, R. Gnann, *Z. Anorg. Allg. Chem.* **1992**, 618, 74-76.

[43] a) U. Mayer, V. Gutmann, W. Gerger, *Monatsh. Chem.* **1975**, 106, 1235-1257; b) I. B. Sivaev, V. I. Bregadze, *Coord. Chem. Rev.* **2014**, 270-271, 75-88; c) M. A. Beckett, G. C. Strickland, J. R. Holland, K. Sukumar Varma, *Polymer.* **1996**, 37, 4629-4631.

[44] G. C. Welch, L. Cabrera, P. A. Chase, E. Hollink, J. D. Masuda, P. Wei, D. W. Stephan, *Dalton Trans.* **2007**, 3407–3414.

[45] M. A. Wünsche, T. Witteler, F. Dielmann, *Angew. Chem. Int. Ed.* **2018**, 57, 7234-7239.

[46] APEX2 Version 2.1 – 0; Bruker AXS Inc. Madison, **2004**.

[47] APEX4; Bruker AXS Inc. Madison, **2021**.

[48] SAINT version 7.46a, Bruker AXS Inc. Madison **2004**.

[49] G. Sheldrick GM SADABS. University of Göttingen, Göttingen **1996**.

[50] O. V. Dolomanov, L. J. Bourhis, R. J. Gildea, J. A. K. Howard and H. Puschmann, *J. Appl. Crystallogr.* **2009**, 42, 339–341.

[51] a) L. Palatinus, G. Chapuis, *J. Appl. Cryst.* **2007**, 40, 786-790; b) L. Palatinus, A. van der Lee, *J. Appl. Cryst.* **2008**, 41, 975-984; c) L. Palatinus, S. J. Prathapa, S. van Smaalen, *J. Appl. Cryst.* **2012**, 45, 575-580.

[52] L. J. Bourhis; O. V. Dolomanov; R. J. Gildea; J. A. K. Howard; H. Puschmann, *Acta crystallogr. A*. **2015**, 71, 59–75.

[53] G. M. Sheldrick, *Acta crystallogr. A*. **2008**, 64, 112–122.

[54] G. M. Sheldrick, *Acta crystallogr. A*. **2015**, 71, 3–8.

[55] Gaussian 16, Revision C.01, M. J. Frisch, G. W. Trucks, H. B. Schlegel, G. E. Scuseria, M. A. Robb, J. R. Cheeseman, G. Scalmani, V. Barone, G. A. Petersson, H. Nakatsuji, X. Li, M. Caricato, A. V. Marenich, J. Bloino, B. G. Janesko, R. Gomperts, B. Mennucci, H. P. Hratchian, J. V. Ortiz, A. F. Izmaylov, J. L. Sonnenberg, D. Williams-Young, F. Ding, F. Lipparini, F. Egidi, J. Goings, B. Peng, A. Petrone, T. Henderson, D. Ranasinghe, V. G. Zakrzewski, J. Gao, N. Rega, G. Zheng, W. Liang, M. Hada, M. Ehara, K. Toyota, R. Fukuda, J. Hasegawa, M. Ishida, T. Nakajima, Y. Honda, O. Kitao, H. Nakai, T. Vreven, K. Throssell, J. A. Montgomery, Jr., J. E. Peralta, F. Ogliaro, M. J. Bearpark, J. J. Heyd, E. N. Brothers, K. N. Kudin, V. N.

Staroverov, T. A. Keith, R. Kobayashi, J. Normand, K. Raghavachari, A. P. Rendell, J. C. Burant, S. S. Iyengar, J. Tomasi, M. Cossi, J. M. Millam, M. Klene, C. Adamo, R. Cammi, J. W. Ochterski, R. L. Martin, K. Morokuma, O. Farkas, J. B. Foresman, and D. J. Fox, Gaussian, Inc., Wallingford CT, **2016**.

[56] a) A. D. Becke, *J. Chem. Phys*, **1993**, 98, 5648–5652; b) C. Lee, W. Yang, R. G. Parr, *Phys. Rev. B.* **1998**, 37, 785–789; c) S. H. Vosko, L. Wilk, M. Nusair, *Can. J. Phys*. **1980**, 58, 1200-1211; d) P. J. Stephens, F. J. Devlin, C. F. Chabalowski, M. J. Frisch, *J. Phys. Chem*. **1994**, 98, 11623–11627.

[57] a) L. Goerigk, S. Grimme, *J. Chem. Theory Comput.* **2011**, 7, 291–309; b) L. Goerigk, A. Hansen, C. Bauer, S. Ehrlich, A. Najibi, S. Grimme, *Phys. Chem. Chem. Phys.* **2017**, 19, 32184–32215; c) B. G. Johnson, M. J. Frisch, *J. Chem. Phys.* **1994**, 100, 7429–7442.

[58] a) F. Weigend, R. Ahlrichs, *Phys. Chem. Chem. Phys.* **2005**, 7, 3297–3305; b) F. Weigend, *Phys. Chem. Chem. Phys.* **2006**, 8, 1057–1065.

[59] A. V. Marenich, C. J. Cramer, D. G. Truhlar, *Phys. Chem. B*. **2009**, 113, 6378–6396.

[60] a) F. Weinhold, C. R. Landis (Eds.) Discovering Chemistry with Natural Bond Orbitals, John Wiley & Sons, Inc, Hoboken, NJ, USA, **2012**; b) J. P. Foster, F. Weinhold, *J. Am. Chem. Soc.* **1980**, 102, 7211–7218; c) A. E. Reed, R. B. Weinstock, F. Weinhold, *J. Chem. Phys.* **1985**, 83, 735–746; d) J. E. Carpenter, F. Weinhold, *J. Mol. Struct.: THEOCHEM*. **1988**, 169, 41–62; e) F. Weinhold, J. E. Carpenter, *The Structure of Small Molecules and Ions*, R. Naaman and Z. Vager, Springer US, Boston, MA, **1988**, pp. 227–236.

[61] T. Lu, F. Chen, *J. Comput. Chem.* **2012**, 33, 580–592.

[62] K. O. Christe, D. A. Dixon, D. McLemore, W. W. Wilson, J. A. Sheehy, J. A. Boatz, *J. Fluorine Chem*. **2000**, 101, 151–153.
